# Supplementary material for: High-throughput screening of caterpillars as a platform to study host–microbe interactions and enteric immunity
Source: Nat Commun. 2022 Nov 24;13:7216. doi: 10.1038/s41467-022-34865-7 (PMC9700799; doi:10.1038/s41467-022-34865-7)

Supplementary Information

High-throughput Screening of Caterpillars as a Platform to Study Host-microbe Interactions and Enteric Immunity

Anton G. Windfelder, Frank H. H. Müller, Benedict Mc Larney, Michael Hentschel, Anna Christina Böhringer, Christoph-Rüdiger von Bredow, Florian H. Leinberger, Marian Kampschulte, Lorenz Maier, Yvette M. von Bredow, Vera Flocke, Hans Merzendorfer, Gabriele A. Krombach, Andreas Vilcinskas, Jan Grimm, Tina E. Trenczek & Ulrich Flögel

Supplementary Methods
Supplementary Tables S1 to S17
Supplementary Figures S1 to S26

Supplementary Materials and Methods

Animal rearing preparation and ethics

The *Manduca sexta* larvae were reared at 24 °C with a 16 h photoperiod and were fed on a modified artificial diet[1] without fixatives (Details of the *Manduca sexta* diet are given in the source data). To establish imaging procedures, larvae were fed with artificial diet cuboids (7 × 7 × 4 mm) immersed in 0.3% (w/v) commercially available *Bacillus thuringiensis* (Bt) subsp. *aizawai* suspension (2.7 × 10^8 cells/ml) (Xentari, Neudorff, #00592), or containing 5% (w/v) dextran sodium sulfate (DSS, MP Biomedicals, #0216011025), *E. coli* (DH5α strain, New England Biolabs, Ipswich, MA, #C2987H) with 2.7 × 10^8 cells/ml (*E. coli* control), without any additions (control), or Bt with two concentrations of gentamicin (0.5 mg/ml or 1.0 mg/ml, Sigma-Aldrich, St. Louis, MO, #G1914) for 12 h.

For uracil experiments, the larvae were fed with food cuboids submerged in 0.1 M uracil (Carl Roth, Karlruhe, Germany, #7288.2) with or without DPI (65 µM, Cayman Chemical, Ann Arbor, MI, #81050) and NAC (72 µM, Carl Roth, Karlruhe, Germany, #4126.1) or with a normal diet (control) for 12 h. Further animals were fed on a regular diet containing 1.0 M uracil. One cuboid was used per animal. They were starved 1 h before feeding, and no additional food was applied. For the dexamethasone rescue experiment, larvae were fed as indicated above and injected with 100 µg dexamethasone (Sigma-Aldrich, St. Louis, MO, #D4902) in 0.1 ml 0.9 % NaCl and exposed to a regular control diet. The inflammation group was exposed to 0.1 M uracil treatment as indicated above and injected with 0.1 ml 0.9 % NaCl. The rescue group was exposed to 0.1 M uracil treatment and injected with 100 µg dexamethasone in 0.1 ml 0.9 % NaCl. CT imaging was done after 12 h of exposure.

For mutualist pathogen differentiation, L5 day 1 animals were fed on a regular diet sprinkled with 250 µl of 2.7 × 10^9 cells/ml of the isolated *Microbacterium* sp. (#3) or *Enterococcus* sp. (#4) for 4 days. The food was renewed every day.

The detailed experimental procedure is shown in **Fig. 10**. Only animals of the same developmental stage (L5 larvae, day 5–6 if not indicated otherwise) were included in this study. We selected this developmental stage because the animals are very similar in size but also large enough for CT, MR, and PET (6.7 cm in length with a coefficient of variation = 9.3%; **Fig. 10**). Abnormal animals or animals with morphological or behavioral signs of late L5 development (e.g., a noticeable dorsal vessel or cessation of feeding) were excluded.

Unlike vertebrate research animals, working with insects does not require any approval in Europe or the United States.

Magnetic resonance imaging

The Bt-infected larvae were imaged at 12, 36, and 42 h post-feeding, while all other treatments were imaged 12 h post-feeding (**Fig. 10**). For immobilization, larvae were cooled for 30 min on ice and then injected into the dorsal vessel (at the level of the 7th abdominal segment) with 0.1 ml 0.2 M Gd-BOPTA (MultiHance, Bracco Diagnostics, Milan, Italy, #12406641) in 0.9% NaCl or fed with Gd-BOPTA for 12 h. For further immobilization, animals were carefully fixed with Leukosilk S (BSN Medical, Charlotte, NC) on cardboard. The isolated midgut was exposed to 0.2 M Gd-BOPTA in 0.9% NaCl for 5 min, and then washed twice with 0.9% NaCl and deposited in 0.9% NaCl for MRI. Images were taken 15 min after the application of contrast agent (CA) with a standard four-channel flex coil in a clinical Siemens Magnetom Symphony 1.5 T MRI system with a maximum gradient field strength of up to 30 mT/m (Siemens Healthineers, Erlangen, Germany). Axial T1-weighted images were measured using an SE sequence (+FS) with the following settings: RT/TE = 736 ms/15 ms, flip angle = 90°, FOV = 178 × 260 mm, matrix = 512 × 352, and a slice thickness of 3 mm. Coronal T1-weighted SE sequences with FS were acquired with the following parameters: RT/TE = 451 ms/15 ms, flip angle = 90°, FOV = 280 × 280 mm, matrix = 512 × 512, and a slice thickness of 3 mm. Furthermore, axial T2-weighted images were recorded using an SE sequence with the following

settings: RT/TE = 4720 ms/96 ms, flip angle = 180°, FOV = 280 × 512 mm, matrix = 512 × 208, and a slice thickness of 3 mm. Coronal T2 weighted SE images were measured with the following settings: RT/TE 2000 ms/96 ms, flip angle = 180°, FOV = 220 × 220 mm, matrix = 512 × 261, and a slice thickness of 3 mm. The T1-weighted axial sequences were analyzed with Horos v3.3.5. The signal enhancement in T1-weighted sequences was calculated and defined as the normalized T1 signal. The ratio of the maximum T1-weighted signal from the gut wall to the T1-weighted signal from the gut lumen was calculated for every slice. From these values, mean values for each animal were calculated. The maximum gut wall thickness measured in contrast-enhanced T1-weighted MRI was measured manually at the thickest visible spot on each slice. The accuracy of these measurements was verified through a Full Width at Half Maximum (FWHM) analysis (**Fig. S7**+**S8**). Then mean values were calculated as described above. The T2-weighted signal of the gut wall was measured as the maximum T2 signal value on every slice, and mean values per animal were calculated.

Computed tomography

All CT scans except the dexamethasone rescue experiment were performed on a clinical Siemens SOMATOM Emotion 6 (110 kV, 80 mAs and 1 mm collimation) (Siemens Healthineers, Erlangen, Germany). Larvae were prepared as described for the MRI experiments and injected or fed with 0.1 µl of 50% iodixanol (Visipaque 320, GE Healthcare, Solingen, Germany, #1105030) in 0.9% NaCl (**Fig. 10**). Time-dependent differences in the contrast-enhanced gut wall thickness of control and Bt-infected animals were measured at 9, 14, and 19 min. The isolated larval midgut was exposed to 50% iodixanol in 0.9% NaCl for 5 min, then washed twice with 0.9% NaCl and deposited in 0.9% NaCl for CT imaging. After CT, axial sequences were analyzed with Horos v3.3.5. The reconstruction interval was 1.25 mm. The maximal gut wall thickness in contrast-enhanced CT was measured as described for MRI, and the maximal contrast-enhanced CT gut wall signal density was measured at maximum density

on each slice, with mean values calculated for each animal. A subset of animals without gaps in the gut wall thickness measurements along the whole midgut was selected for sequential treatment-specific analysis of the axial CT scans (**Fig. 4d** and **8d**). For better comparability of the thickness measurements, the first 10 measurements were excluded. The resulting treatment-specific sequential gut wall thickness curves (c=12, u=22, u+DPI=22, u+NAC=23, Bt=13, DSS=14, *E.coli*=12) were compared with an extra sum-of-squares F test and tested whether each treatment had the same slope and intercept and could be represented as a single global model (H0) or if individual models for each treatment should be employed (H1).

The dexamethasone rescue experiment was imaged with a SOMATOM Force (Siemens Healthineers, Erlangen, Germany) with the following settings: 110 kV, 102 mAs and 0.6 mm collimation. All other settings were kept as close as possible to the SOMATOM Emotion 6 scans.

Positron emission tomography

We followed the same protocol as described above for MRI (**Fig. 10**). Larvae were fed with Bt, DSS, *E. coli,* or control diets and fasted for 12 h. Cooled and immobilized animals were injected with 0.1 ml 1 MBq/ml 2-[^{18}F]-deoxy-D-glucose (FDG, Life Radiopharma f-con, Holzhausen, Germany, e.g. #220107.1) in 0.9% NaCl in the caudal dorsal vessel and kept cool at 13 °C. FDG PET was performed 3 h after injection in larvae 3, 12, and 24 h after Bt infection with a PEM FLEX Solo II system (CMR Naviscan, Carlsbad, CA) using an in-plane spatial resolution of 1.8 mm and a between-plane resolution of 4–6 mm as previously described.[2] The emission scan time was 15 min. Maximum PUV (PUV_{max}) was measured for each larva in a rectangular region enclosing the apical third of each animal without the head using MIM Viewer (PEM b1.2.4; **Fig. 1O**). To ensure high throughput PET screening, we used 1 kg and 1 MBq as the default option to measure PUV_{max}. The PUV_{max} was normalized for the activity and mean

weight of the larvae as the PUV_{maxn}. We determined the $\bar{x}$ mean weight of the larvae, which were very similar in size, as 7.419 g (**Fig. 10**).

The PUV_{maxn} was calculated using the following formula:

$$PUV_{maxn} = \frac{PUV_{max}}{\frac{activity\ [MBq]}{\bar{x}\ weight\ [Kg]}} \qquad (1)$$

We also isolated the midgut ($\bar{x}$ = 0.81 g), the head ($\bar{x}$ = 0.296 g), as well as fat body tissue and hemolymph (both weighed individually because uniform sampling was not possible), and compared the PUV_{maxn} values 39 min or 3 h after ^{18}F-FDG injection in animals 12 h after Bt infection. The removed tissue was washed in 0.9% NaCl.

Statistics

For statistical analysis we generally used PRISM v8, with only the general linear model calculated using Statistica v12.5.192.7. To evaluate different parameters for CT and MRI diagnostics, all axial slices from a given larvae were measured, and the mean value per animal was calculated. PUV_{maxn} values were log or square root transformed to achieve normal distribution. Depending on the data distribution (evaluated using the Shapiro-Wilk test), we used one-way analysis of variance (ANOVA) and Tukey's multiple comparisons test or the Kruskal-Wallis test and Dunn's multiple comparisons test. When two treatments were compared, we applied a t-test or Mann–Whitney U-test (two-sided). A Pearson product-moment correlation between parameters was calculated to identify correlations between the CT, MRI, and PET findings. Images were inspected and screened for artifacts directly after image acquisition. Larvae were excluded if a large amount of CA spilled over after injection into the dorsal vessel due to animal movement during image acquisition (MRI control = 2, DSS 5% = 2; CT DSS 5% = 1, *E. coli* = 1). Additionally, three outliers were excluded using the ROUT (Q

= 1%) method (MRI control = 1; PET DSS 5% = 2). To test whether small potential differences in larval size affect the epithelial thickness, we used general linear models with MR/CT gut wall thickness or PET PUV_{maxn} as the dependent variable, treatment as a category factor, and animal length as a continuous predictor. ROC curve analysis was carried out using PRISM, including sensitivity and specificity for the listed parameters. The corresponding threshold values were used to compare Bt-infected, DSS or *E. coli*-fed, and control animals using multiple Chi-square tests. The manual thickness measurements were validated via semi-automatic FWHM thickness measurements using OriginPro 2020b and Analyze 14.0. Bland Altman plots have been created using PRISM.

Small animal MRI (µMRI)

The insect larvae were fixed with tape on a standard animal bed and anesthetized with 1–2% isoflurane. Like rodents, the larvae tolerated isoflurane anesthesia very well, and even higher concentrations of up to 4% isoflurane seemed not to harm the animals. For image acquisition, we used a vertical 9.4 Tesla Bruker Wide Bore NMR spectrometer equipped with the actively shielded gradient system Micro 2.5 (1.5 T/m) and a 25-mm ^{1}H quadrature coil (Bruker, Billerica, MA). The dorsal vessel (heart) of the larvae was cannulated, and following the acquisition of baseline images the CA was injected directly into the heart with the same volume and concentration as used for macro-MRI (0.1 ml 0.2 M Gd-BOPTA). Cardiac motion (the contraction of the dorsal vessel) was visualized using a retrospectively gated Intragate-FLASH (fast low-angle shot) cine sequence with the following parameters: (1) TR = 10 ms, TE = 2.31 ms, flip angle = 10 , FOV = 20 × 60 mm^2, matrix size (MS) = 192 × 64, and a slice thickness (ST) of 1.5 mm; (2) TR = 10 ms, TE = 2.31 ms, flip angle = 10, FOV = 20 × 40 mm^2, MS = 128 × 64, ST = 1.5 mm; (3) TR = 10 ms, TE = 2.31 ms, flip angle = 10, FOV = 20 × 20 mm^2, MS = 128 × 64, ST = 1.5 mm. For the acquisition of axial images, a FLASH sequence was used with the following parameters: TR = 50 ms, TE = 3.28 ms, flip angle = 75, FOV = 20 × 20

mm^2, MS = 256 × 256 after zero filling, ST = 1 mm. The first pass of the injected CA was monitored by the retrospectively gated Intragate-FLASH cine sequence (TR = 9.31 ms, TE = 1.77 ms, flip angle = 10, FOV = 18 × 50 mm^2, MS = 96 × 256, ST = 1 mm). For image processing, a heart rate of ~25 bpm was preset, which facilitated accurate data reconstruction.

Ex vivo µCT

For µCT imaging, 10 L5d6 animals were sacrificed and the isolated alimentary tract was incubated in 4% PFA in PBS with in 1% phosphotungstic acid (PTA, Carl Roth, Karlruhe, Germany, #2635.4) for 2-5 days. We acquired µCT images using a Bruker (Billerica, MA) Skyscan 1173 system with an isotropic voxel size of 7.47 µm (50 kVp and 160 µA). Images were reconstructed with NRecon using an 8-bit grayscale range, followed by image analysis with CTAn, DataViewer, and Horos v3.3.5. The details of each scan are given in **Tab. S4**. The whole midgut was analyzed. The µCT maximum gut wall thickness was measured on every 100th slice, and mean values per animal were calculated and compared to the corresponding clinical CT values.

Optoacoustic tomography

For optoacoustic tomography, larvae were handled according to the Arthropod Containment Level 1 guidelines, and work was reviewed and approved by the Memorial Sloan Kettering Cancer Center Institutional Biosafety Committee. The feasibility of optoacoustic tomography in *M. sexta* was demonstrated by the oral application of black India ink (American Mastertech Scientific, Lodi, CA, #STIINPT PINT) and subsequent imaging of the gut. Black India ink was added to the water at a final concentration of 1% before adding to the food mixture. The larvae were incubated for 12 h with the dyed food and then anesthetized with isoflurane (5 min, 5% v/v in 100% O_2) before imaging. Optoacoustic tomography was carried out at 800 nm using a small animal optoacoustic tomography device (MSOT inVision-256 TF, iThera Medical,

Munich, Germany). Once anaesthetized, the (limp) larvae were placed into the holder in a supine position with a bed of ultrasound gel (Aquasonic Clear, Parker Laboratories, Fairfield, NJ, #03-08). Ultrasound gel was then added to cover the rest of the larvae. The holder was sealed, and care was taken to ensure no bubbles were present and the ultrasound gel surrounded the larvae. Transverse optoacoustic slices were acquired at 800 nm with a step size of 0.3 mm, a FOV of 25 mm, and a resolution of 75 µm. Slices were acquired covering the entire larvae and 10 laser pulses were averaged for each slice. Two to three larvae at the 3rd pupal stage could readily fit in the holder at a time. Once imaging was complete, larvae were removed from the holder and wiped to remove excess ultrasound gel. No adverse effects of the imaging, immobilization, or anesthesia were observed. The data were reconstructed offline using back projection methods in ViewMSOT (iThera Medical, Munich, Germany). Representative cross sections were chosen to determine optoacoustic intensity. Pixel values across lines covering the diameter of the larvae were recorded (Fiji, ImageJ v2.1.0). Data were then normalized to each corresponding trace, interpolated and smoothened to generate the graphs (Matlab, 2020a).

Survival

Survival kinetics were subject to Kaplan-Meier survival analysis with a log-rank (Mantel-Cox) test to detect differential survival. Each survival experiment was done in triplicate. The Kaplan-Meier plots show the sum of these experiments. The n refers to the number of animals used for the respective analysis.

Three-dimensional reconstruction

For 3D reconstruction, sagittal T1 weighted (n = 5), axial CT sequences (n = 2), axial µCT (*ex vivo*) sequences, and axial T1 weighted DICOM data were processed and segmented with 3D Slicer v4.8.1 r26813 or Horus v3.3.5. Back projection based optoacoustic reconstructions were exported as 3D tiff stacks from ViewMSOT. The exported stack was then imported to Fiji and

visualized using the 3D Viewer plugin, with spacing set to that of the transverse slice steps (0.3 mm).

Confirmation of MR and CT resolution

In the first set of experiments, we quantified the CT and MR resolution using different glass capillaries (Hilgenberg, Malsfeld, Germany). We used capillaries with 4, 4.5, 7, 8 and 9 mm inner diameters, filled with 0.1 mmol Gd-BOPTA in 0.9% NaCl and capillaries with outer diameters of 4.1, 4.7, 10, and 11 mm filled with 50% iodixanol in 0.9% NaCl (**Fig. S4**). We also used a capillary system with two capillaries stacked one into another. The internal space between these capillaries was filled with 0.1 mmol Gd-BOPTA in 0.9% NaCl. The diameters of the glass capillaries were measured to appraise the empirical resolution of the CT and MR images. (Because the widths of these capillaries were not normally distributed, multiple Mann–Whitney U-tests were performed to estimate the resolution). The mean of the standard deviations of each glass capillary was calculated and then doubled to obtain the empirical CT and MRI resolution for cylindrical or tube-shaped objects.

In addition, we measured an empty glass capillary with a known wall thickness of 1 mm (Hilgenberg, Malsfeld, Germany) in CT with the same imaging settings used for caterpillar imaging. The wall thickness measurements were calculated as FWHM measurements (**Fig. S5**). Furthermore, to assess the capability of currently available CT scanners for long cylindrical structures, we used a 3D printed micro PET hot rod phantom (Derenzo phantom, according to PTW specitications, **Fig. S6**). 3D printing was done using a Anycubic Photon Mono X 3D printer (Anycubic, Shenzhen, China) with ABS-like creamy white 3D printer resin (Phrozen Technology, Taiwan). The Derenzo phantom was created with six differently sized segments of cylindrical holes, ranging from 0.6 0.8, 1.0, 1.2, 1.5 to 2 mm (**Fig. S6**). After the printing procedure, the object was washed twice with isopropyl alcohol and then hardened under UV light according to the manufacturer's instructions.

The phantom was initially imaged using our µCT Skyscan 1173 system (Bruker, Billerica, MA). Next, it was imaged with the Photon-counting CT: NAEOTOM Alpha (Siemens Healthineers, Erlangen, Germany), the Dual-source CT SOMATOM Force (Siemens Healthineers, Erlangen, Germany), the signal source CT SOMATOM X.ceed (Siemens Healthineers, Erlangen, Germany) and SOMATOM go.Top (Siemens Healthineers, Erlangen, Germany) (**Fig.S6**). The bore thickness was measured semiautomatically using the FWHM measurements. The double mean SD of the measurements was used as a proxy for spatial resolution of long cylindrical objects in the mentioned CT scanners.

Verification of Gibbs artifacts

To determine whether the hypointense and hyperintense rings and lines in the larval alimentary tract were Gibbs artifacts, we used water-filled phantoms and took T1 and T2 weighted images with matrices of different sizes. We used a capillary filled with water and two stacked capillaries with the interspace filled with water as phantoms (**Fig. S12**).

Optical density measurements of the hemolymph and gut lesions

L5d2 larvae were fed with Bt (n = 12) or the control diet (n = 12) and were cooled on ice 36 h later. The surface of the insects was sterilized with 70% ethanol, and half of the dorsal horn was removed. The hemolymph was collected individually in sterile cups, and 10 µl was incubated in standard nutrient broth I (Carl Roth, Karlruhe, Germany, #AE92.1) overnight before measuring the optical density at 560 nm (OD_{560}). L5d6 larvae 24 h after Bt infection (Bt n = 3 and control n = 6) were dissected and the midguts were examined.

Differential hemocyte count

The relative number of hemocytes from Bt-treated and untreated (control) larvae were compared. Larval hemocytes were collected, washed, and immunolabeled with the plasmatocyte specific marker MS#13 (plasmatocytes-specific β-integrin),[3,4] the granular cell specific monoclonal antibody MS#7,[3] the oenocytoid labeling *M. sexta*-specific β(1,3)-glucan recognition protein 1 immune serum,[5] (1:500) or the spherule-labeling anti-*Ephestia kuehniella* hemolymph esterase immune serum[6] (1:1000) as described elsewhere.[7] The donkey anti-mouse IgG H&L (DyLight 549, ab96876) and goat anti-rabbit IgG H&L (DyLight® 488, ab96899) were used as secondary antibodies. Images were taken on an Olympus BX60 microscope using an XC10 camera (Olympus Life Sciences, Tokyo, Japan). The relative number of each hemocyte type (plasmatocytes, granular cells, spherule cells, and oenocytoids) was determined in n = 4 animals per treatment using ImageJ v1.46 and the plugin Cell Counter (http://imagej.nih.gov/ij).

Cryosectioning and immunohistochemistry

The medial midgut of L5d2 larvae fed with Bt (n = 7) or the control diet (n = 7) was fixed for 1 h in 3.7% (w/v) paraformaldehyde (PFA) in MS-saline. The tissue was embedded for 30 min in OCT compound (Sakura Finetek, Torrance, CA, #4583) and then frozen using a frigocut cryotome (Cryo Leica CM1950, Leica Biosystems, Wetzlar, Germany). Sections were collected at −26 °C using microscope slides coated with 2% silane in acetone. The freshly-loaded microscope slides were dried for 10 min at room temperature for better tissue adherence. For immunofluorescence staining, the cryosections were incubated for 1 h in 3% (w/v) BSA and 5% (w/v) goat serum in Tris-buffered saline (TBS). The samples were then incubated with the primary monoclonal antibody (plasmatocytes-specific marker Ms#13) and the appropriate secondary antibody (Dylight 549, Abcam, Cambridge, United Kingdom) diluted 1:2500, as previously described.[8] Additionally, the samples were incubated in 5 μM FITC-phalloidin

(Sigma-Aldrich, St. Louis, MO, #P5282) in 0.5% BSA in TBS for 1 h, washed three times for 5 min with TBS, and stained with DAPI (Sigma-Aldrich, St. Louis, MO, #D9542). Finally, the sections were rinsed three times for 5 min with TBS, covered with Fluoromount-G (SouthernBiotech, Birmingham, AL, #0100-01), and viewed under a BX60 Olympus fluorescence microscope.

Paraffin-embedded sections and histochemical staining

Larvae were anesthetized on ice and 1 ml of 10% buffered formalin was injected into the midgut. The dorsal horn was removed, and the animal was placed in a Falcon tube containing 10% buffered formalin for 40 min. Abdominal segments 2–4 were then removed and incubated in 10% formalin overnight. Each sample was then placed in a plastic cassette and dehydrated in an ascending ethanol series at 37 °C, followed by 100% isopropanol and three changes of xylene before infiltration with Roti-Plast paraffin (Carl Roth, Karlsruhe, Germany, #6642.5) in a Tissue-Tek VIP machine (Sakura Finetek, Torrance, CA). The sample was then embedded in Tissue-Tek TEC paraffin (Sakura Finetek, Torrance, CA, #4005) and sectioned on a rotary microtome (Leica RM2255). The sections were mounted on Superfrost Plus Slides (Langenbrinck, Emmendingen, Germany, #03-0060) and stained with hematoxylin and eosin using an automated robot-stainer (HMS740 Microm). Finally, the samples were covered with Tissue-Tek Coverslipping Film (Sakura Finetek, Torrance, CA, #4770) using an automated coverslipper (Vogel, Kevelaer, Germany).

Scanning electron microscopy

Midguts from L5d2 larvae fed with Bt (n = 3) or the control diet (n = 5) were dissected in PBS and fixed in 2% PFA plus 0.5% glutaraldehyde in PBS for 3 h at room temperature. The fixative was diluted 1:10 and the samples were stored at 4 °C. Next, the midgut samples were rinsed in PBS and ultrapure water and then dehydrated in an ascending ethanol series on ice (30%, 50%,

70%, 80%, 90%, 96%, 99.8%, and 100%) for 10 min at each concentration. The samples were critical point dried (Balzers CPD 030, Balzers, Liechtenstein), sputter-coated with gold (Balzers SCD 004, Balzers, Liechtenstein), and then mounted on aluminum stubs using Ag paste (Electrodag 1415, Plano, Wetzlar, Germany, #16062). The samples were analyzed using a Zeiss EM9DSM982 scanning electron microscope (Zeiss, Oberkochen, Germany).

Semi-quantitative RT-PCR analysis

Midgut samples were taken from control and Bt-infected animals 24 h after MRI, and RNA was isolated using the RNeasy Mini Kit according to the manufacturer's instructions (Qiagen, Hilden, Germany, #74004). DNA contamination was removed by incubating samples with 1 U/µl TURBO DNase (Invitrogen, Waltham, MA, #AM2238) for 30 min at 37 °C, then inactivating the enzyme by incubation in 5 mM EDTA at 75 °C for 10 min. The absence of DNA contamination was confirmed by PCR (Mastercycler Gradient, Eppendorf Scientific, Hamburg, Germany) for every sample (95 °C for 2 min followed by 34 cycles of 95 °C for 30 s, 57 °C for 1 min, and 72 °C for 30 s, followed by a final elongation step at 72 °C for 10 min). Each 25-µl reaction contained 250 ng RNA, 1 U MyTaq DNA polymerase (Bioline, London, UK, #BIO-21105), and 5 pmol of the forward and reward EF1(α) primers. Reverse transcription was carried out with the Transcriptor High Fidelity cDNA Synthesis Kit (Roche Pharmaceuticals, Basel, Switzerland, #5081955001) in a 20-µl reaction with 500 ng RNA, according to the manufacturer's instructions. The cDNA was amplified by heating to 95 °C for 2 min, followed by 38 cycles of 95 °C for 30 s, 56 °C for 45 s, and 72 °C for 30 s, followed by a final elongation step at 72 °C for 10 min. The primers are listed in Table S1. Control reactions were carried out using primers specific for EF1α as a housekeeping gene with an annealing temperature of 75 °C for 30 s. See the source data file for a presentation of complete, uncropped gels.

Quantitative real-time RT-PCR analysis

Total RNA was prepared from different gut regions (foregut, anterior-, median-, posterior-midgut and ileum) of 6 control or 6 animals exposed to uracil (8 h) and from pools of tissues (head, fat, central nervous system, labial gland, muscle, skin, trachea and Malpighian tubules) from *M. sexta* fifth instar larvae (L5d5) using the RNeasy Mini Kit (Qiagen, #74004) followed by DNase I digestion (Thermo Scientific, Waltham, USA, #EN0521). cDNA synthesis was performed using the biotechrabbit cDNA Synthesis Kit and oligo(dT) primers following the manufacturers' instructions (biotechrabbit GmbH, Berlin, Germany, #BR0400401). The qPCRs were performed in triplicates with the qTOWER3 (analytikjena, Jena, Germany) using qPCR SyGreen Mix Fluorescein (Nippon Genetics, Tokyo, Japan), 100 ng of the respective cDNA and pairs of MsDUOX specific primers (forward primer: 5'-AAGCACTTCGAGTGGTTCATC-3'; reverse primer: 5'-TCAAGAAGGAGGACATGTCG-3', Table S1). Relative gene expression was calculated based on the comparison of CT values for MsDUOX and the reference gene MsEF1α (forward primer: 5'-CTTCACAGCTCAGGTCATCG-3'; reverse primer: 5'-GAAGGACTCCACACACATGG-3', Table S1). The specificity of the PCR was confirmed by melting-curve analysis and mean normalized expression was determined according to [9]. Mean-normalized MsDUOX expression values of the anterior digestive tract (FG, Foregut; aMD, anterior midgut; and MD, medial midgut) or the posterior digestive tract (pMD, posterior midgut and Il, ileum , part of the hindgut) were reported.

Polyclonal anti-DUOX antibody

The polyclonal rabbit anti-DUOX antibody was custom-created by Davids Biotechnologie (**Fig. S26**). One rabbit was injected with the DUOX peptide LLRDKHCRYGKAPGGHDAIR

(amino acids 342–361, within the extracellular PHD). The affinity-purified antibody was used at a dilution of 1:100 (v/v) for Western blot and immunohistochemistry.

Western blot

L5d5 larvae fed on the control diet or the diet supplemented with 0.1 M uracil were killed after 24 h and sections of the foregut, anterior, medial and posterior midgut, hindgut, and other tissues were washed with ice-cold PBS and immersed in Tris-glycine electrophoresis buffer with proteinase inhibitor cocktail P2714 (Sigma-Aldrich, St. Louis, MO, #P2714-1BTL) according to the sample weight (1:3; w/w). The tissue was shredded with a pestle and sonicated in ice water before adding 5 × SDS gel loading buffer[10] (1:5; v/v) and heating for 5 min at 96 °C. Samples were then stored at –20 °C.

Sodium dodecylsulfate polyacrylamide electrophoresis (SDS-PAGE) was carried out as previously described.[10,11] Briefly, we prepared polyacrylamide gels with a 5% stacking gel and a 7.5% separation gel (w/v) and loaded 30 µl of sample per lane. The samples were separated at a voltage of 100 V with size markers for comparison (Color Plus Prestained Protein Ladder, Broad Range, 10–230 kDa, (New England Biolabs, Ipswich, MA). The separated proteins were electroblotted for 1 h at a constant 1 mA/cm^2 onto PVDF membranes or the gel was stained with Coomassie R-250 (Thermo Fisher Scientific, Waltham, MA, #20278). After protein transfer, the PVDF membranes were blocked with 5% (w/v) non-fat dried milk in TBS (pH 7.5) for 1 h then incubated with the polyclonal rabbit anti-DUOX primary antibody diluted 1:100 (v/v) in 5% non-fat dried milk in TBS (pH 7.5) and 0.1% (v/v) Tween-20 at 4 °C overnight on a tilting platform. The membranes were rinsed three times with 0.1% Tween (v/v) in TBS and stained with the anti-rabbit alkaline phosphatase (ALP)-conjugated secondary antibody (goat anti-rabbit IgG H&L, Roth #4751) diluted 1:5000 (v/v) for 2 h in 2% non-fat dried milk in TBS (pH 7.5) and 0.1% Tween-20. The membranes were then washed four times with TBS (pH 7.5) and 0.1% Tween-20 for 10 min. The protein bands were visualized by adding

bromochloroindoyl phosphate (BCIP) and nitroblue tetrazolium (NBT) as previously described.[12] The ALP reaction was stopped after 10 min and the DUOX signal was assessed as present or absent. Then, the membranes were hydrated in 100% methanol, washed with TBS+T, and rinsed with ultrapure water. Next, the membranes were stained with Ponceau S staining solution (Cell Signaling, Danvers, MA, #59803S) and a protein with a rel. molecular weight of 30 kDa was used as a loading control. Finally, the number of DUOX-positive gut samples of control and uracil-exposed animals were compared (**Fig. 7B**+**C** and **S22**). The Coomassie-stained gels were dehydrated on a vacuum gel dryer. See the source data file for a presentation of complete, uncropped gels.

Detection of HOCl with R19S

HOCl was imaged as previously described.[13] Briefly, larvae fasted 3 h before the treatment were fed on the control diet or with a 0.1 M uracil suspension with or without DPI (65 μM) and NAC (72 μM) for 1 h. The larvae were then exposed to the same diets supplemented with 42 μM R19S (FutureChem, Seoul, South Korea, #FC-8001-0010) for 2 h. After this treatment, the gut was dissected, washed in PBS, and fixed in 4% PFA in PBS for 10 min, before washing in PBS and mounting on glass slides. Images were collected with a Leica SP8 confocal laser scanning microscope (CLSM) with an excitation wavelength of 514 nm and an emission wavelength of 530–603 nm.

Manduca sexta DUOX tertiary structure prediction

Homology modeling was carried out in Raptor X[14-16] using lactoperoxidase (2E9E, *Capra hircus*) and DUOX1 (6WXV, *Mus musculus*)[17] as templates for the PHD and human DUOX1 (7D3E, *Homo sapiens*)[18] as a template for the whole protein. We used template-based protein structure modeling[14,15,19-22] and model-assisted protein binding site prediction[14,15,21,23] as well

as a structure alignment calculation.[24,25] The structures were visualized in UCSF Chimera v1.11.2. General domain prediction, unless otherwise stated, was carried out in SMART, whereas transmembrane domain prediction was achieved using phobius. Signal peptides were predicted using SignalP 4.0.

CFU count after uracil treatment

L5d6 larvae were raised on a normal diet (n = 15) or on a diet containing 0.1 M uracil (n = 17) for 20.5 h. They were then transferred to a sterile Petri dish in a laminar flow cabinet for fecal collection. One fresh feces pellet per animal was diluted 1:10,000 in standard nutrient broth I and transferred to agar plates based on the same medium (pH 7 and pH 9). The agar plates were incubated at 37 °C for 96 h at pH 7 or for 8 days at pH 9. CFUs were counted with OpenCFU-2.5[26].

Identification of cultivable bacteria from fecal pellets

All 64 (pH 7 and pH 9) agar plates were scrutinized under a stereomicroscope, and cell morphology was evaluated under a phase-contrast microscope. Colonies were classified and isolated based on colony morphology, cell morphology, and differential growth on selective media (see below). Five different colony archetypes were identified and purified. DNA extraction and 16S ribosomal DNA (rDNA) amplification was performed as previously described[27] using the G7 primer mix or as previously described[28] with the listed primers. PCR products were sequenced (Microsynth SeqLab, Göttingen, Germany), and used as a BLAST search query (https://blast.ncbi.nlm.nih.gov/Blast.cgi). A match was accepted at ≥ 98% sequence identity (see **Table S11**). Rarefaction curves calculated with EstimateS v9[29] revealed a sufficient sampling of the low-diversity bacterial community in *M. sexta* fecal pellets (**Fig. 9H**).

Characterization of host-mutualist and host-pathogen interactions

To further characterize the fecal bacteria of *M. sexta*, several selective and differential media were inoculated with each isolate (see **Table S5**). To determine the pathogenicity of these bacteria, L5d5 larvae were fed on a diet spiked with 50 µl of each bacterial suspension (2×10^8 cells/ml in standard nutrient broth I, Carl Roth, Karlruhe, Germany, #AE92.1). Each diet group (different bacterial species and control) comprised 24 individual larvae. The diet was renewed daily, and survival was recorded. Antimicrobial susceptibility testing (AST) was performed to determine if the isolated bacterial species produce bactericidal toxins (e.g., bacteriocins). We inoculated 30 ml of Oxoid agar No. 1 (Thermo Scientific, Waltham, USA, #10055782) (47 °C) with 100 µl of each overnight culture and poured the agar into sterile Petri dishes. We punched 4 mm holes into the cooled agar. Overnight cultures of the isolated bacteria were centrifuged twice at 5000 g for 20 min, and 4 µl of the supernatant was added to the holes. The agar plates were incubated for 48 h at 37 °C before we measured the zone of inhibition. The susceptibility of the bacteria against different antibiotics, hemolymph, and different concentrations of HOCl was evaluated using the same method. Linear regression (test for equal elevations) was used to determine differential sensitivity of *Enterococcus casseliflavus*/*gallinarum* #1 and *Enterococcus* sp. #4 against HOCl. HOCl was prepared as described previously[13] (NaClO, Carl Roth, Karlruhe, Germany, #9062.1). Both enterococci inhibited the pathogenic *Microbacterium* sp., and this was confirmed by spectrophotometry, by measuring the optical density at 415 nm (OD_{415}). The supernatant of both enterococci was diluted (1:1, 1:10, 1:100, and 1:1000) with the overnight cultures in 96-well plates and incubated at room temperature on a platform shaker. The OD_{415} was measured after 7 h. To assess whether the inhibitory effect of the enterococci was biologically relevant, we compared the survival of animals fed on (1) a diet inoculated with the pathogenic *Microbacterium* (2×10^8 cell/ml, n = 40) to animals fed with diet inoculated with this strain and supernatants from the two enterococci (1:1 with 2×10^8 cell/ml, each n = 24) or control animals (n = 41). To evaluate the potential pleiotropic effect of bacterial

maintenance, freshly hatched larvae were fed with penicillin (Sigma-Aldrich, St. Louis, MO, #PENNA, n = 47, control n = 38), which was bactericidal to our isolates at 500 µg/ml in the AST. Accordingly, 50 µl penicillin was spread on the regular diet, and the animals were weighed after 16 days of treatment. The clearance of gut bacteria by penicillin treatment was confirmed by measuring the OD_{415} of fecal pellets diluted 1:10,000 after 12 h, as discussed above.

Table S1: Sequences of RT-PCR primers (5′ to 3′ direction).

Gene	GenBank accession number	Primer	Sequence	Product size (bp)
MsEF1 (α)	AF234571.1	Forward<br>Reverse	5’-CTTCACAGCTCAGGTCATCG-3’<br>5’GAAGGACTCCACACACATGG-3’	229bp
MsAttacin 1	DQ072728.1	Forward<br>Reverse	5’-CCTGTCGTGCCTCTTCCTC-3’<br>5’GAGCGAGGTGGTCTTGTC-3’	751bp
MsGloverin	AM293324.1	Forward<br>Reverse	5’-GAAGGTCTTCGGAACTCTGG-3’<br>5’CTGGAAGAGACCTTGGAAGC-3’	352 bp
MsDUOX	MK983103.1	Forward<br>Reverse	5’-AAGCACTTCGAGTGGTTCATC-3’<br>5’-TCAAGAAGGAGGACATGTCG-3’	228 bp

Table S2: Measurements of gut wall thickness (after Bt, DSS and *E.coli* treatments) and influence of animal length.

Mean differences between CT gut wall thicknesses of different treatments.

Test details	Mean 1 [mm]	Mean 2 [mm]	Mean Diff [mm]	SE of diff.
control vs. Bt	0.8771	1.338	**-0.461**	0.05558
control vs. DSS 5%	0.8771	1.158	**-0.2813**	0.06407
control vs. *E. coli*	0.8771	0.9731	**-0.09597**	0.05994
E. coli vs. DSS 5%	0.9731	1.158	**-0.1853**	0.06407
E coli vs. Bt	0.9731	1.338	**-0.3651**	0.05558
Bt vs. DSS 5%	1.338	1.158	**0.1797**	0.06002

Mean differences between MRI gut wall thicknesses of different treatments.

Test details	Mean 1 [mm]	Mean 2 [mm]	Mean Diff [mm]	SE of diff.
control vs. Bt	1.557	2.198	**-0.6403**	0.08642
control vs. DSS 5%	1.557	2.181	**-0.6238**	0.1269
control vs. *E. coli*	1.557	1.762	**-0.2047**	0.1016
E. coli vs. DSS 5%	1.762	2.181	**-0.4191**	0.1436
E coli vs. Bt	1.762	2.198	**-0.4356**	0.1095
Bt vs. DSS 5%	2.198	2.181	0.01652	0.1333

Generalized linear models, which imply that CT (n = 75) and MR (n = 92) contrast-enhanced gut wall thickness, as well as PUV_{maxn} (n = 88), are treatment-dependent but not dependent on larval size (length).

CT (contrast-enhanced gut wall thickness)	SS	Degrees of freedom	MS	F	p
length	0.10357	1	0.103577	3.30786	0.073227
treatment	2.248926	3	0.749642	23.94076	**<0.000001**
error	2.191867	70	0.031312		

MRI (contrast-enhanced gut wall thickness)	SS	Degrees of freedom	MS	F	p
length	0.30208	1	0.302076	2.58817	0.111288
treatment	7.7577	3	2.585900	22.15584	**<0.000001**
error	10.15413	87	0.116714		

PET (PUV_{maxn})	SS	Degrees of freedom	MS	F	p
length	0.53368	1	0.533679	2.120157	0.149144
treatment	6,55584	3	2.185281	8.681500	**0.000045**
error	20.89250	83	0.251717		

Table S3: Diagnostic findings after Bt, DSS 5% and *E. coli* treatment with corresponding threshold values.

Diagnostic finding CT, MR or PET	Change after **Bt** treatment (compared to control)		Change after **DSS 5%** treatment (compared to control)		Change after ***E. coli*** treatment (compared to control)		**Diagnostic values**				
	without threshold (1 way-ANOVA or Kruskal-Wallis ANOVA)	above or under threshold? (Chi-squared test)	without threshold (1 way-ANOVA or Kruskal-Wallis ANOVA)	above or under threshold? (Chi-squared test)	without threshold (1 way-ANOVA or Kruskal-Wallis ANOVA)	above or under threshold? (Chi-squared test)	Threshold value	Sensitivity	Specificity	**ROC Area**	*ROC Area from the Literature of human Crohn's disease*
**CT gut wall thickness** (post-iodixanol)	↑**** <0.0001	↑**** <0.0001	↑*** 0.0005	↑** 0.0022	ns	ns	>1.006 mm	96.00%	88.89%	**0.97**	*CT: 0.90 - 0.89 Lee et al. 2009* [30]
**CT signal density** (post-iodixanol)	↑* 0.0132	↑* 0.0254	ns	↑** 0.0012	ns	↑** 0.0076	> 84.23 Hu	84.62%	58.33%	**0.80**	
**MR gut wall thickness** (post-gadolinium)	↑**** <0.0001	↑**** <0.0001	↑**** <0.0001	↑**** <0.0001	ns	ns	>1.833 mm	80.77%	85.37%	**0.86**	*MRI: 0.93 - 0.95 Lee et al. 2009* [30]
**MR normalized T1 signal** (post-gadolinium)	↑**** <0.0001	↑**** <0.0001	ns	ns	ns	ns	>1.883	90%	80.47%	**0.91**	
**MR T2 signal**	↑* 0.0221	↑** 0.0010	ns	ns	/	/	>997.6	85%	68.75%	**0.78**	
**FDG-PET PUV_{maxn}**	↓*** 0.0010	↓**** <0.0001	↓** 0.0054	↓* 0.047	ns	↑* 0.0282	<0.5405 kg/MBq	84%	65.52%	**0.72**	*PET/CT: 0.85 Louis et al. 2007* [31]

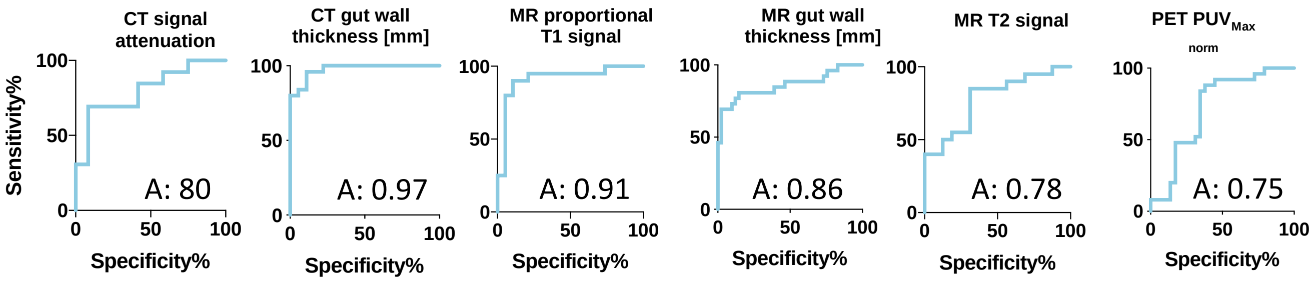

Table S4: Detailed information for each scan and the corresponding scanning parameters.

#	Animal	Scan modality	Contrast agent	Connected scans	Source voltage [kV]	Source current [µA]	Image pixel size [µm]	Filter [mm]	Rotation step [˚]	Frame averaging	Scan duration [h:m:s]
1.1	L5d6 part 1	hydrated PTA scan	PTA	3	60	160	7.47	0.5 Al	0.25	4	01:44:34
1.2	L5d6 part 2	hydrated PTA scan	PTA	5	50	160	7.47	0.5 Al	0.25	4	02:33:15
2	L5d6	hydrated PTA scan	PTA	7	50	160	7.47	0.5 Al	0.25	4	01:44:00
3	L5d6	hydrated PTA scan	PTA	7	50	160	7.47	0.5 Al	0.25	4	01:45:04
4	L5d6	hydrated PTA scan	PTA	5	50	160	7.47	0.5 Al	0.25	4	01:57:32
5	L5d6	hydrated PTA scan	PTA	7	50	160	7.47	0.5 Al	0.25	4	02:45:01
6	L5d6	hydrated PTA scan	PTA	6	50	160	7.47	0.5 Al	0.25	4	03:42:03
7	L5d6	hydrated PTA scan	PTA	6	50	160	7.47	0.5 Al	0.25	4	02:03:53
8	L5d6	hydrated PTA scan	PTA	6	50	160	7.47	0.5 Al	0.25	4	01:58:00
9	L5d6	hydrated PTA scan	PTA	6	50	160	7.47	0.5 Al	0.25	4	01:54:22
10	L5d6	hydrated PTA scan	PTA	6	50	160	7.47	0.5 Al	0.25	4	01:45:31

Table S5: Summary of the binding site prediction results for the *Manduca sexta* DUOX PHD using the MsDUOX 3D model ($P = 1.97 \times 10^{-19}$, uGDT(GDT) = 751.2(50.1), uSeqld(SeqlD) = 484(32).

Pocket	Multiplicity	Ligand	Binding residues
1	166	NAG	A97 R99 A301 H489 D552 H555
2	165	HEM	A104 G107 Q108 T111 V115 M116 A117 S118 R241 Q244 N245 A326 A329 F330 R331 G333 H334 V337 W366 L405 L416 L419 R423
3	118	NAG	S195 Q197 S202 S203 L209
4	81	IOD	T57 R58 K59 T60 T322 H323 E324
4	81	NO_3	T57 R58 K59 T60 T322 H323 E324
5	80	Ca^{2+}	E113 T174 W176 S180
6	80	NAG	R314 E316 V317
7	79	NAG	H224 Y225 M228 N370
8	65	IOD	D345 K346 H347 M383 I544 G545
9	54	IOD	N292 L295 Y296 F513
10	53	IOD	T81 K84 I85 D132

NAG = N-acetyl-β-D-glucosamine; HEM = heme or protoporphyrin IX containing Fe; IOD = iodine; NO_3 = Nitrate ion; Ca^{2+} = calcium ion

Table S6: Summary of the binding site prediction results for the *Homo sapiens* DUOX1 PHD using the HsDUOX1 3D model ($P = 1.61 \times 10^{-20}$, uGDT(GDT) = 880(57), uSeqld(SeqlD) = 996(64).

Pocket	Multiplicity	Ligand	Binding residues
1	167	NAG	A93 R95 A298 Q301 V558 H560
2	164	HEM	V100 G103 Y104 L107 V111 S112 V113 E114 R238 R241 E242 V323 S326 E327 Q328 L330 S331 V334 Y367 W408 H419 S422 R426
3	114	NAG	S191 S193 A198 S199 R207
4	98	NAG	T223 N226 E371 P373
5	79	Ca^{2+}	D109 T170 W172 S176
6	79	IOD	Q52 R53 L54 V55 S319 S320 E321
6	79	NO_3	Q52 R53 L54 V55 S319 S320 E321
7	76	NAG	R311 F313 L314
8	64	IOD	N342 A343 S344 L386 I547 D548
9	54	IOD	N289 V292 Y293 R533
10	53	IOD	D77 N80 T81 P128

NAG = N-acetyl-β-D-glucosamine; HEM = heme or protoporphyrin IX containing Fe; IOD = iodine; NO_3 = Nitrate ion; Ca^{2+} = calcium ion

Table S7: Summary of the binding site prediction results for the *Homo sapiens* DUOX2 PHD using the HsDUOX2 3D model (P = 8.26×10^{-21}, uGDT(GDT) = 866(56), uSeqld(SeqlD) = 881(57)

Pocket	Multiplicity	Ligand	Binding residues
1	167	NAG	H99 R101 S304 Q307 V561 H563
2	164	HEM	V106 G109 Y110 L113 V117 S118 V119 E120 R244 R247 E248 V329 S332 E333 Q334 F336 S337 V340 Y373 W414 Y425 S428 R432
3	117	NAG	S197 S199 A204 S205 P207 R213
4	89	NAG	T229 N232 E377 P379
5	79	Ca^{2+}	D115 T176 W178
6	79	IOD	Q58 R59 R60 V61 S325 P326 E327
6	79	NO_3	Q58 R59 R60 V61 S325 P326 E327
7	77	NAG	R317 F319 L320
8	64	IOD	N348 A349 S350 L392 I550 D551
9	54	IOD	V298 Y299 F519 R536
10	53	IOD	R83 N86 A87 P134

NAG = N-acetyl-β-D-glucosamine; HEM = heme or protoporphyrin IX containing Fe; IOD = iodine; NO_3 = Nitrate ion; Ca^{2+} = calcium ion

Table S8: Summary of the binding site prediction results for the *Drosophila melanogaster* DUOX PHD using the DmDUOX 3D model ($P = 1.82 \times 10^{-19}$, uGDT(GDT) = 748(51), uSeqId(SeqId) = 474(32).

Pocket	Multiplicity	Ligand	Binding residues
1	163	HEM	A139 G142 Q143 A146 V150 M151 A152 R278 Q281 N282 Q363 A366 F367 R368 G370 H371 I374 W403 V438 F443 L453 L456 R460
2	152	NAG	F132 N133 R134 A338 D589 M592
3	117	NAG	S230 H232 L237 T238 K240
4	89	NAG	V262 S407 F409
5	79	Ca^{2+}	E148 T209 W211 S215
6	79	IOD	V92 R93 K94 A95 H360 I361 A472
6	79	NO_3	V92 R93 K94 A95 H360 I361 A472
7	76	NAG	K351 D353 I354
8	64	IOD	D382 G383 M420 V581 K582 E585
9	54	IOD	N329 V332 Y333 F550
10	53	IOD	R116 R119 L120 E167

NAG = N-acetyl-β-D-glucosamine; HEM = heme or protoporphyrin IX containing Fe; IOD = iodine; NO_3 = Nitrate ion; Ca^{2+} = calcium ion

Table S9: Diagnostic findings after uracil, uracil + DPI, and uracil + NAC treatment with corresponding threshold values, ROC curve, and statistical tests.

Diagnostic finding MRI or PET	Change after **uracil** treatment (compared to control)		Change after **uracil + DPI** treatment (compared to uracil treatment)		Change after **uracil + NAC** treatment (compared to uracil treatment)	
	without threshold (1 way-ANOVA or Kruskal-Wallis ANOVA)	above or under threshold? (chi-squared test)	without threshold (1 way-ANOVA or Kruskal-Wallis ANOVA)	above or under threshold? (chi-squared test)	without threshold (1 way-ANOVA or Kruskal-Wallis ANOVA)	above or under threshold? (chi-squared test)
**CT gut wall thickness** (post-iodixanol)	↑**** <0.0001	↑**** <0.0001	↓**** <0.0001	↓**** <0.0001	↓**** <0.0001	↓**** <0.0001
**CT signal density** (post-iodixanol)	↑**** <0.0001	↑**** <0.0001	↓**** <0.0001	↓** <0.0028	↓**** <0.0001	↓*** <0.0006
**MR gut wall thickness** (post-gadolinium)	↑**** <0.0001	↑*** <0.0003	↑* <0.0285	ns	↓**** <0.0001	↓*** <0.0003
**MR normalized T1 signal** (post-gadolinium)	↑** 0.0069	↑* <0.0318	ns	ns	ns	↓** <0.0068
**FDG-PET PUV$_{maxn}$**	↑** 0.0049	ns	↓**** <0.0001	↓** 0.001	↓* 0.0283	ns

PET PUV $_{maxn}$

Diagnostic finding	Threshold value	Sensitivity	Specificity	**ROC Area**	*ROC area from the literature of human Crohn's disease*
**FDG-PET PUV$_{maxn}$**	>0.7630 kg/MBq	63.16%	55.17%	**0.68**	*PET/CT: 0.85 Louis et al. 2007*

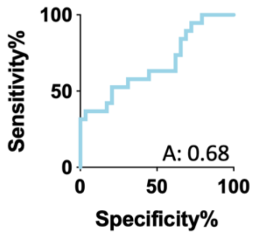

Table S10: Gut wall thicknesses after uracil, uracil + DPI, and uracil + NAC treatments.

Note that only significant differences in gut wall thickness (greater than the doubled mean empirical standard deviation for thickness discrimination [values in bold] for MRI and CT) are discussed in this work.

Mean differences between CT gut wall thicknesses of different treatments.

Test details	Mean 1 [mm]	Mean 2 [mm]	Mean Diff [mm]	SE of diff.
control vs. uracil	0.8771	1.135	**-0.2581**	0.04328
control vs. uracil + DPI	0.8771	0.8572	0.01992	0.04328
control vs. uracil + NAC	0.8771	0.83	0.0471	0.04285
uracil vs. uracil + DPI	1.135	0.8572	**0.278**	0.04106
uracil vs. uracil + NAC	1.135	0.83	**0.3052**	0.04061
uracil + DPI vs. uracil + NAC	0.8572	0.83	0.02718	0.04061

Mean differences between MRI gut wall thicknesses of different treatments.

Test details	Mean 1 [mm]	Mean 2 [mm]	Mean Diff [mm]	SE of diff.
control vs. uracil	1.557	2.062	**-0.5051**	0.07569
control vs. uracil + DPI	1.557	1.831	**-0.2737**	0.06878
control vs. uracil + NAC	1.557	1.657	0.09985	0.07702
uracil vs. uracil + DPI	2.062	1.831	**0.2314**	0.08188
uracil vs. uracil + NAC	2.062	1.657	**0.4053**	0.08891
uracil + DPI vs. uracil + NAC	1.831	1.657	**0.1738**	0.08311

Table S11: Cultivable bacteria isolated from feces of *Manduca sexta*, with further information.

**Sequence number**	**GenBank accession number**	**Sequence length**	**Closest relative(s) (% Sequence identity)**	**Isolate(s) origin**	**Order (phylum or class)**	**Comment** (If already known from *Manduca sexta* gut)
#1.	OP630947	790 bp	-*Enterococcus casseliflavus (100%)*<br><br>-*Enterococcus gallinarum* (100%)	Streptococcus faecium var. Casseliflavus, now known as *Enterococcus casseliflavus* (Naser et al 2006) was isolated **175 insects** Martin and Mundt (1972)<br>known from ***Manduca sexta*** Brinkmann et al (2008)<br>clinical specimens Reid et al. (2001)<br><br>Known from ***Manduca sexta*** Brinkmann et al (2008)<br>***Drosophila*** Cox and Gilmore (2007),<br>**house flies and cockroaches** Ahmad et al. (2011),<br>clinical specimens Reid et al. (2001)	Lactobacillales	Both known from ***Manduca sexta***: Brinkmann et al, 2008, Enterococci were isolated from **213 insects** Mart and Mundt (1972), Enterococci were found in **85 of lar** **Lepidoptera** (Martin and Mundt (1972), referred as <u>*En*</u> <u>*#1*</u>
#2.	OP630948	481 bp	-*Corynebacterium nuruki (100%)*	Alcohol fermentation starter Shin et al. (2011)	Actinomycetales	Genus already known from ***Manduca sexta***: van der Hoeven et al 2008
#3.	OP630949	275 bp	-*Microbacterium paraoxydans* (99%)<br>-*Microbacterium phyllosphaerae* (99%)<br>-*Microbacterium oxydans* (99%)<br>-*Microbacterium testaceum* (99%)<br>-*Microbacterium maritypicum* (99%)<br>-*Microbacterium lacus* (99%)<br>-*Microbacterium invictum* (99%)<br>-*Microbacterium oleivorans* (99%)<br>-*Microbacterium shaanxiense* (99%)<br>-*Microbacterium natoriense* (99%)<br>-*Microbacterium foliorum* (99%)	Clinical specimens Laffineur et al. (2003)<br>Phyllosphere Behrendt et al. (2001)<br>Clinical specimens Gneiding et al. (2008)<br>Potato leaves Morohoshi et al. (2011)<br>Sea water and marine mud Takeuchi and Hatano (1998)<br>Marine environment Kageyama et al. (2007)<br>Compost Vaz-Moreira et al. (2009)<br>Oil-containing environments Schippers et al. (2005)<br>Soybean Peng et al. (2015)<br>Soil Liu et al. (2005)<br>Phyllosphere Behrendt et al. (2001)	Actinomycetales	Genus already known from ***Manduca sexta***: van der Hoeven et al 2008, referred as <u>*Microbacterium* sp. #3</u>
#4.	OP630950	484 bp	-*Enterococcus rivorum (100%)*<br>-*Enterococcus haemoperoxidus (100%)*<br>-*Enterococcus plantarum (100%)*<br>-*Enterococcus faecalis (100%)*<br><br><br>-*Enterococcus termitis (100%)*<br>-*Enterococcus moraviensis (99%)*<br>-*Enterococcus ureasiticus (99%)*<br>-*Enterococcus rotai (99%)*<br>-*Enterococcus silesiacus (99%)*	Water Niemi et al. (2012)<br>Water Svec et al. (2001)<br>Plants Švec et al. (2012)<br>**Many Insects including Lepidoptera** see above, Marti (1972)<br>Gut of ***Manduca sexta***, ***Drosophila*** Cox and Gilmore (20(<br>Clinical specimens Mason et al. (2011)<br>Gut of a **termite** Švec et al. (2006)<br>Water Svec et al. (2001)<br>Water Sistek et al. (2012)<br>Water, plant, **mosquito** Sedláček, I., et al. (2013)<br>Water Švec et al. (2006)	Lactobacillales	Genus already known from ***Manduca sexta***: Brinkman<br>**Many Insects including Lepidoptera** see above, Mart (1972),<br>referred as <u>*Enterococcus sp. #4*</u>
#5.	OP630951	294 bp	-*Comamonas kersterii (98%)*<br><br>-*Acidovorax sp*. (98%)	Soil Swamy et al (2013)<br>Clinical specimens Almuzara et al. (2017)<br><br>Species of different plant, soil and water samples (Gardan et al 2003, Choi et al 2010, Li et al 2011, Schaad et al 2008, Chun et al 2017, Willems et al 1992, Pal et al 2018, Gardan et al 2000) and<br>clinical specimens GenBank: KY014106.1		

References listed in Table S11:

Ahmad, A., Ghosh, A., Schal, C., Zurek, L., 2011. Insects in confined swine operations carry a large antibiotic resistant and potentially virulent enterococcal community. BMC Microbiology 11, 23.

Almuzara, M., Barberis, C., Veiga, F., Bakai, R., Cittadini, R., Ocampo, C.V., Serena, M.A., Cohen, E., Ramirez, M., Famiglietti, A., 2017. Unusual presentations of Comamonas kerstersii infection. New microbes and new infections 19, 91-95.

Behrendt, U., Ulrich, A., Schumann, P., 2001. Description of Microbacterium foliorum sp. nov. and Microbacterium phyllosphaerae sp. nov., isolated from the phyllosphere of grasses and the surface litter after mulching the sward, and reclassification of Aureobacterium resistens (Funke et al. 1998) as Microbacterium resistens comb. nov. International journal of systematic and evolutionary microbiology 51, 1267-1276.

Biswas, J.S., Fitchett, J., O'Hara, G., 2014. Comamonas kerstersii and the Perforated Appendix. Journal of Clinical Microbiology 52, 3134-3134.

Choi, J.-H., Kim, M.-S., Roh, S.W., Bae, J.-W., 2010. Acidovorax soli sp. nov., isolated from landfill soil. International journal of systematic and evolutionary microbiology 60, 2715-2718.

Chun, S.-J., Cui, Y., Ko, S.-R., Lee, H.-G., Srivastava, A., Oh, H.-M., Ahn, C.-Y., 2017. Acidovorax lacteus sp. nov., isolated from a culture of a bloom-forming cyanobacterium (Microcystis sp.). Antonie van Leeuwenhoek 110, 1199-1205.

Cox, C.R., Gilmore, M.S., 2007. Native microbial colonization of Drosophila melanogaster and its use as a model of Enterococcus faecalis pathogenesis. Infection and immunity 75, 1565-1576.

Gardan, L., Dauga, C., Prior, P., Gillis, M., Saddler, G., 2000. Acidovorax anthurii sp. nov., a new phytopathogenic bacterium which causes bacterial leaf-spot of anthurium. International journal of systematic and evolutionary microbiology 50, 235-246.

Gardan, L., Stead, D.E., Dauga, C., Gillis, M., 2003. Acidovorax valerianellae sp. nov., a novel pathogen of lamb's lettuce [Valerianella locusta (L.) Laterr.]. International journal of systematic and evolutionary microbiology 53, 795-800.

Gneiding, K., Frodl, R., Funke, G., 2008. Identities of Microbacterium spp. encountered in human clinical specimens. Journal of clinical microbiology 46, 3646-3652.

Kageyama, A., Takahashi, Y., Matsuo, Y., Adachi, K., Kasai, H., Shizuri, Y., Omura, S., 2007. Microbacterium flavum sp. nov. and Microbacterium lacus sp. nov., isolated from marine environments. Actinomycetologica 21, 53-58.

Laffineur, K., Avesani, V., Cornu, G., Charlier, J., Janssens, M., Wauters, G., Delmée, M., 2003. Bacteremia Due to a Novel Microbacterium Species in a Patient with Leukemia and Description of Microbacterium paraoxydans sp. nov. Journal of clinical microbiology 41, 2242-2246.

Li, D., Rothballer, M., Schmid, M., Esperschütz, J., Hartmann, A., 2011. Acidovorax radicis sp. nov., a wheat-root-colonizing bacterium. International journal of systematic and evolutionary microbiology 61, 2589-2594.

Liu, J., Nakayama, T., Hemmi, H., Asano, Y., Tsuruoka, N., Shimomura, K., Nishijima, M., Nishino, T., 2005. Microbacterium natoriense sp. nov., a novel D-aminoacylase-producing bacterium isolated from soil in Natori, Japan. International journal of systematic and evolutionary microbiology 55, 661-665.

Martin, J.D., Mundt, J.O., 1972. Enterococci in insects. Applied microbiology 24, 575-580.

Morohoshi, T., Wang, W.-Z., Someya, N., Ikeda, T., 2011. Genome sequence of Microbacterium testaceum StLB037, an N-acylhomoserine lactone-degrading bacterium isolated from potato leaves. Journal of bacteriology 193, 2072-2073.

Naser, S.M., Vancanneyt, M., Hoste, B., Snauwaert, C., Vandemeulebroecke, K., Swings, J., 2006. Reclassification of Enterococcus flavescens Pompei et al. 1992 as a later synonym of Enterococcus casseliflavus (ex Vaughan et al. 1979) Collins et al. 1984 and Enterococcus saccharominimus Vancanneyt et al. 2004 as a later synonym of Enterococcus italicus Fortina et al. 2004. International journal of systematic and evolutionary microbiology 56, 413-416.

Niemi, R.M., Ollinkangas, T., Paulin, L., Švec, P., Vandamme, P., Karkman, A., Kosina, M., Lindström, K., 2012. Enterococcus rivorum sp. nov., from water of pristine brooks. International journal of systematic and evolutionary microbiology 62, 2169-2173.

Opota, O., Ney, B., Zanetti, G., Jaton, K., Greub, G., Prod'hom, G., 2014. Bacteremia Caused by Comamonas kerstersii in a Patient with Diverticulosis. Journal of Clinical Microbiology 52, 1009-1012.
Pal, D., Kaur, N., Sudan, S.K., Bisht, B., Krishnamurthi, S., Mayilraj, S., 2018. Acidovorax kalamii sp. nov., isolated from a water sample of the river Ganges. International journal of systematic and evolutionary microbiology.
Peng, S., Dongying, L., Bingxin, Y., Mingjun, L., Gehong, W., 2015. Microbacterium shaanxiense sp. nov., isolated from the nodule surface of soybean. International journal of systematic and evolutionary microbiology 65, 1437-1443.
Reid, K.C., Cockerill, I.I.I.F.R., Patel, R., 2001. Clinical and Epidemiological Features of Enterococcus casseliflavus/flavescens and Enterococcus gallinarum Bacteremia: A Report of 20 Cases. Clinical Infectious Diseases 32, 1540-1546.
Schaad, N.W., Postnikova, E., Sechler, A., Claflin, L.E., Vidaver, A.K., Jones, J.B., Agarkova, I., Ignatov, A., Dickstein, E., Ramundo, B.A., 2008. Reclassification of subspecies of Acidovorax avenae as A. Avenae (Manns 1905) emend., A. cattleyae (Pavarino, 1911) comb. nov., A. citrulli Schaad et al., 1978) comb. nov., and proposal of A. oryzae sp. nov. Systematic and Applied Microbiology 31, 434-446.
Schippers, A., Bosecker, K., Spröer, C., Schumann, P., 2005. Microbacterium oleivorans sp. nov. and Microbacterium hydrocarbonoxydans sp. nov., novel crude-oil-degrading Gram-positive bacteria. International journal of systematic and evolutionary microbiology 55, 655-660.
Sedláček, I., Holochová, P., Mašlaňová, I., Kosina, M., Spröer, C., Bryndová, H., Vandamme, P., Rudolf, I., Hubálek, Z., Švec, P., 2013. Enterococcus ureilyticus sp. nov. and Enterococcus rotai sp. nov., two urease-producing enterococci from the environment. International journal of systematic and evolutionary microbiology 63, 502-510.
Shin, N.-R., Jung, M.-J., Kim, M.-S., Roh, S.W., Nam, Y.-D., Bae, J.-W., 2011. Corynebacterium nuruki sp. nov., isolated from an alcohol fermentation starter. International journal of systematic and evolutionary microbiology 61, 2430-2434.
Sistek, V., Maheux, A.F., Boissinot, M., Bernard, K.A., Cantin, P., Cleenwerck, I., De Vos, P., Bergeron, M.G., 2012. Enterococcus ureasiticus sp. nov. and Enterococcus quebecensis sp. nov., isolated from water. International journal of systematic and evolutionary microbiology 62, 1314-1320.
Svec, P., Devriese, L.A., Sedlácek, I., Baele, M., Vancanneyt, M., Haesebrouck, F., Swings, J., Doskar, J., 2001. Enterococcus haemoperoxidus sp. nov. and Enterococcus moraviensis sp. nov., isolated from water. International Journal of Systematic and Evolutionary Microbiology 51, 1567-1574.
Švec, P., Vancanneyt, M., Sedláček, I., Naser, S.M., Snauwaert, C., Lefebvre, K., Hoste, B., Swings, J., 2006. Enterococcus silesiacus sp. nov. and Enterococcus termitis sp. nov. International journal of systematic and evolutionary microbiology 56, 577-581.
Švec, P., Vandamme, P., Bryndová, H., Holochová, P., Kosina, M., Mašlaňová, I., Sedláček, I., 2012. Enterococcus plantarum sp. nov., isolated from plants. International journal of systematic and evolutionary microbiology 62, 1499-1505.
Swamy, M., K Pradeep, S., Sandip, P., Monika, P., Neeki, M., Nabendu, N., 2013. Scholars Research Library Identification and characterization of a novel isolate Comomonas kerstersii KSM7 for extracellular α-amylase production.
Takeuchi, M., Hatano, K., 1998. Proposal of six new species in the genus Microbacterium and transfer of Flavobacterium marinotypicum ZoBell and Upham to the genus Microbacterium as Microbacterium maritypicum comb. nov. International Journal of Systematic and Evolutionary Microbiology 48, 973-982.
Vaz-Moreira, I., Lopes, A.R., Faria, C., Spröer, C., Schumann, P., Nunes, O.C., Manaia, C.M., 2009. Microbacterium invictum sp. nov., isolated from homemade compost. International journal of systematic and evolutionary microbiology 59, 2036-2041.
Wauters, G., De Baere, T., Willems, A., Falsen, E., Vaneechoutte, M., 2003. Description of Comamonas aquatica comb. nov. and Comamonas kerstersii sp. nov. for two subgroups of Comamonas terrigena and emended description of Comamonas terrigena. International journal of systematic and evolutionary microbiology 53, 859-862.
Willems, A., Goor, M., Thielemans, S., Gillis, M., Kersters, K., De Ley, J., 1992. Transfer of several phytopathogenic Pseudomonas species to Acidovorax as Acidovorax avenae subsp. avenae subsp. nov., comb. nov., Acidovorax avenae subsp. citrulli, Acidovorax avenae subsp. cattleyae, and Acidovorax konjaci. International Journal of Systematic and Evolutionary Microbiology 42, 107-119.

Table S12: Isolated bacteria grown on selective media and antimicrobial susceptibility testing.

#	Origin	Name	STD1 pH 9	STD 1 pH 7	pH 7 (anoxic)	pH 9 (anoxic)	EMB	NBTA
#1	Feces**	*Enterococcus casseliflavus/gallinarum*	+++	+++	+++	++	-	+++ green
#2	Feces	*Corynebacterium nuruki*	+++	++	+++	++	-	++ green
#3	Feces	*Microbacterium sp.*	++	++	++	+	-	++ green-yellow
#4	Feces	*Enterococcus sp.*	-*	++	++	+	-	+ yellow
#5	Feces	*Comamonas kersterii/ -Acidovorax sp.*	+++	++	++	++	-	++ green

** also isolated from Egg

*= growes at pH 9 at RT

#	Mc	Starch pH7	Starch pH9	Cellulose pH7	Cellulose pH9	Catalase	Casein pH7	Casein pH9	Ultrapure water
#1	-	++	+	-	-	weak positive	+++	+++	-
#2	-	+++	+++	-	-	positive	+++	+++	-
#3	-	++	+	-	-	positive	-	-	-
#4	-	-	-	+	-	weak positive	-	-	-
#5	-	++	+++	-	-	weak positive	-	-	-

Antimicrobial susceptibility testing: zone of inhibition in cm

#	Streptomycin 1.000 U/m	Penicillin 500 µg/ml	Gentamicin 100 µg/ml	Amphotericin B 500 mg/ml	Kanamycin 500 µg/ml	Ampicillin 100 µg/ml	Lysozyme 1×10^4µg/ml	Hemoly -mph+*	Hemoly -mph-**
#1	2.4	3.6	2.1	-	2	2.7	1	1	1
#2	2.3	3.1	2.1	-	2	2.7	1	1	1
#3	2	>3.2	2.2	-	3	>5	1	1	1
#4	1.9	2.9	2.2	-	1	2.3	-	-	-
#5	-	2.1	1.5	-	2	2.7	1	1	1

*immune-activated

** not immune-activated

Antimicrobial susceptibility testing: zone of inhibition in cm

#	Supernatant	10% HCOl	5% HOCl	4% HOCl	3% HOCl	2,5% HOCL	2% HOCl	1,5% HOCl	1% HOCl	0,5% HOCl	0,1% HOCl
#1	2.5 (inhibits #3)	1.9	1.6	1.6	1.5	1.5	1.4	1.3	1.2	1	0.9
#2	2.4 (inhibits #3)	1.9	2.55	2	1.9	1.6	1.4	1.3	1.2	1.4	1
#3	-	2.1	2.1	1.9	1.6	1.5	1.6	1.5	1.4	1.4	1.3
#4	1.1 (inhibits #3)	1.9	1.3	1.3	1.2	1.1	1.1	1.1	1.1	1.1	-
#5	-	1.8	1.1	1.1	1.1	1.1	1.05	1.05	1.05	0.9	-

Table S13: Gut wall thicknesses after *Microbacterium* sp. and *Enterococcus* sp. (#4) treatments.

Test details	Mean 1 [mm]	Mean 2 [mm]	Mean Diff [mm]	SE of diff.
control vs. *Microbacterium* sp.	0.8771	1.691	**-0.8138**	0.05338
control vs. *Enterococcus* sp. (#4)	0.8771	1.183	**-0.3054**	0.06225
Microbacterium sp.vs. *Enterococcus* sp. (#4)	1.691	1.183	**0.5084**	0.06290

Table S14: Effect size of the used treatments with the respective imaging modality; eta squared (η²), Hedges' g or Cohen's d).

Imaging modality	Diagnostic finding	Treatment	Effect size of treatment η², Hedges' *g* or Cohen's *d* (compared to control)	Large effect*	Moderate effect*	Small effect*	No effect*
CT	CT gut wall thickness (post-iodixanol)	Bt 12h	$\eta^2 = 0.6010$	X			
			$g = 3.38151$	X			
CT	CT gut wall thickness (post-iodixanol)	Bt 36h	$\eta^2 = 0.5059$	X			
			$g = 1.985085$	X			
CT	CT gut wall thickness (post-iodixanol)	DSS 5%	$\eta^2 = 0.3801$	X			
			$g = 1.526288$	X			
CT	CT gut wall thickness (post-iodixanol)	Uracil 0.1M	$\eta^2 = 0.4951$	X			
			$g = 1.93884$	X			
CT	CT gut wall thickness (post-iodixanol)	Uracil 1.0M	$\eta^2 = 0.5273$	X			
			$g = 2.022548$	X			
CT	CT gut wall thickness (post-iodixanol)	*Microbacterium sp.* (#3)	$\eta^2 = 0.8648$	X			
			$g = 5.971387$	X			
CT	CT gut wall thickness (post-iodixanol)	*Enterococcus sp.* (#4)	$\eta^2 = 0.5675$	X			
			$g = 2.308153$	X			
CT	CT gut wall thickness (post-iodixanol)	*E. coli*	$\eta^2 = 0.01557$				X
			$d = 0.244981$				X
CT	CT signal density (post-iodixanol)	Bt 12h	$\eta^2 = 0.2707$	X			
			$d = 1.165844$	X			
CT	CT signal density (post-iodixanol)	Bt 36h	$\eta^2 = 0.8221$	X			
			$g = 4.128445$	X			
CT	CT signal density (post-iodixanol)	DSS 5%	$\eta^2 = 0.2188$		X		
			$g = 1.017681$	X			
CT	CT signal density (post-iodixanol)	Uracil 0.1M	$\eta^2 = 0.4309$	X			
			$g = 1.752486$	X			
CT	CT signal density (post-iodixanol)	*E. coli*	$\eta^2 = 0.1931$	X			
			$g = 0.95736$	X			
MRI	MR gut wall thickness (post-gadolinium)	Bt 12h	$\eta^2 = 0.4014$	X			
			$g = 1.656781$	X			
MRI	MR gut wall thickness (post-gadolinium)	Bt 36h	$\eta^2 = 0.7520$	X			
			$g = 3.521243$	X			
MRI	MR gut wall thickness (post-gadolinium)	DSS 5%	$\eta^2 = 0.3869$	X			
			$g = 2.02662$	X			
MRI	MR gut wall thickness (post-gadolinium)	Uracil 0.1M	$\eta^2 = 0.3492$	X			
			$g = 1.534149$	X			
MRI	MR gut wall thickness (post-gadolinium)	Uracil 1.0M	$\eta^2 = 0.5273$	X			
			$g = 2.022548$	X			
MRI	MR gut wall thickness (post-gadolinium)	*E. coli*	$\eta^2 = 0.09932$		X		
			$g = 0.726905$		X		
MRI	MR normalized T1 signal	Bt 12h	$\eta^2 = 0.4017$	X			
			$g = 1.596851$	X			
MRI	MR normalized T1 signal	Bt 36h	$\eta^2 = 0.9196$	X			
			$g = 6.712817$	X			
MRI	MR normalized T1 signal	DSS 5%	$\eta^2 = 0.07492$		X		
			$g = 0.588844$		X		
MRI	MR normalized T1 signal	Uracil 0.1M	$\eta^2 = 0.2248$	X			
			$g = 1.04751$	X			
MRI	MR normalized T1 signal	*E. coli*	$\eta^2 = 0.0006059$				X
			$g = 0.046986$				X
MRI	MR T2 signal	Bt 12h	$\eta^2 = 0.1857$	X			
			$g = 0.933256$	X			
MRI	MR T2 signal	Bt 36h	$\eta^2 = 0.4704$	X			
			$g = 1.826791$	X			

MRI	MR T2 signal	DSS 5%	$\eta^2 = 0.03676$			X	
			g = 0.386134			X	
FDG-PET	$PUV_{Max\ norm}$	Bt 3 h	$\eta^2 = 0.1124$			X	
			g = 0.702109			X	
FDG-PET	$PUV_{Max\ norm}$	Bt 12h	$\eta^2 = 0.1893$	X			
			g = 0.966266	X			
FDG-PET	$PUV_{Max\ norm}$	Bt 24h	$\eta^2 = 0.2314$	X			
			g = 1.093209	X			
FDG-PET	$PUV_{Max\ norm}$	DSS 5%	$\eta^2 = 0.1607$	X			
			g = 0.886772	X			
FDG-PET	$PUV_{Max\ norm}$	Uracil 0.1M	$\eta^2 = 0.1609$	X			
			g = 0.87674	X			
FDG-PET	$PUV_{Max\ norm}$	Uracil 1.0M	$\eta^2 = 0.001127$				X
			g = 0.06767				X
FDG-PET	$PUV_{Max\ norm}$	*Microbacterium sp.* (#3)	$\eta^2 = 0.1046$		X		
			g = 0.697976		X		
FDG-PET	$PUV_{Max\ norm}$	*Enterococcus sp.* (#4)	$\eta^2 = 0.004313$				X
			g = 0.146927				X
FDG-PET	$PUV_{Max\ norm}$	*E. coli*	$\eta^2 = 0.07862$		X		
			g = 0.592084		X		

***η^2= 0.01 small effect, 0.06 moderate effect and 0.14 a large effect size**
***d/g = 0.2 small effect, 0.5 moderate effect and 0.8 a large effect size**

Table S15: Effect size of the used antibiotic treatments with the respective imaging modality; eta squared (η^2), Hedges' g or Cohen's d.

Imaging modality	Diagnostic finding	Treatment	Effect size of treatment η^2, Hedges' *g* or Cohen's *d* (compared to *Bt* only)	Large effect*	Moderate effect*	Small effect*	No effect*
CT	CT gut wall thickness (post-iodixanol)	*Bt* + gentamicin 0.5 mg/ml	$\eta^2 = 0.08357$		X		
			g = 0.661769		X		
CT	CT gut wall thickness (post-iodixanol)	*Bt* + gentamicin 1 mg/ml	$\eta^2 = 0.3171$	X			
			g = 1.59552	X			
MRI	MR gut wall thickness (post-gadolinium)	*Bt* + gentamicin 0.5 mg/ml	$\eta^2 = 0.1850$	X			
			g = 1.060247	X			
MRI	MR gut wall thickness (post-gadolinium)	*Bt* + gentamicin 1 mg/ml	$\eta^2 = 0.3171$	X			
			g = 1.616842	X			

***η^2= 0.01 small effect, 0.06 moderate effect and 0.14 a large effect size**
***d/g = 0.2 small effect, 0.5 moderate effect and 0.8 a larg effect size**

Table S16: Effect size of the used anti-ROS treatments (DUOX) with the respective imaging modality; eta squared (η^2), Hedges' g or Cohen's d.

Imaging modality	Diagnostic finding	Treatment	Effect size of treatment η^2, Hedges' *g* or Cohen's *d* (compared to uracil only)	Large effect*	Moderate effect*	Small effect*	No effect*
CT	CT gut wall thickness (post-iodixanol)	Uracil + DPI	$\eta^2 = 0.5555$	X			
			d = 2.182477	X			
CT	CT gut wall thickness (post-iodixanol)	Uracil + NAC	$\eta^2 = 0.5486$	X			
			g = 2.153977	X			
CT	CT signal density (post-iodixanol)	Uracil + DPI	$\eta^2 = 0.4111$	X			
			g = 1.633457	X			
CT	CT signal density (post-iodixanol)	Uracil + NAC	$\eta^2 = 0.3929$	X			
			g = 1.573131	X			
MRI	MR gut wall thickness (post-gadolinium)	Uracil + DPI	$\eta^2 = 0.1449$	X			
			g = 0.813261	X			
MRI	MR gut wall thickness (post-gadolinium)	Uracil + NAC	$\eta^2 = 0.3904$	X			
			g = 1.558285	X			
MRI	MR normalized T1 signal	Uracil + DPI	$\eta^2 = 0.1380$		X		
			g = 0.801297	X			

MRI	MR normalized T1 signal	Uracil + NAC	η^2 = 0.1397		X		
			g = 0.783295		X		
FDG-PET	$PUV_{Max\ norm}$	Uracil + DPI	η^2 = 0.2312	X			
			g = 1.088819	X			
FDG-PET	$PUV_{Max\ norm}$	Uracil + NAC	η^2 = 0.1299		X		
			g = 0.754842		X		

**η^2= 0.01 small effect, 0.06 moderate effect and 0.14 a large effect size*
**d/g = 0.2 small effect, 0.5 moderate effect and 0.8 a large effect size*

Table S17 Effect size of the used dexamethasone rescue after uracil treatment; eta squared (η^2), Hedges' g or Cohen's d.

Imaging modality	Diagnostic finding	Treatment	Effect size of treatment η^2, Hedges' *g* or Cohen's *d* (compared to uracil +NaCl)	Large effect*	Moderate effect*	Small effect*	No effect*
CT	CT gut wall thickness (post-iodixanol)	Uracil + DEX	η^2 = 0.6957	X			
			g = 2.88159	X			

**η^2= 0.01 small effect, 0.06 moderate effect and 0.14 a large effect size*
**d/g = 0.2 small effect, 0.5 moderate effect and 0.8 a large effect size*

References

1 Yamamoto, R. T. Mass Rearing of the Tobacco Hornworm. II. Larval Rearing and Pupation. *J. Econ. Entomol.* **62**, 1427-1431, (1969).

2 Müller, F. H. H., Farahati, J., Müller, A. G., Gillman, E. & Hentschel, M. Positron emission mammography in the diagnosis of breast cancer. Is maximum PEM uptake value a valuable threshold for malignant breast cancer detection? *Nuklearmedizin* **55**, 15-20, (2016).

3 Willott, E., Trenczek, T., Thrower, L. & Kanost, M. Immunochemical identification of insect hemocyte populations: monoclonal antibodies distinguish four major hemocyte types in *Manduca sexta*. *Eur. J. Cell Biol.* **65**, 417-423, (1994).

4 Levin, D. M., Breuer, L. N., Zhuang, S., Anderson, S. A., Nardi, J. B. & Kanost, M. R. A hemocyte-specific integrin required for hemocytic encapsulation in the tobacco hornworm, *Manduca sexta*. *Insect biochemistry and molecular biology* **35**, 369-380, (2005).

5 Ma, C. & Kanost, M. R. A β1, 3-glucan recognition protein from an insect, *Manduca sexta*, agglutinates microorganisms and activates the phenoloxidase cascade. *J. Biol. Chem.* **275**, 7505-7514, (2000).

6 von Bredow, C.-R., von Bredow, Y. M. & Trenczek, T. E. The larval haematopoietic organs of Manduca sexta (Insecta, Lepidoptera): an insight into plasmatocyte development and larval haematopoiesis. *Dev. Comp. Immunol.*, 103858, (2020).

7 von Bredow, Y. M., von Bredow, C. R. & Trenczek, T. E. A novel site of haematopoiesis and appearance and dispersal of distinct haemocyte types in the *Manduca sexta* embryo (Insecta, Lepidoptera). *Developmental & Comparative Immunology*, 103722, (2020).

8 Beetz, S., Brinkmann, M. & Trenczek, T. Differences between larval and pupal hemocytes of the tobacco hornworm, *Manduca sexta*, determined by monoclonal antibodies and density centrifugation. *Journal of insect physiology* **50**, 805-819, (2004).

9 Simon, P. Q-Gene: processing quantitative real-time RT-PCR data. *Bioinformatics* **19**, 1439-1440, (2003).

10 Laemmli, U. K. Cleavage of structural proteins during the assembly of the head of bacteriophage T4. *Nature* **227**, 680-685, (1970).

11 Sambrook, J., Fritsch, E. F. & Maniatis, T. *Molecular cloning: a laboratory manual*. (Cold spring harbor laboratory press, 1989).

12 Harlow, E. & Lane, D. A laboratory manual. *New York: Cold Spring Harbor Laboratory* **579**, (1988).

13 Chen, X., Lee, K.-A., Ren, X., Ryu, J.-C., Kim, G., Ryu, J.-H., Lee, W.-J. & Yoon, J. Synthesis of a highly HOCl-selective fluorescent probe and its use for imaging HOCl in cells and organisms. *Nat. Protoc.* **11**, 1219-1228, (2016).

14 Ma, J., Wang, S., Zhao, F. & Xu, J. Protein threading using context-specific alignment potential. *Bioinformatics* **29**, i257-i265, (2013).

15 Källberg, M., Wang, H., Wang, S., Peng, J., Wang, Z., Lu, H. & Xu, J. Template-based protein structure modeling using the RaptorX web server. *Nature protocols* **7**, 1511-1522, (2012).

16 Wang, Z., Zhao, F., Peng, J. & Xu, J. in *2010 IEEE International Conference on Bioinformatics and Biomedicine (BIBM).* 109-114 (IEEE).

17 Sun, J. Structures of mouse DUOX1–DUOXA1 provide mechanistic insights into enzyme activation and regulation. *Nat. Struct. Mol. Biol.*, (2020).

18 Wu, J.-X., Liu, R., Song, K. & Chen, L. Structures of human dual oxidase 1 complex in low-calcium and high-calcium states. *Nature Communications* **12**, 1-11.

19 Zhu, J., Wang, S., Bu, D. & Xu, J. Protein threading using residue co-variation and deep learning. *Bioinformatics* **34**, i263-i273, (2018).

20 Xu, J. Distance-based Protein Folding Powered by Deep Learning. *bioRxiv*, 465955, (2018).

21 Ma, J., Peng, J., Wang, S. & Xu, J. A conditional neural fields model for protein threading. *Bioinformatics* **28**, i59-i66, (2012).

22 Peng, J. & Xu, J. A multiple-template approach to protein threading. *Proteins* **79**, 1930-1939, (2011).

23 Wang, S., Ma, J., Peng, J. & Xu, J. Protein structure alignment beyond spatial proximity. *Sci. Rep.* **3**, 1448, (2013).

24 Wang, S., Ma, J., Peng, J. & Xu, J. Protein structure alignment beyond spatial proximity. *Sci. Rep.* **3**, 1448, (2013).

25 Wang, S., Peng, J. & Xu, J. Alignment of distantly related protein structures: algorithm, bound and implications to homology modeling. *Bioinformatics* **27**, 2537-2545, (2011).

26 Geissmann, Q. OpenCFU, a New Free and Open-Source Software to Count Cell Colonies and Other Circular Objects. *PLOS ONE* **8**, e54072, (2013).

27 Barghouthi, S. A. A universal method for the identification of bacteria based on general PCR primers. *Indian J Microbiol* **51**, 430-444, (2011).

28 Rainey, F. A., Ward-Rainey, N., Kroppenstedt, R. M. & Stackebrandt, E. The genus Nocardiopsis represents a phylogenetically coherent taxon and a distinct actinomycete lineage: proposal of Nocardiopsaceae fam. nov. *International Journal of Systematic and Evolutionary Microbiology* **46**, 1088-1092, (1996).

29 Colwell, R. K. EstimateS: Statistical estimation of species richness and shared species from samples. Version 9 and earlier. User’s Guide and application. *Published at: http://purl. oclc. org/estimates*, (2013).

30 Lee, S. S., Kim, A. Y., Yang, S.-K., Chung, J.-W., Kim, S. Y., Park, S. H. & Ha, H. K. Crohn disease of the small bowel: comparison of CT enterography, MR enterography, and small-bowel follow-through as diagnostic techniques. *Radiology* **251**, 751-761, (2009).

31 Louis, E., Ancion, G., Colard, A., Spote, V., Belaiche, J. & Hustinx, R. Noninvasive assessment of Crohn's disease intestinal lesions with 18F-FDG PET/CT. *J. Nucl. Med.* **48**, 1053-1059, (2007).

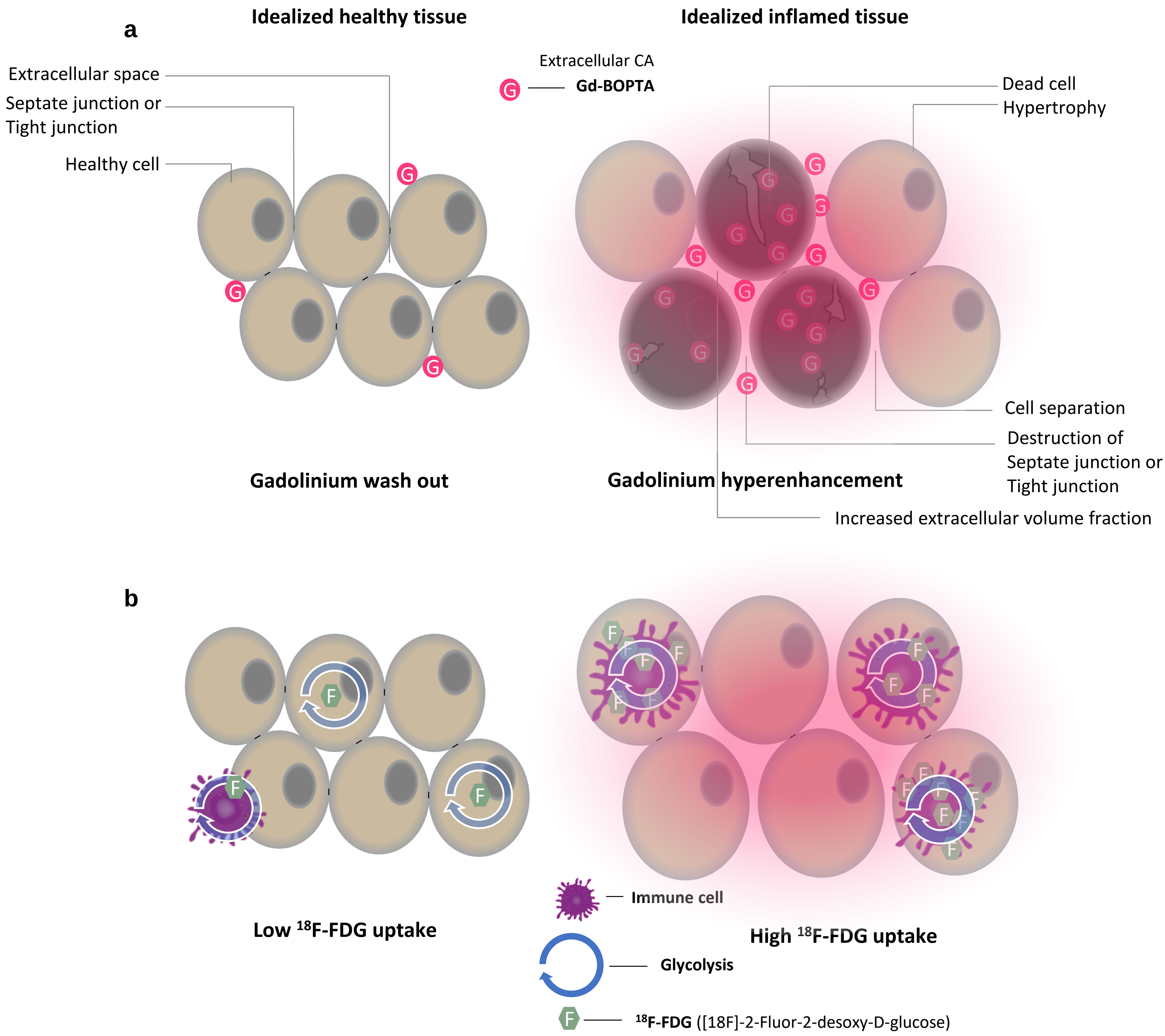

Fig. S1: Mode of action for extracellular CAs in CT, MRI, and PET **Supplementary Video 1** and **Supplementary Video 2** show idealized healthy and inflamed tissues with high CA uptake.

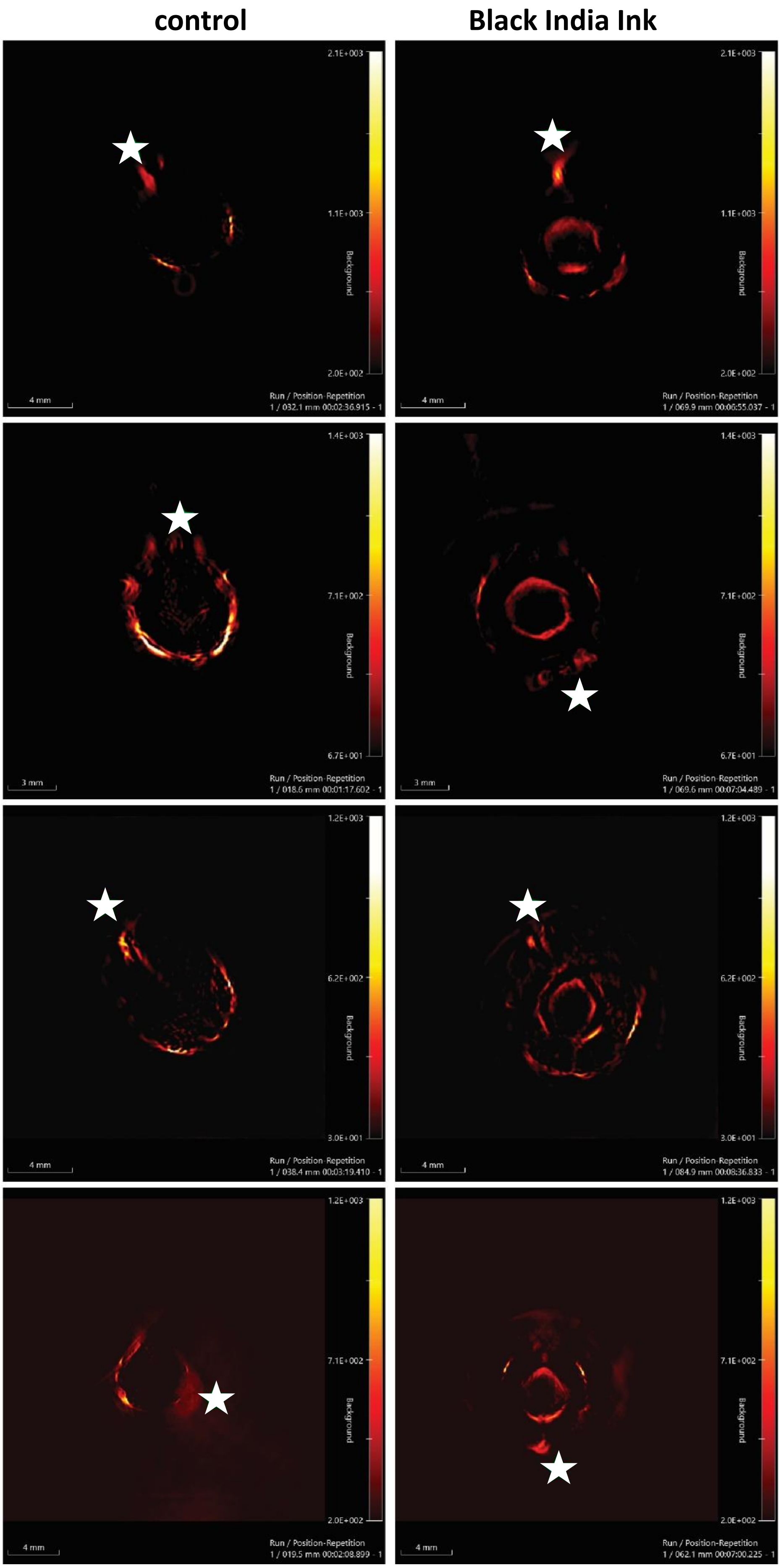

Fig. S2: Optoacoustic imaging without or with oral application of black India ink
Optoacoustic imaging without (left) or with oral application of black India ink (right). The star marks the position of the prolegs. After oral application of the CM, the midgut is apparent, n = 8.

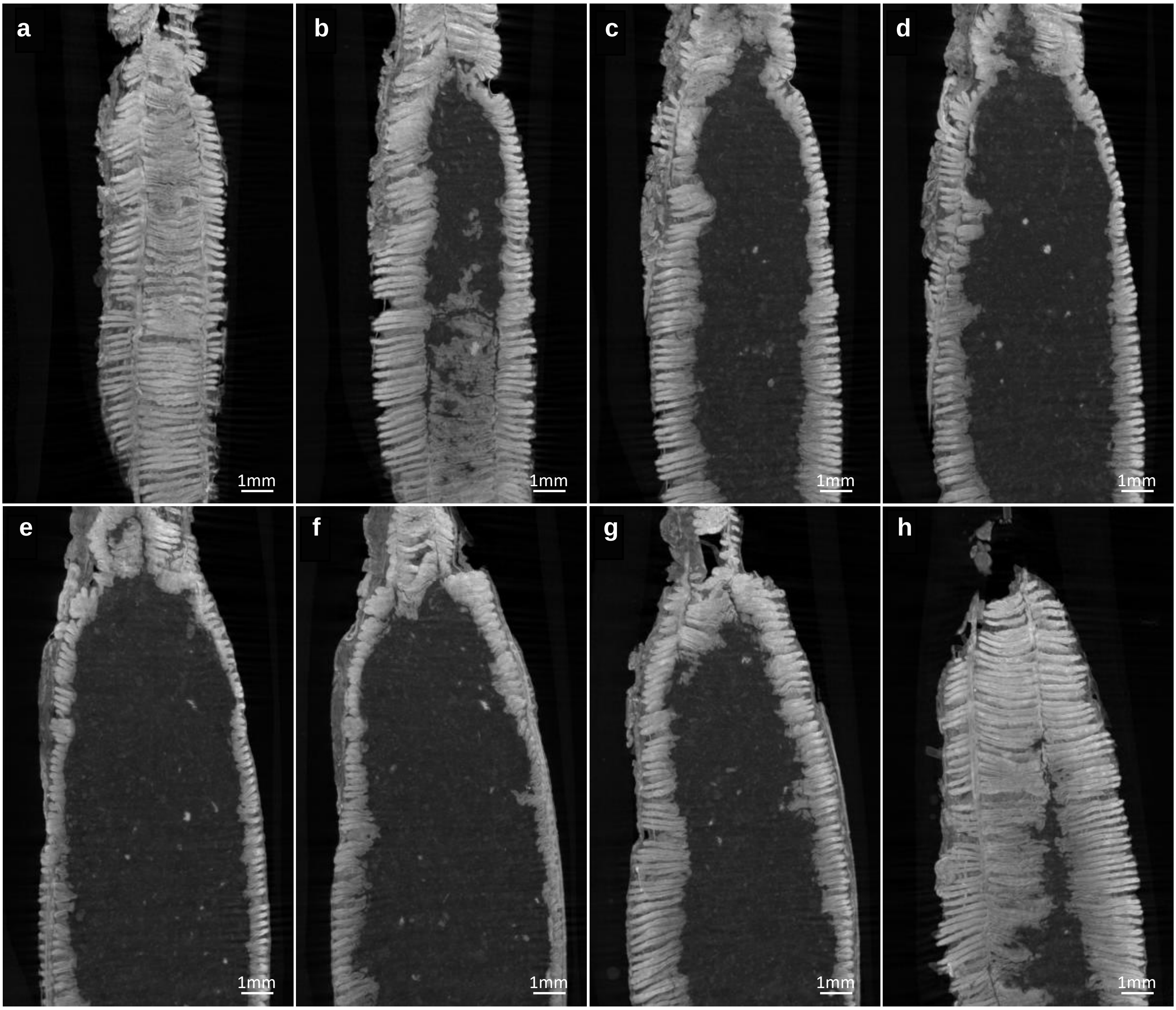

Fig. S3: Contrast-enhanced gut wall of *M. sexta* in µCT

Coronal *ex-vivo* contrast-enhanced µCT from *M. sexta* front-midgut in different planes (**a**–**h**).

CT

a

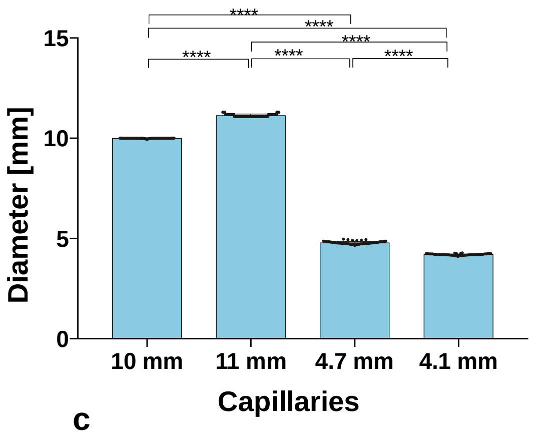

b

CT Standard Deviation and differences between different capillaries diameters.

Mann Whitney test of:	p value	SD of the first capillary [mm]	Median of the first capillary [mm]	Median of the second capillary [mm]	Difference: actual [mm]	Diference: Hodges-Lehman [mm]
10mm vs. 11mm	<0.0001	**0.0097**	10, n=109	11.15, n=113	1.1	1.1
10mm vs. 4.7mm	<0.0001	0.0097	10, n=109	4.79, n=69	-5.21	-5.21
10mm vs. 4.1mm	<0.0001	0.0097	10, n=109	4.21, n=48	-5.79	-5.79
11mm vs. 4.7mm	<0.0001	**0.0656**	11.15, n=113	4.79, n=69	-6.89	-6.92
4.1mm vs. 11mm	<0.0001	**0.0387**	4.21, n=48	11.15, n=113	6.31	6.35
4.7mm vs. 4.1mm	<0.0001	**0.0673**	4.79, n=69	4.21, n=48	-0.58	-0.58

Mean of the SDs = 0.0453 mm

c

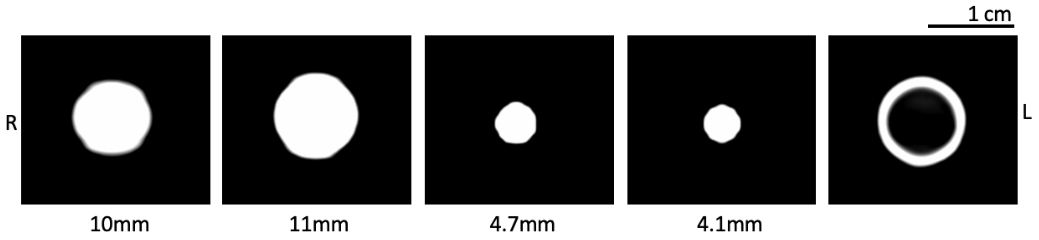

MRI

d

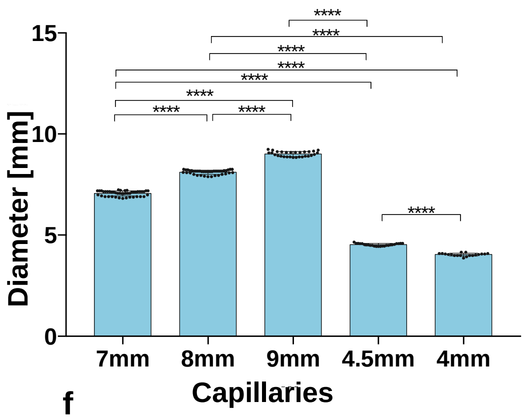

e

MRI Standard Deviation and differences between different capillaries diameters.

Mann Whitney test of:	p value	SD of the first capillary [mm]	Median of the first capillary [mm]	Median of the second capillary [mm]	Difference: actual [mm]	Diference: Hodges-Lehman [mm]
7mm vs. 8mm	<0.0001	**0.126**	7.1, n=45	8.14, n=40	1.04	1.04
7mm vs. 9mm	<0.0001	0.126	7.1, n=45	9.07, n=29	1.97	1.96
7mm vs. 4mm	<0.0001	0.126	7.1, n=45	4.04, n=19	-3.06	-3.06
7mm vs. 4.5mm	<0.0001	0.126	7.1, n=45	4.52, n=25	-2.58	-2.56
8mm vs. 9mm	<0.0001	**0.1066**	8.14, n=40	9.07, n=29	0.93	0.91
8mm vs. 4mm	<0.0001	0.1066	8.14, n=40	4.04, n=19	-4.1	-4.1
8mm vs. 4.5mm	<0.0001	0.1066	8.14, n=40	4.52, n=25	-3.62	-3.6
9mm vs. 4mm	<0.0001	**0.1255**	9.07, n=29	4.04, n=19	-5.03	-4.99
9mm vs. 4.5mm	<0.0001	0.1255	9.07, n=29	4.52, n=25	-4.55	-4.49
4mm vs. 4.5mm	<0.0001	**0.01245**	4.04, n=19	4.52, n=25	0.48	0.49
4.5mm		**0.01657**				

Mean of the SDs = 0.07756 mm

f

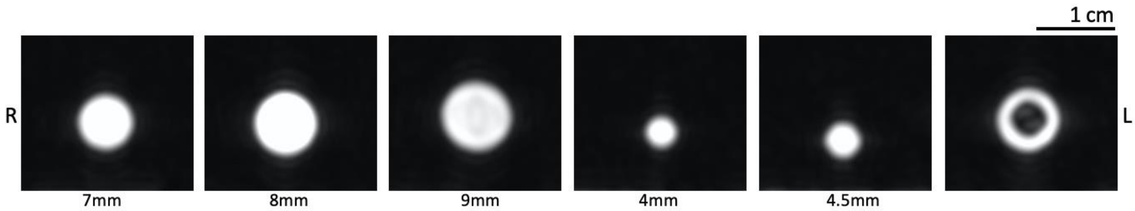

Fig. S4: Validation of CT and MRI resolution using phantoms (glass capillaries filled with CA)

(**a** and **d**) Outer diameter measurements of CT and MRI phantoms. (**b** and **e**) Descriptive statistics of (**a** and **d**). (**c** and **f**) Axial CT images of the phantoms filled with Iodixanol. Bar charts represent mean and SD. The following significance level have been used: Ns = P > 0.05, * = P ≤ 0.05, ** P ≤ 0.01, *** = P ≤ 0.001 and **** = P ≤ 0.0001. Source data are provided as a Source Data file.

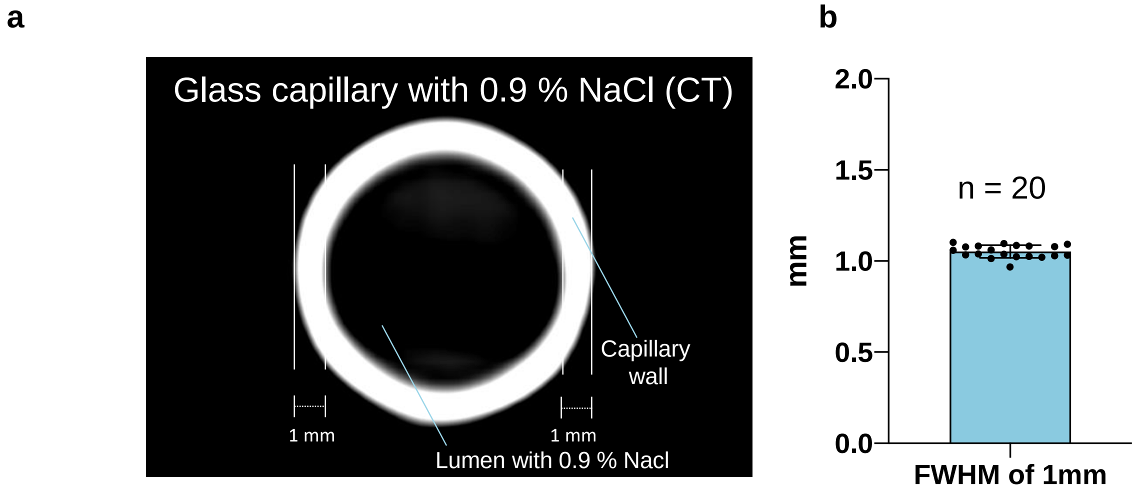

Fig. S5: Validation of CT resolution using a capillary glass phantom.
(**a**) CT image of the capillary glass phantom. (**b**) FWHM measurement of the capillary wall. The mean deviation of our measurements was 52 µm, and the double mean of the SD 0.07 mm. The bar chart represents the mean and SD. Source data are provided as a Source Data file.

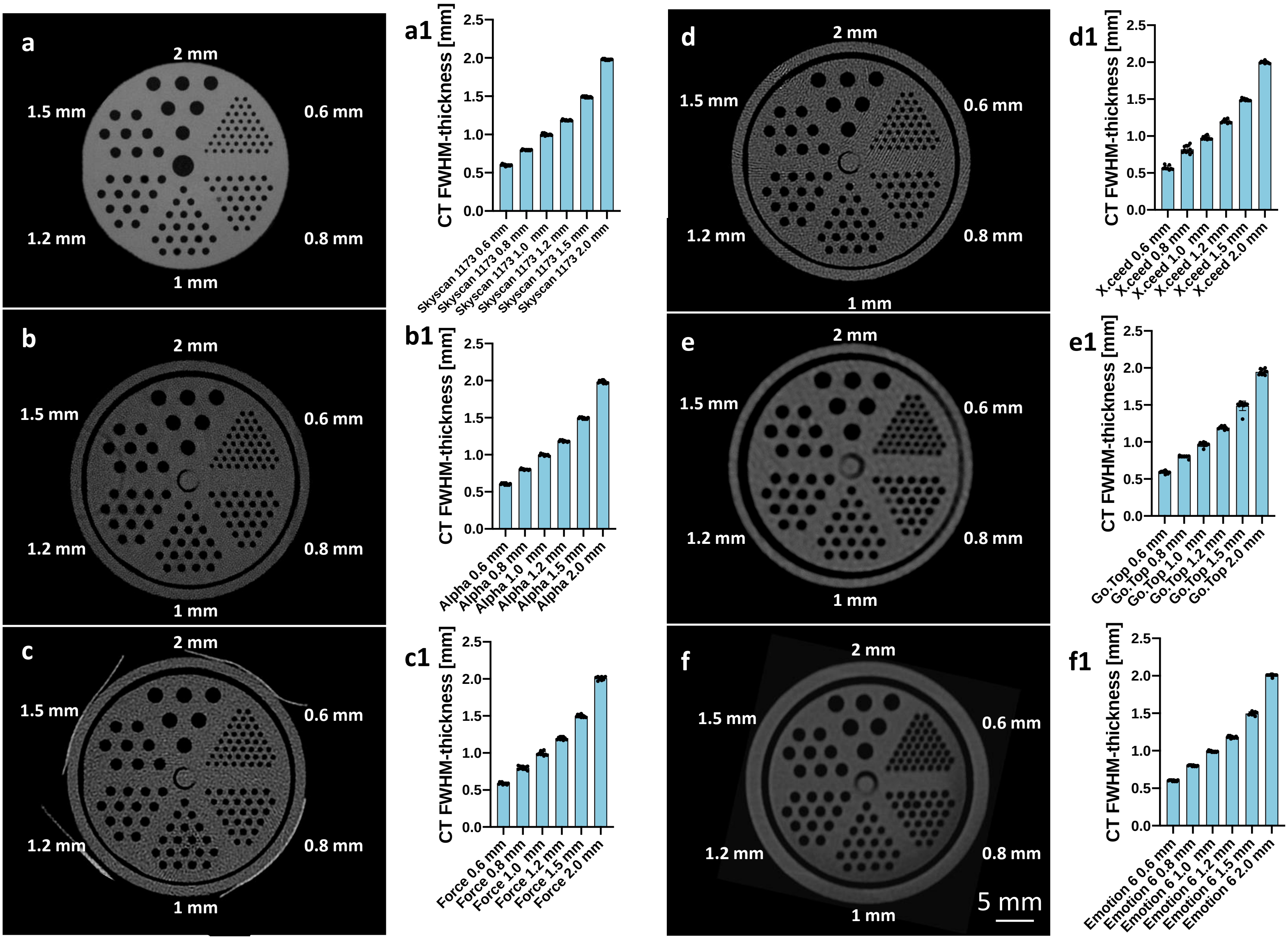

Fig. S6: 3D-printed Derenzo phantom in a micro CT or five different clinical CT scanners and the corresponding FWHM-thickness measurements of the pores. (**a-a1**) Derenzo phantom in the micro CT: Skyscan 1173, n = 68 (**b-b1**) the Photon-counting CT: NAEOTOM Alpha, n = 59 (**c-c1**) the Dual-source CT SOMATOM Force, n = 60 (**d-d1**), the signal source CT SOMATOM X.ceed, n = 59 (**e-e1**) the signal source CT SOMATOM go.Top (n = 60) and the signal source CT SOMATOM Emotion 6 (n = 60) (**f-f1**). Bar charts represent mean and SD. Source data are provided as a Source Data file.

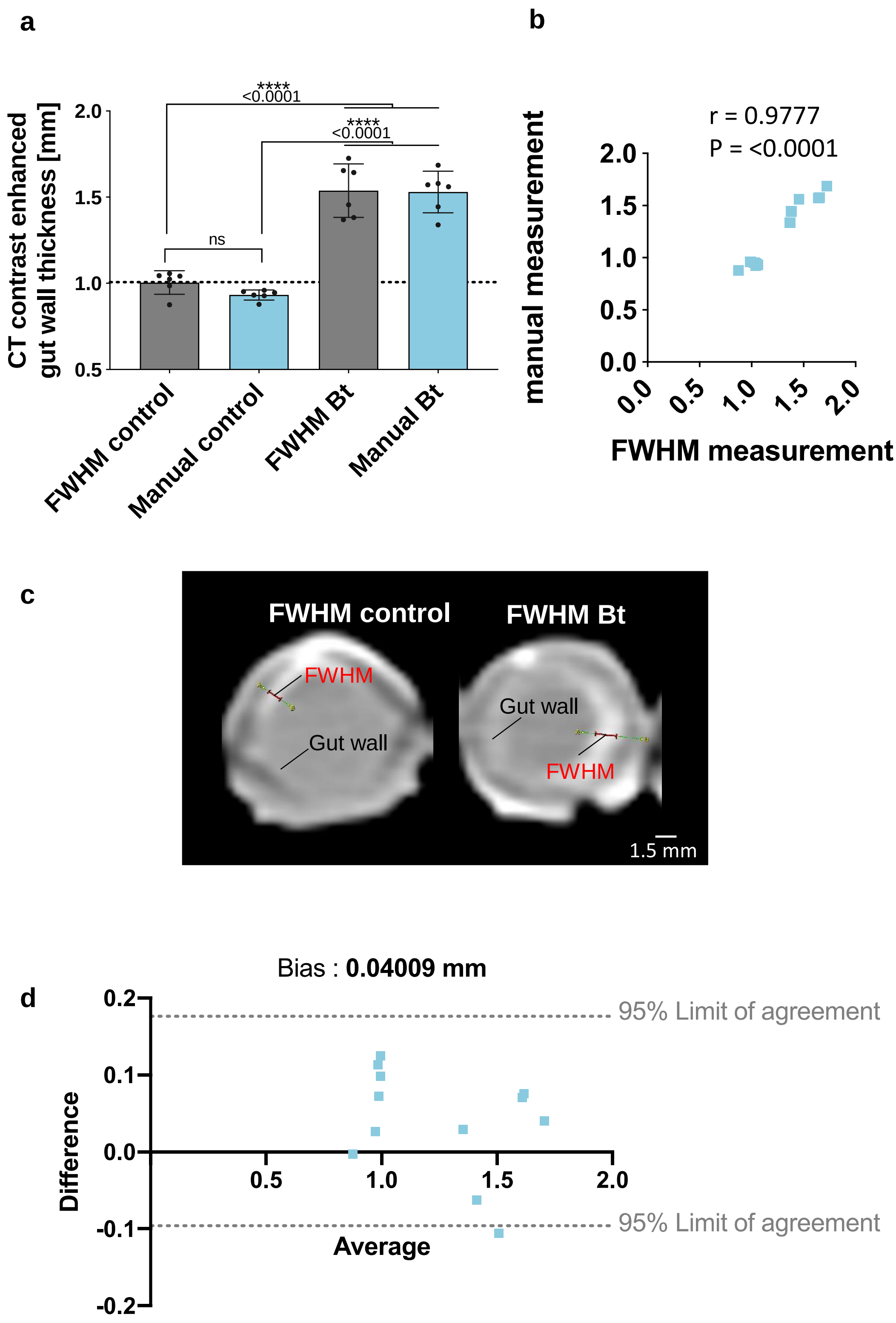

Fig. S7: Comparison of manual CT gut wall thickness measurement and FWHM measurement
(**a**) Comparison of manual CT gut wall thickness and FWHM measurements in control and Bt animals, n = 24, one-way ANOVA, $F(3,20) = 58.53$, $R^2 = 0.8977$, $P < 0.0001$. (**b**) Correlation of FWHM and manual measurements, n = 12, r = 0.9777, 95% CI = 0.9201-0.9939, $r^2 = 0.9559$ and two- tailed $P < 0.0001$. (**c**) CT images with FWHM mesurements. (**d**) Bland Altman plot comparing manual CT gut wall thickness and FWHM measurements (n = 12). Bar charts represent mean and SD. Every Data point represents a single animal. Source data are provided as a Source Data file.

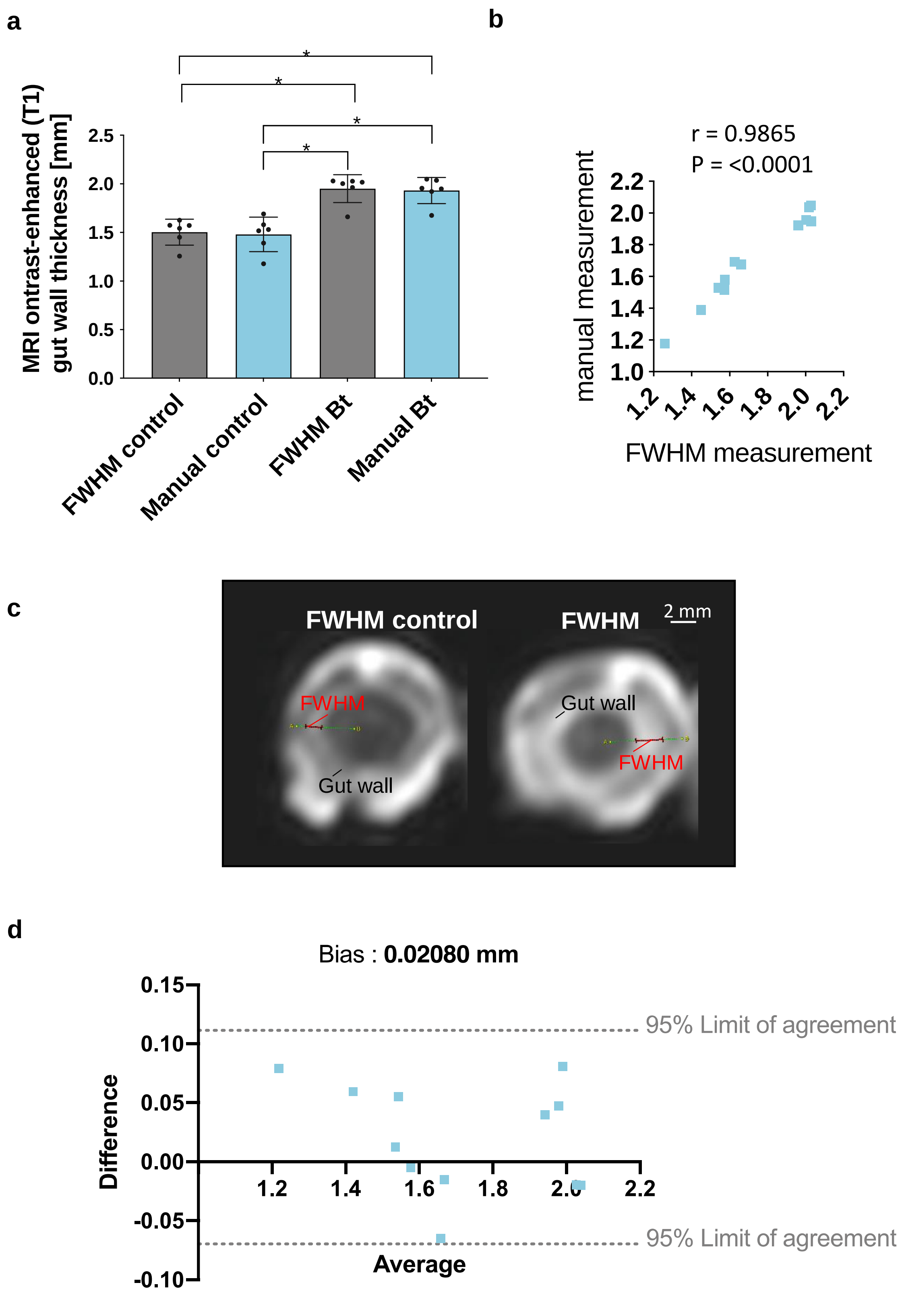

Fig. S8: Comparison of manual MRI gut wall thickness measurement and FWHM measurement
(**a**) Comparison of manual MRI gut wall thickness and FWHM measurements in control and Bt animals, (n = 24, Kruskal-Wallis-test = 16.37, P = 0.0010. (**b**) Correlation of FWHM and manual measurements, n = 12, r = 0.9865, 95% CI = 0.9511-0.9963, r^2 = 0.9732 and two- tailed P < 0.0001. (**c**) MR images with FWHM mesurements. (**d**) Bland Altman plot comparing manual MR gut wall thickness and FWHM measurements (n = 12). Bar charts represent mean and SD. Every Data point represents a single animal. Source data are provided as a Source Data file.

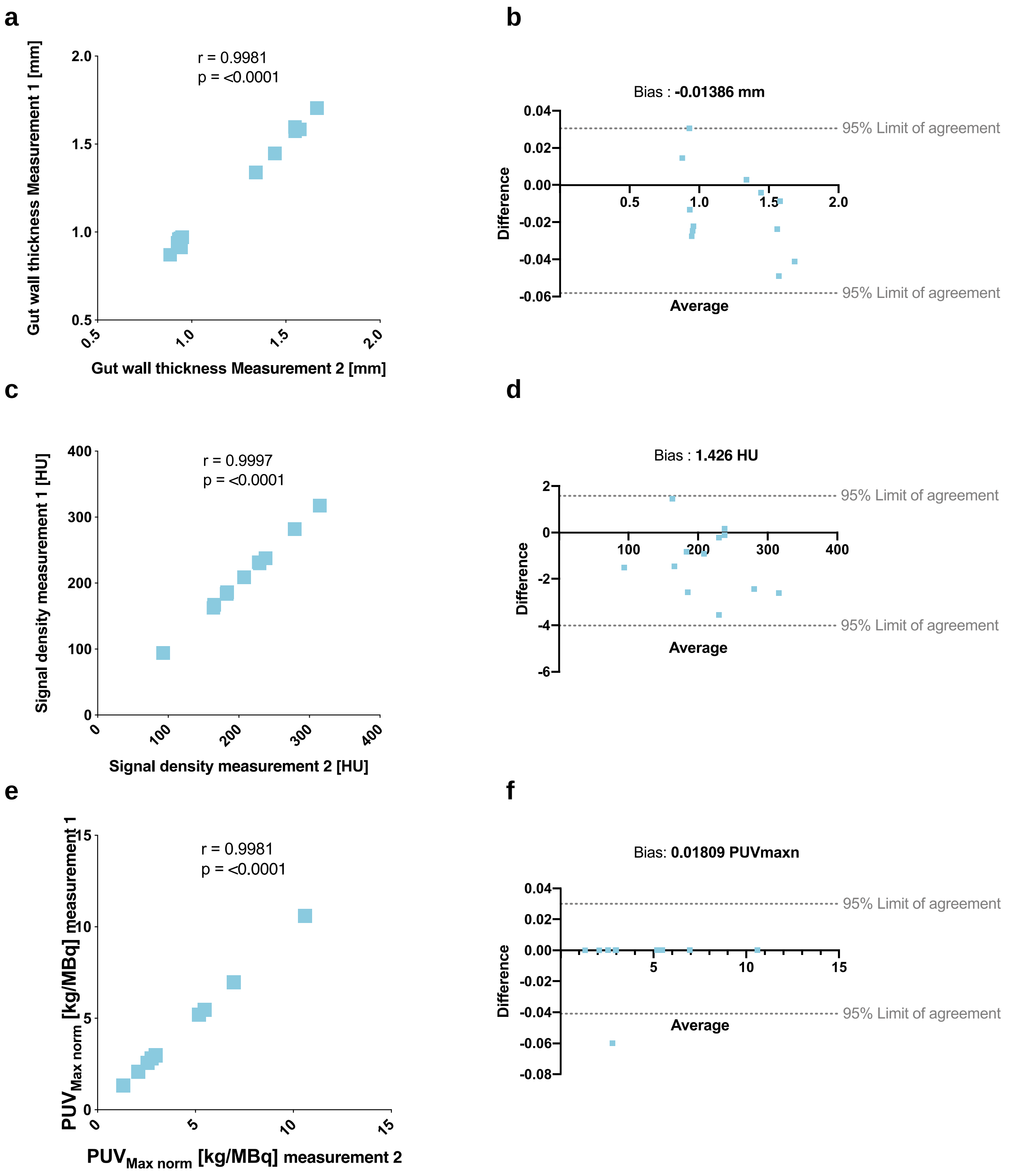

Fig. S9: Intraobserver variability and retesting in the used imaging modalities.
(**a**) Correlation of independent manual CT gut wall thickness measurements, n = 12, r = 0.9981, 95% CI = 0.9928-0.9995, r^2 = 0.9961 and two- tailed P < 0.0001. (**b**) Bland Altman plot comparing the manual CT gut wall thickness measurements (n = 12). (**c**) Correlation of two CT Signal density measurements, n = 12, r = 0.9997, 95% CI = 0.9990-0.9999, r^2 = 0.9994 and two- tailed P < 0.0001. (**d**) Bland Altman plot comparing the Signal density measurements (n = 12). (**e**) Correlation of two PUVMaxn measurements, n = 11, r = 1, 95% CI = 0.9999-1, r^2 = 1 and two- tailed P < 0.0001. (**f**) Bland Altman plot comparing the PUVMaxn measurements (n = 11). Every Data point represents a single animal. Source data are provided as a Source Data file.

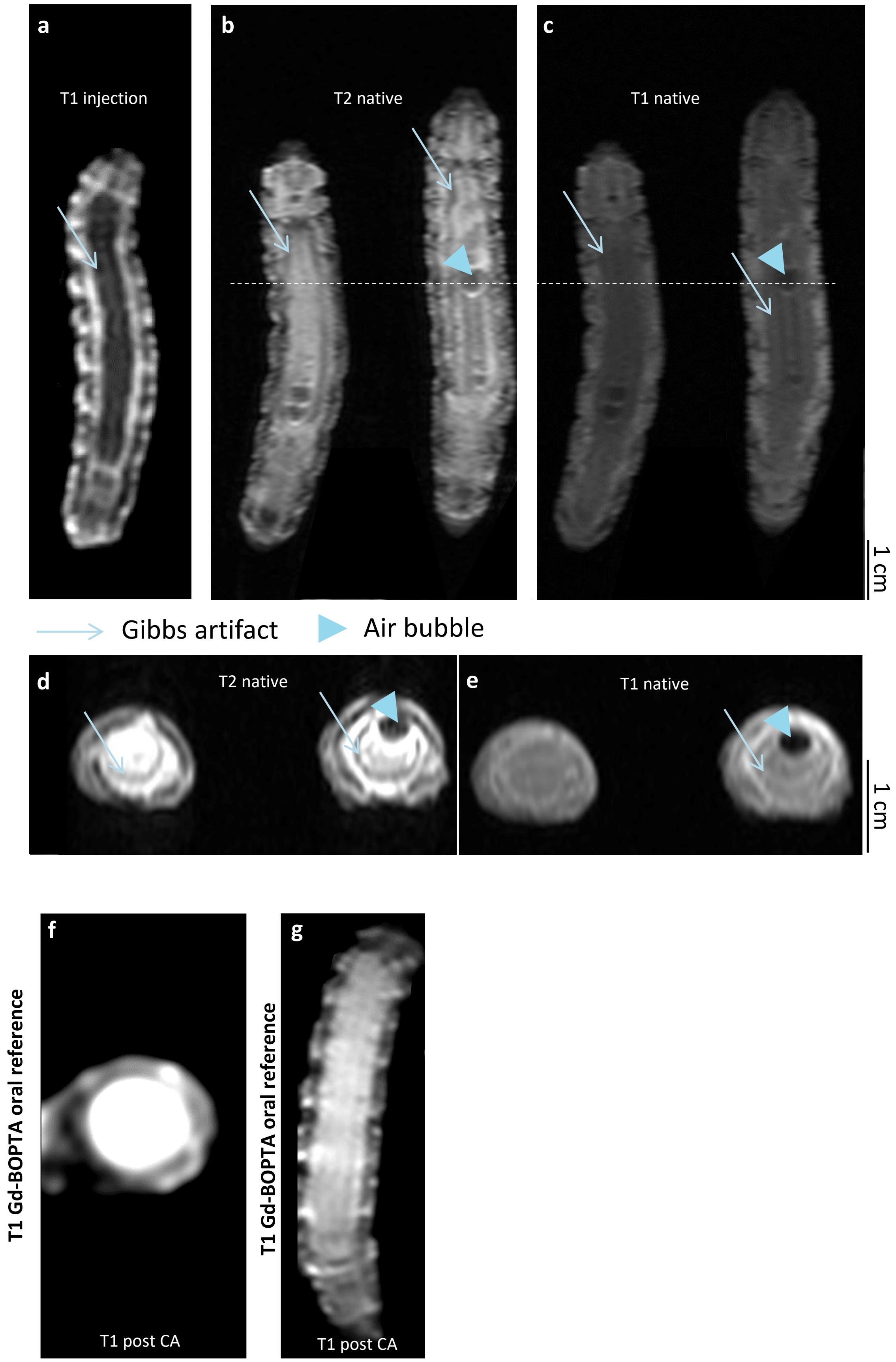

Fig. S10: Gibbs artifacts in (macro) MR imaging
T1 (with CA) and T2 weighted images of *M. sexta* larvae with (**a**) and without (**b**–**e**) Gd-BOPTA in the axial and coronal plane. Blue arrows indicate Gibbs artifacts, while black arrows indicate air bubbles in the alimentary tract. Dashed lines indicate the location of the axial slices. For comparison, animals with an oral application of Gd-BOPTA are shown in **Fig. f**–**g**.

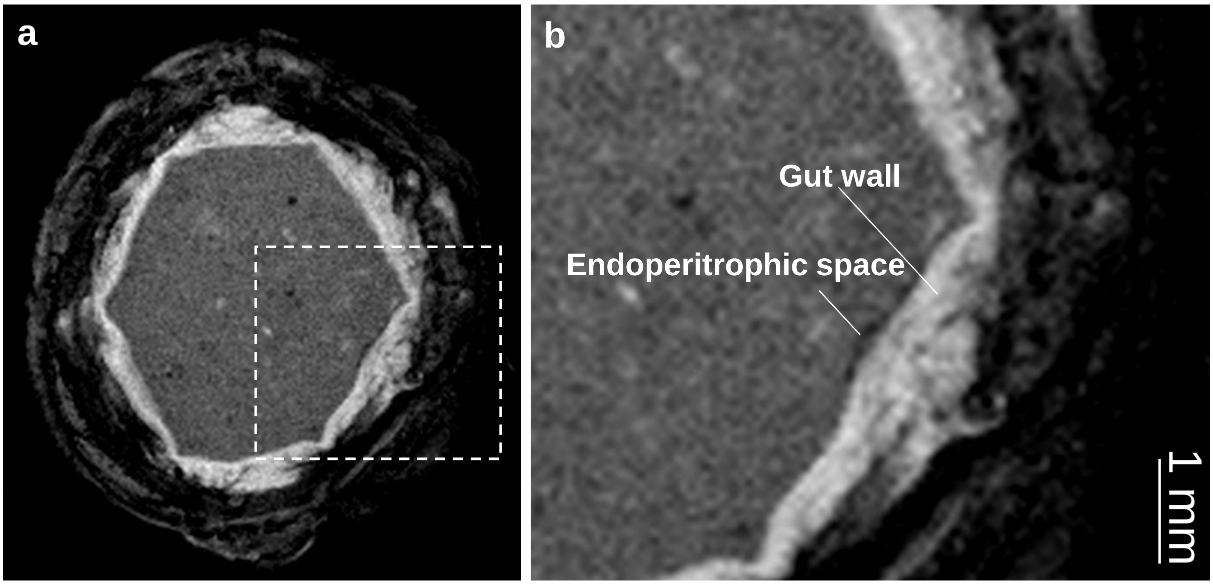

Fig. S11: Endoperitrophic space of *M. sexta* midgut
(**a**) Axial μ-MRI of a *M. sexta* L5 larvae. **(b)** Image section of (**a**): The endoperitrophic space is visible and has a maximal thickness of 0.105 mm.

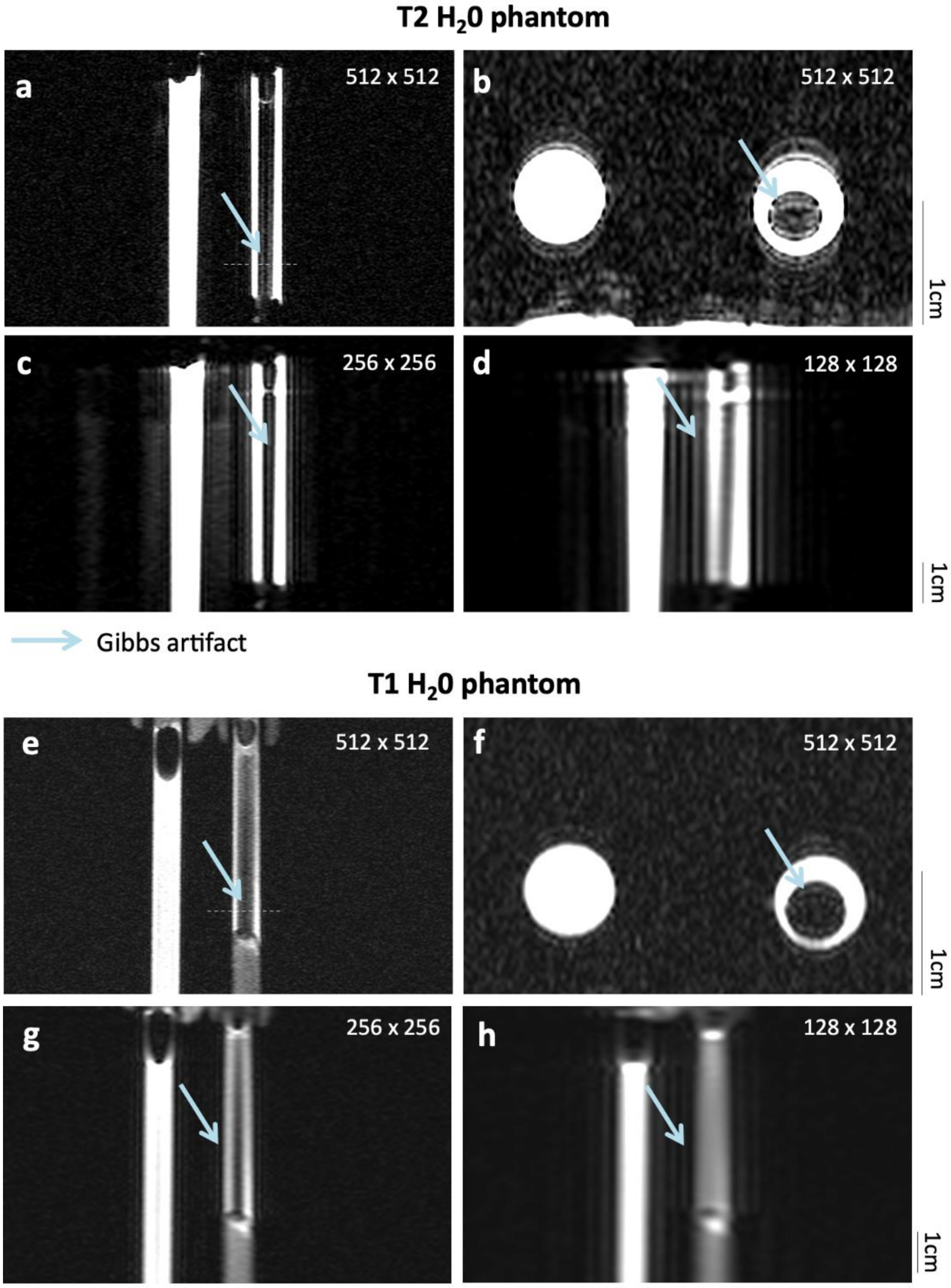

Fig. S12: Gibbs artifacts of phantoms in (macro) MR imaging
T2 (**a**–**d**) and T1 (**e**–**h**) weighted images of two phantoms with different matrix sizes. The matrix size is shown in the upper right corner of each figure. In each figure, a capillary filled with H_2O is on the left side, and two capillaries plugged into each other with H_2O filled into the interspace are shown on the right side. Gibbs artifacts (blue arrows) are most prominent on T2 weighted images with small matrix size but also present at 512 × 512 matrices (**a**–**b**).

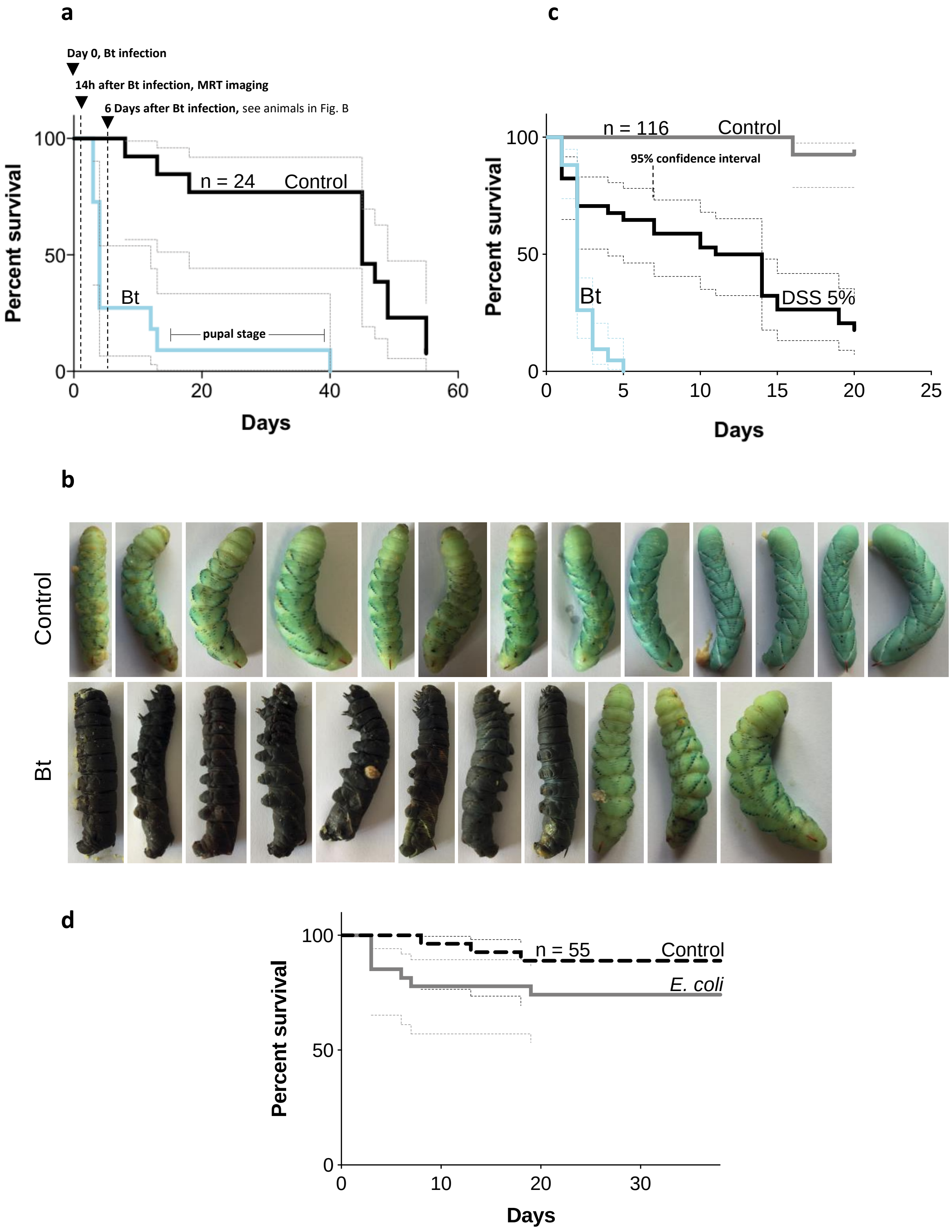

Fig. S13: Survival after Bt, DSS (5%) or *E. coli* treatment

(**a**) Large larvae (L5 d5/6) fed with normal diet (control n = 13) or with a diet containing Bt (n = 11) were observed for almost 60 days. Survival curves of *M. sexta* larvae (L5 d5/6) exposed to *B. thuringiensis* subjected to survival analyses using the Kaplan–Meier estimators' log-rank test with 95% confidence interval $p < 0.0001$). (**b**) Appearance of all untreated and infected larvae after six days of observation. (**c**) Small larva (L2 d0) were with normal diet (control n = 42), with a diet containing Bt (n = 40), or 5% DSS (n = 34) and were observed for 20 days. The survival curves of the 3 used treatments differd significantly from each other (Kaplan–Meier estimators' log-rank test with 95% confidence interval $p < 0.0001$)). (**d**) Kaplan Meier Survival curve of animals fed with normal diet (control n = 27), or with a diet containing *E. coli* (n = 28) with 95% confidence interval, starting at L5d5/6. Animals fed with *E. coli* showed no difference in survival compared to animals fed with a normal diet. Survival kinetics show the sum of the conducted experiments.

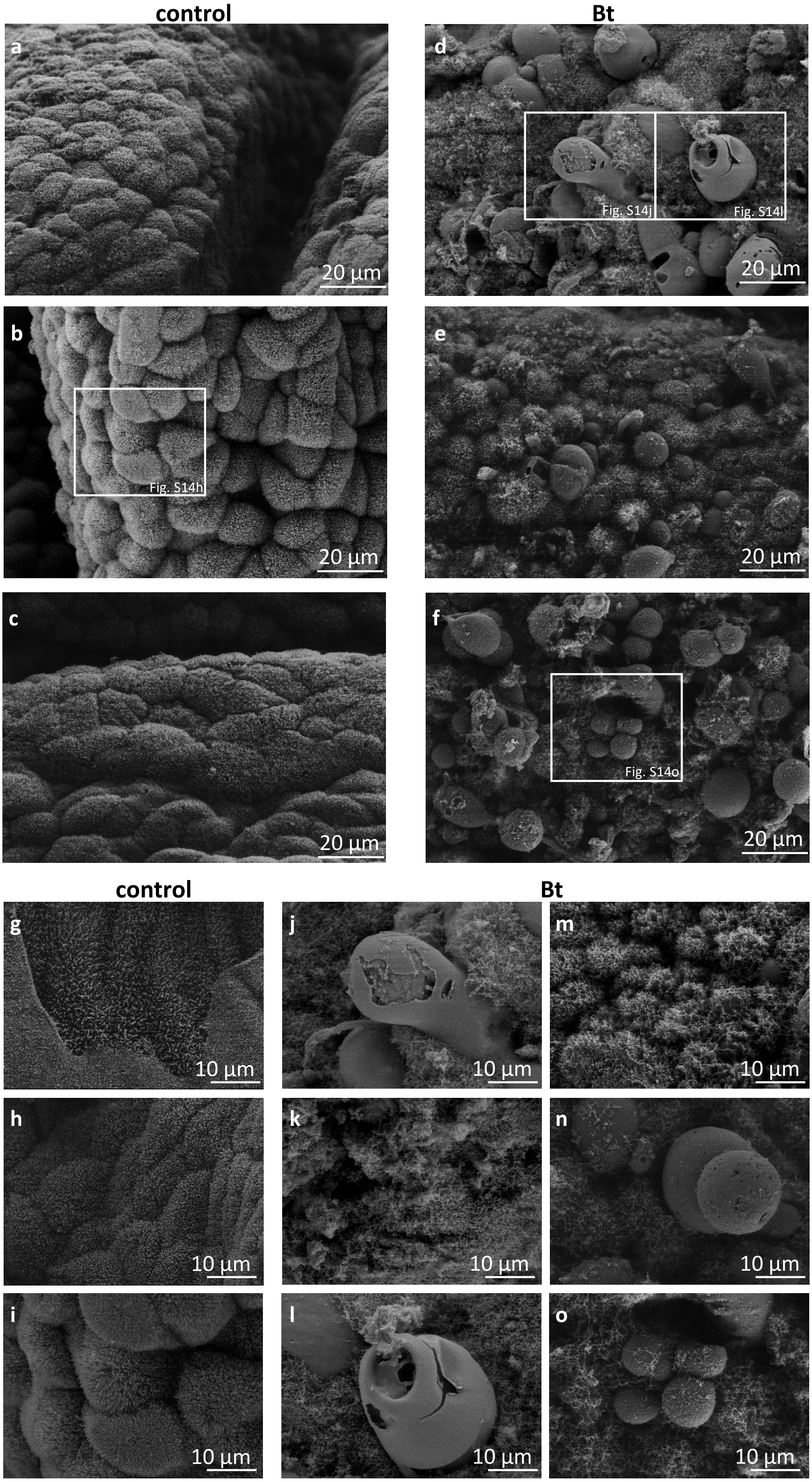

Fig. S14: Midgut ultrastructure after Bt treatment

Midgut ultrastructure (scanning electron microscopy) from control (**a**–**c**, **g**–**i,** n = 5) and Bt-treated (**d**–**f**, **j**–**o,** n = 3) larvae. Overviews are shown in **a**–**f** and details are given in **g**–**o**. 12 h Bt-exposed larvae showed loss of microvilli, cell swelling, and necrosis.

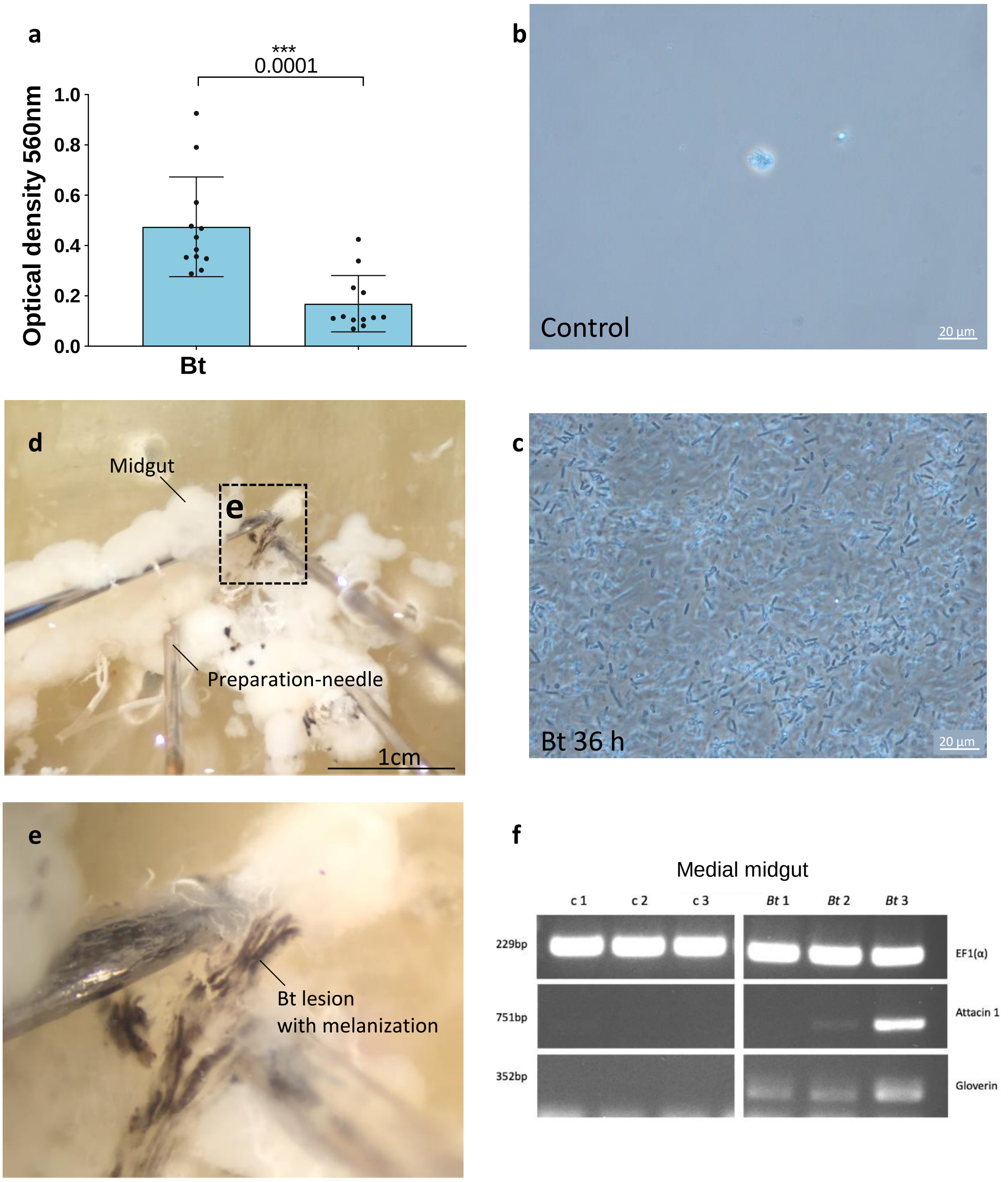

Fig. S15: Bacteria found in the hemolymph, melanization of the gut, and induction of AMPs after Bt treatment

Hemolymph samples from control (n = 12) and Bt treated animals (n = 12) 36 h after oral infection. The samples were incubated in standard I nutrient medium overnight. (**a**) The optical density (λ = 560 nm) of each sample was determined, indicating bacterial growth after gut perforation (n=24, two-tailed Mann Whitney test). (**b**) Phase-contrast microscopy image of a hemolymph sample from a control animal and (**c**) of a hemolymph sample from a Bt treated animal. Experiments were repeated independently: n = 4 with similar results . (**d**) Bt lesion with melanization in the midgut 24 h after infection (Bt n = 3, control = 3). (**e**) Detail of the midgut lesion. (**f**) Semiquantitative PCR analysis of the AMP genes attacin 1 and gloverin expression in the medial midgut of *M. sexta* larvae after Bt infection. Lane c 1–3 show cDNA samples from the medial midgut of control animals (n = 3), and lane Bt 1–3 show samples from animals treated with Bt (n = 3). *EF1(α)* was used as a control. Bar charts represent mean and SD. Every Data point represents a single animal. See the source data file or the end of the supplementary information file for a presentation of complete, uncropped gels. Source data are provided as a Source Data file.

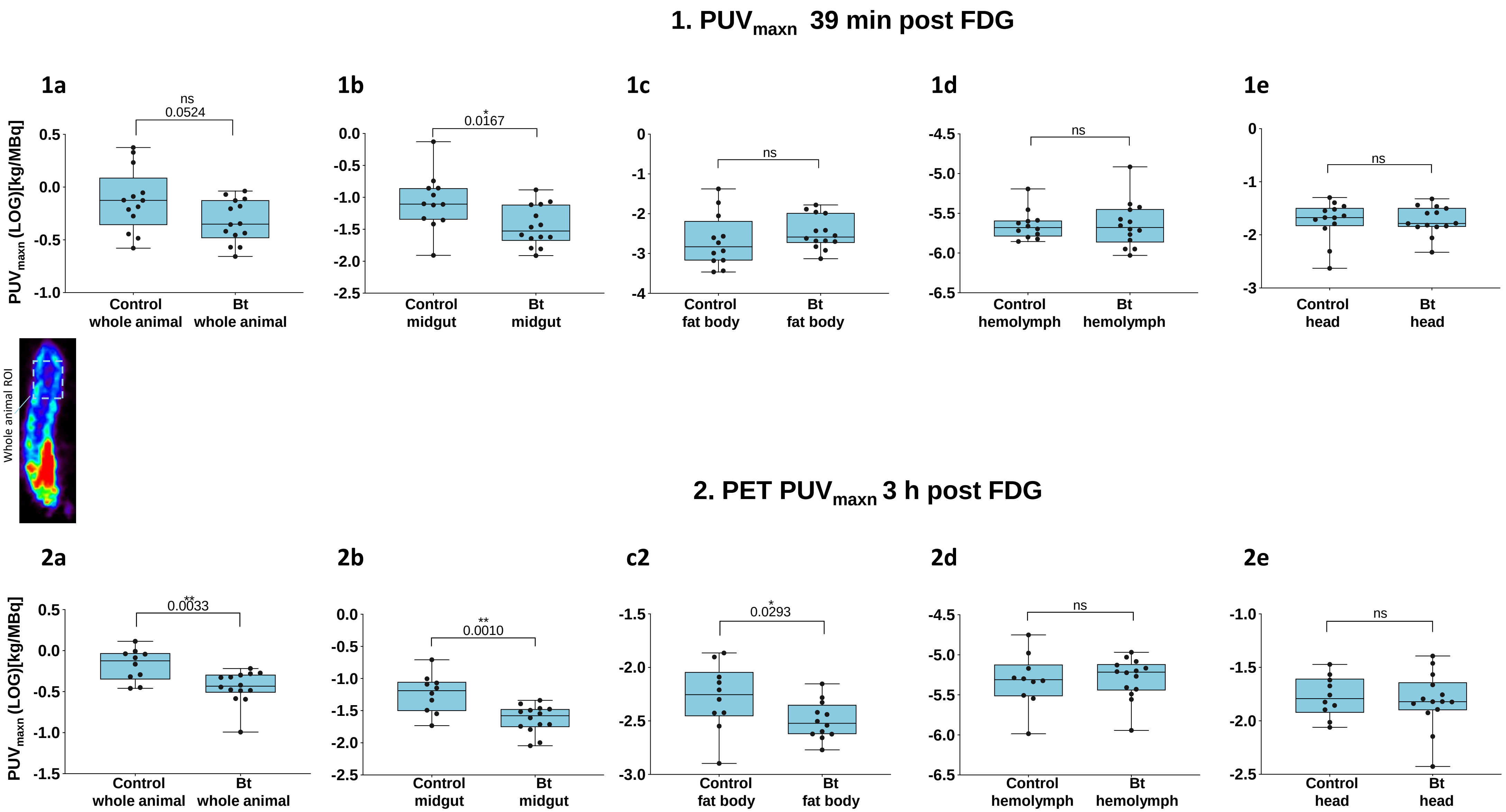

Fig. S16: Time and inflammation dependent organ-specific FDG uptake

PUV_{maxn} values of different tissues or parts of *M. sexta* after 39 min or 3 h post-FDG injection. Every Data point represents a single animal (1a: n=27 ,two-tailed t-test, 1b: n =26 ,two-tailed t-test, 1c: n =26 ,two-tailed t-test, 1d: n =26 ,two-tailed t-test, 1e: n = 27, two-tailed Mann Whitney test and 2a: n=24 ,two-tailed t-test, 2b: n =24 ,two-tailed t-test, 2c: n =22 ,two-tailed t-test, 2d: n =24 ,two-tailed t-test, 2e: n =24 ,two-tailed t-test, no adjustments were made). Box plots represent 25th-75th percentiles; whiskers represent min-max (show all points), and the centers represents median values. Source data are provided as a Source Data file.

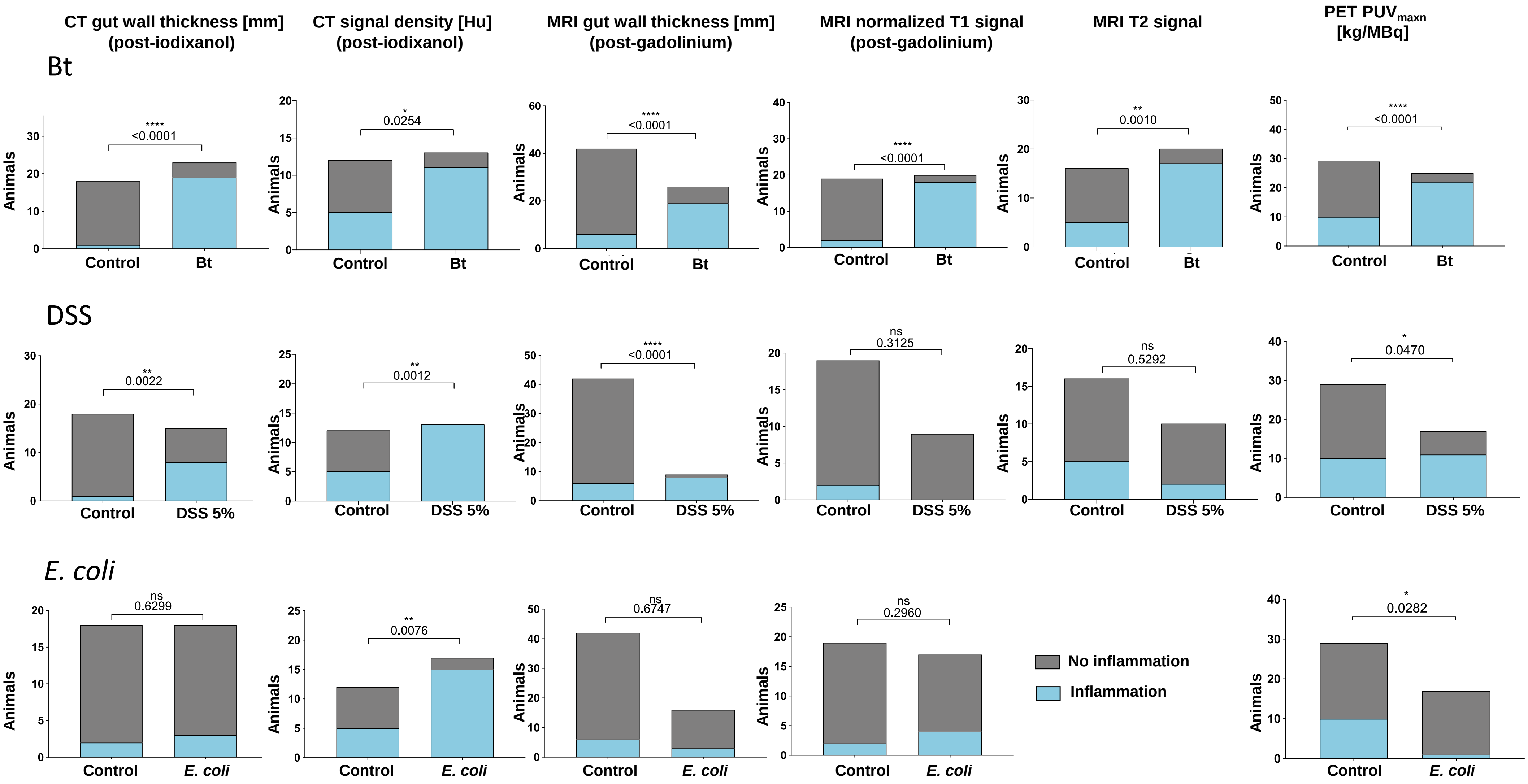

Fig. S17: Chi-square tests with threshold values for each diagnostic feature from the ROC analysis for Bt, DSS, or *E. coli* treatment

Animals fed with *E. coli*, DSS, and Bt were compared using the respective threshold values for each diagnostic feature from the ROC analysis. Source data are provided as a Source Data file.

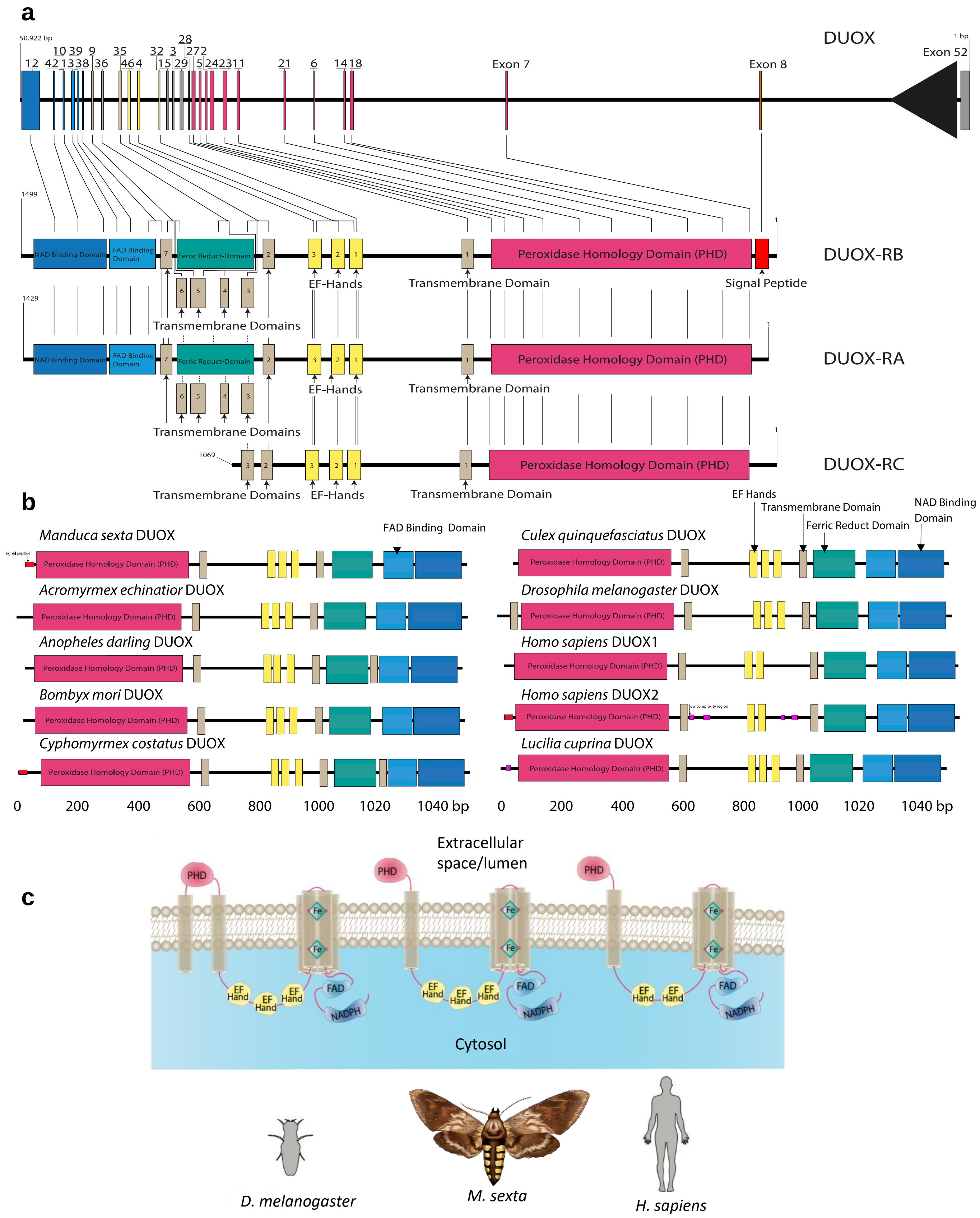

Fig. S18: DUOX gene and protein domain organization

(**a**) The *DUOX* gene is 50.922 bp long and contains 29 exons. 11 exons are coding for the PHD domain; three exons are coding for three EF-hand motifs; three exons are coding for one FAD binding domain; three exons are coding for one NAD binding domain; 5 exons are coding for seven transmembrane domains. Three different mRNAs are known: DUOX-RB (full-length DUOX transcript), DUOX-RA (similar to DUOX-RB but without the signal peptide), and DUOX RC (containing only the PHD domain and the EF-hand motifs). (**b**) DUOX domain organization from different species analyzed with SMART. Overlapping domains are not shown. Genbank accession numbers are: DUOX *Acromyrmex echinatior* = EGI68387.1, DUOX *Anopheles darling* = ETN58940.1, DUOX *Bombyx mori* = AFV61649.1, DUOX *Cyphomyrmex costatus* = KYN07485.1, DUOX *Culex quinquefasciatus* = XP_001844503.1, DUOX *Drosophila melanogaster* = FBpp0289611 (Fly Base), DUOX 1 *Homo sapiens* = AAI14939.1, DUOX 2 *Homo sapiens* = EAW77288.1 and DUOX *Lucilia cuprina* = KNC33589.1. The numbers indicate base pairs (bp). (**c**) Comparison of DUOX domain organization (and orientation) of *D. melanogaster*, *M. sexta*, and *H. sapiens*.

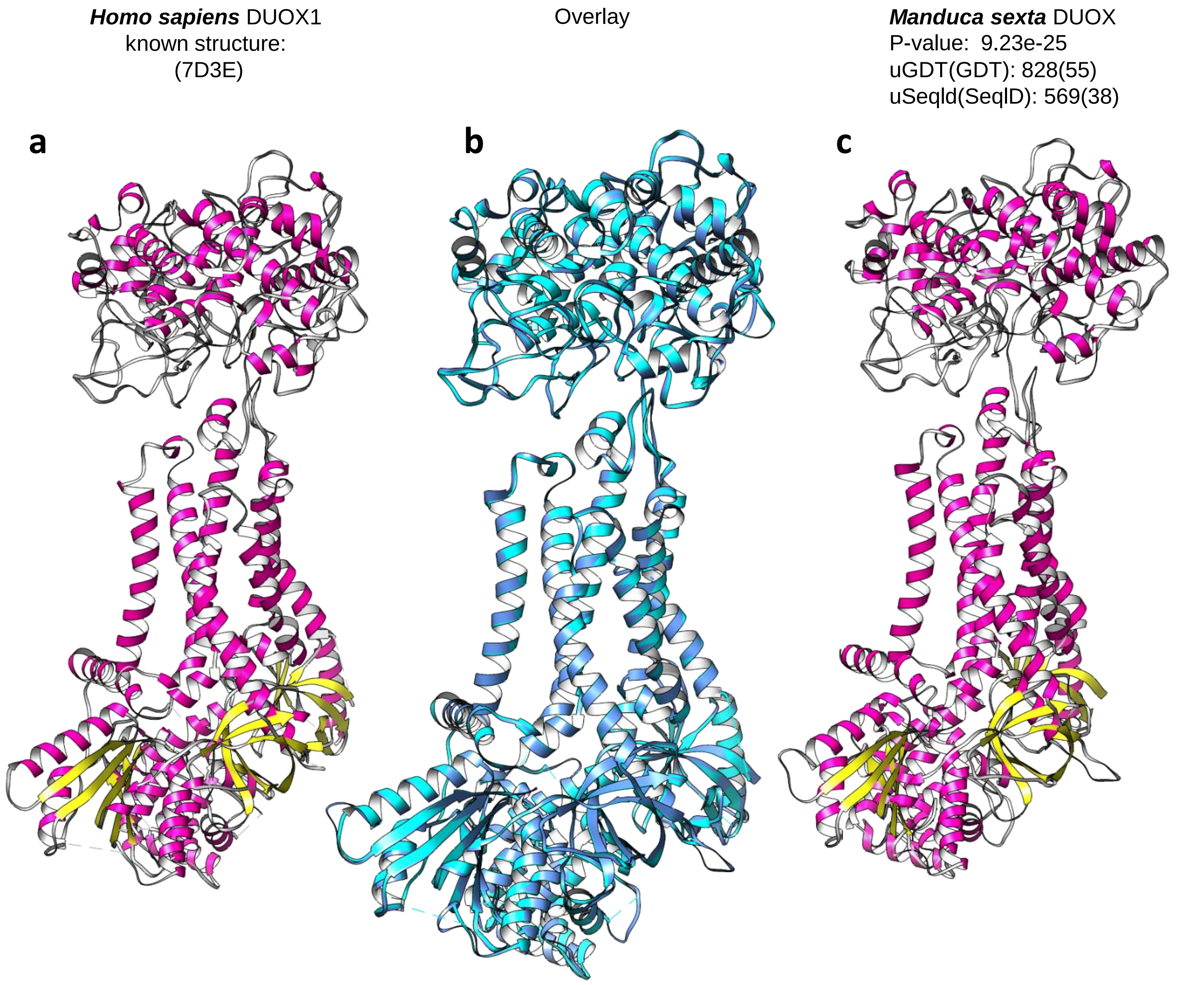

Fig. S19: Template-based protein structure modeling of *M. sexta* DUOX

(**a**) Known structure of Human DUOX 1. (**b**) Overlay of human DUOX and the predicted structure of *M. sexta* DUOX. (**c**) Predicted structure of *M. sexta* DUOX.

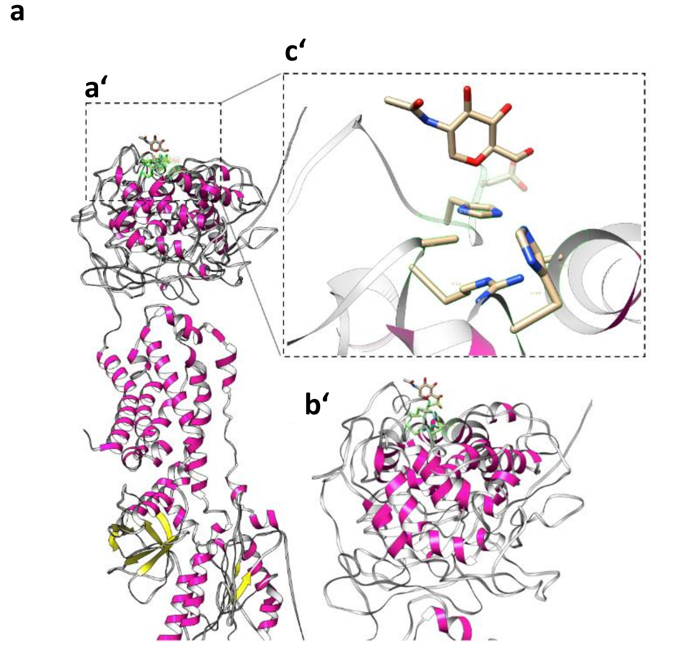

Fig. S20a: N-acetyl-beta-D-glucosamine binding site prediction (pocket 1) of *M. sexta* DUOX PHD
N-acetyl-beta-D-glucosamine binding site prediction of *M. sexta* DUOX PHD with model-assisted protein binding site prediction with different magnifications (**a'**–**c'**). Pocket: 1, Multiplicity: 166, Ligand: NAG, Binding residues: A97 R99 A301 H489 D552 H555. Details are given in **Table S8**.

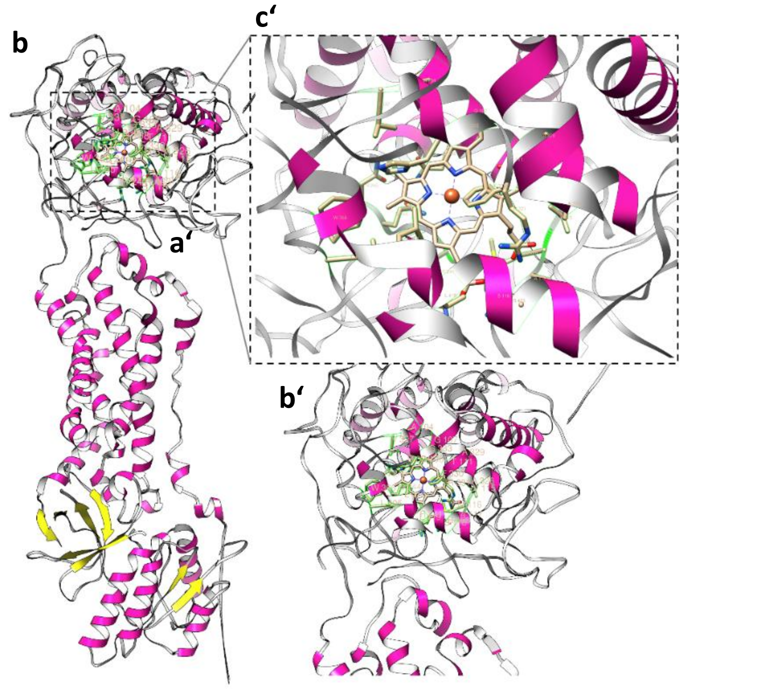

Fig. S20b: Heme or Protoporphyrin IX containing Fe binding site prediction (pocket 2) of *M. sexta* DUOX PHD
Heme or Protoporphyrin IX containing Fe binding site prediction of *M. sexta* DUOX PHD with model-assisted protein binding site prediction with different magnifications (**a'**–**c'**). Pocket: 2, Multiplicity: 165, Ligand: HEM, Binding residues: A104 G107 Q108 T111 V115 M116 A117 S118 R241 Q244 N245 A326 A329 F330 R331 G333 H334 V337 W366 L405 L416 L419 R423. Details are given in **Table S8**.

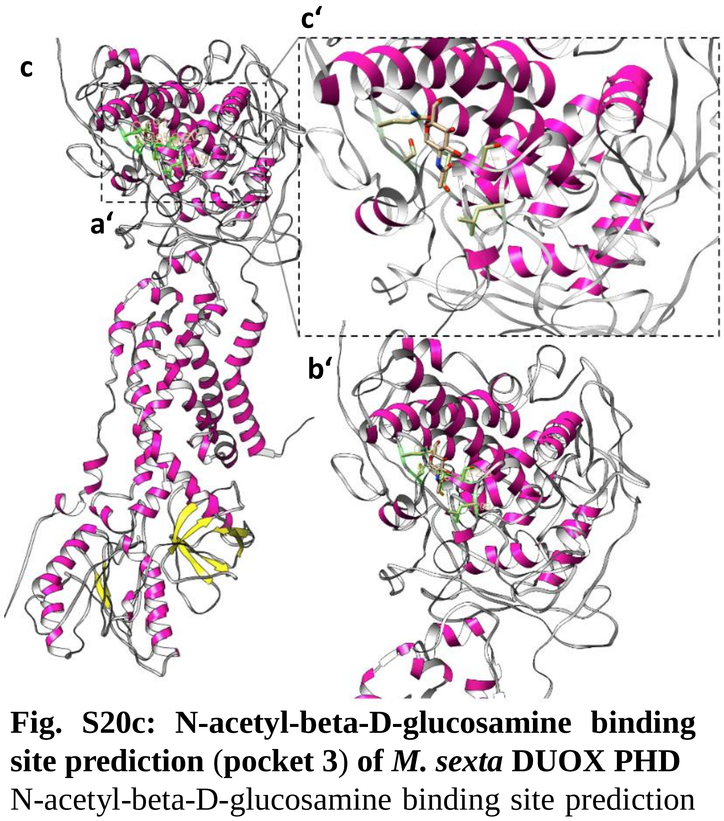

Fig. S20c: N-acetyl-beta-D-glucosamine binding site prediction (pocket 3) of *M. sexta* DUOX PHD
N-acetyl-beta-D-glucosamine binding site prediction of *M. sexta* DUOX PHD with model-assisted protein binding site prediction with different magnifications (**a'**–**c'**). Pocket: 3, Multiplicity: 118, Ligand: NAG, Binding residues: S195 Q197 S202 S203 L209. Details are given in **Table S8**.

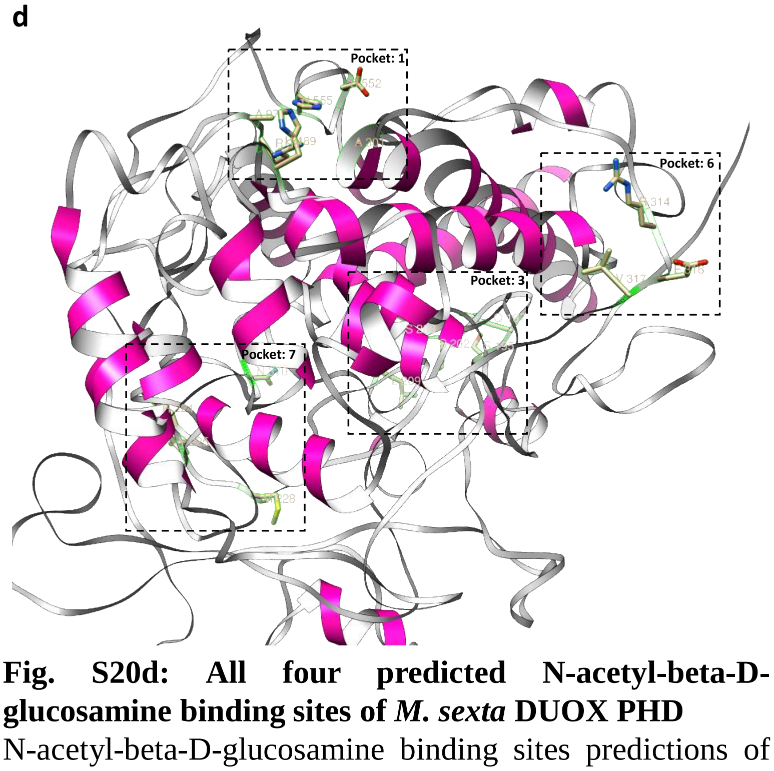

Fig. S20d: All four predicted N-acetyl-beta-D-glucosamine binding sites of *M. sexta* DUOX PHD
N-acetyl-beta-D-glucosamine binding sites predictions of *M. sexta* DUOX PHD with model-assisted protein binding site prediction. **Pocket: 1**, Multiplicity: 166, Ligand: NAG, Binding residues: A97 R99 A301 H489 D552 H555; **Pocket: 3**, Multiplicity: 118, Ligand: NAG, Binding residues: S195 Q197 S202 S203 L209; **Pocket: 6**, Multiplicity: 80, Ligand: NAG, Binding residues: R314 E316 V317; **Pocket: 7**, Multiplicity: 79, Ligand: NAG, Binding residues: H224 Y225 M228 N370. Details are given in **Table S8**.

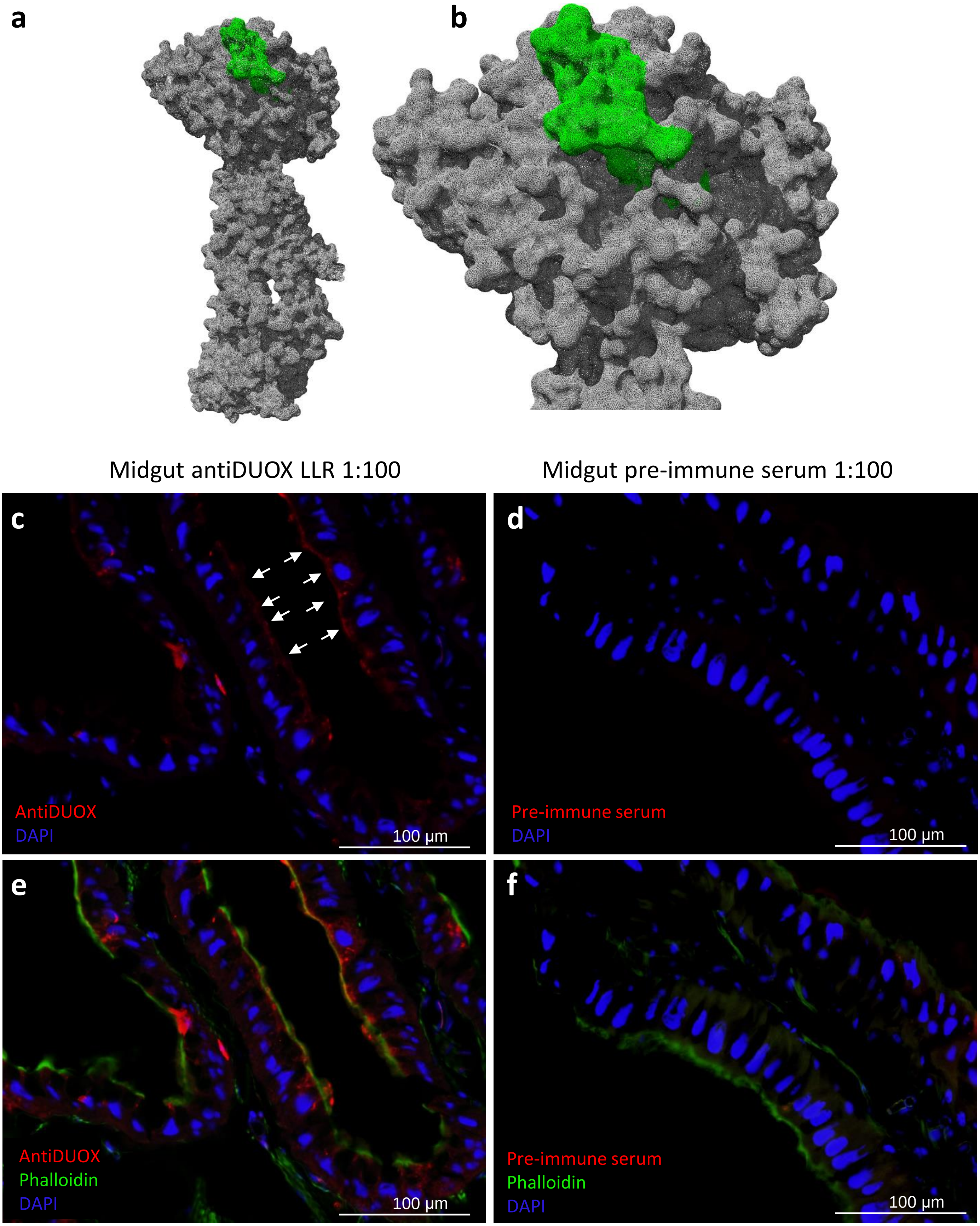

Fig. S21: Epitope of the generated antiDUOX LLR antibody and immunohistochemical identification of DUOX in midgut of *M. sexta* larvae

(**a**–**b**) The surface structure of DUOX and the peroxidase homology domain (PHD) with protein mesh surface. The epitope of the polyclonal anti-DUOX antibody (amino acids 342–361) is shown in green. Cryosections incubated with affinity-purified anti-DUOX-LRR-antibody (red, **c** and **f**) and with pre-immune serum (red, **d** and **f**). Further labelling of nuclei with DAPI (blue) and of F-actin of microvilli and muscles with FITC-Phalloidin (green). Experiments were repeated independently: n = 8 with similar results.

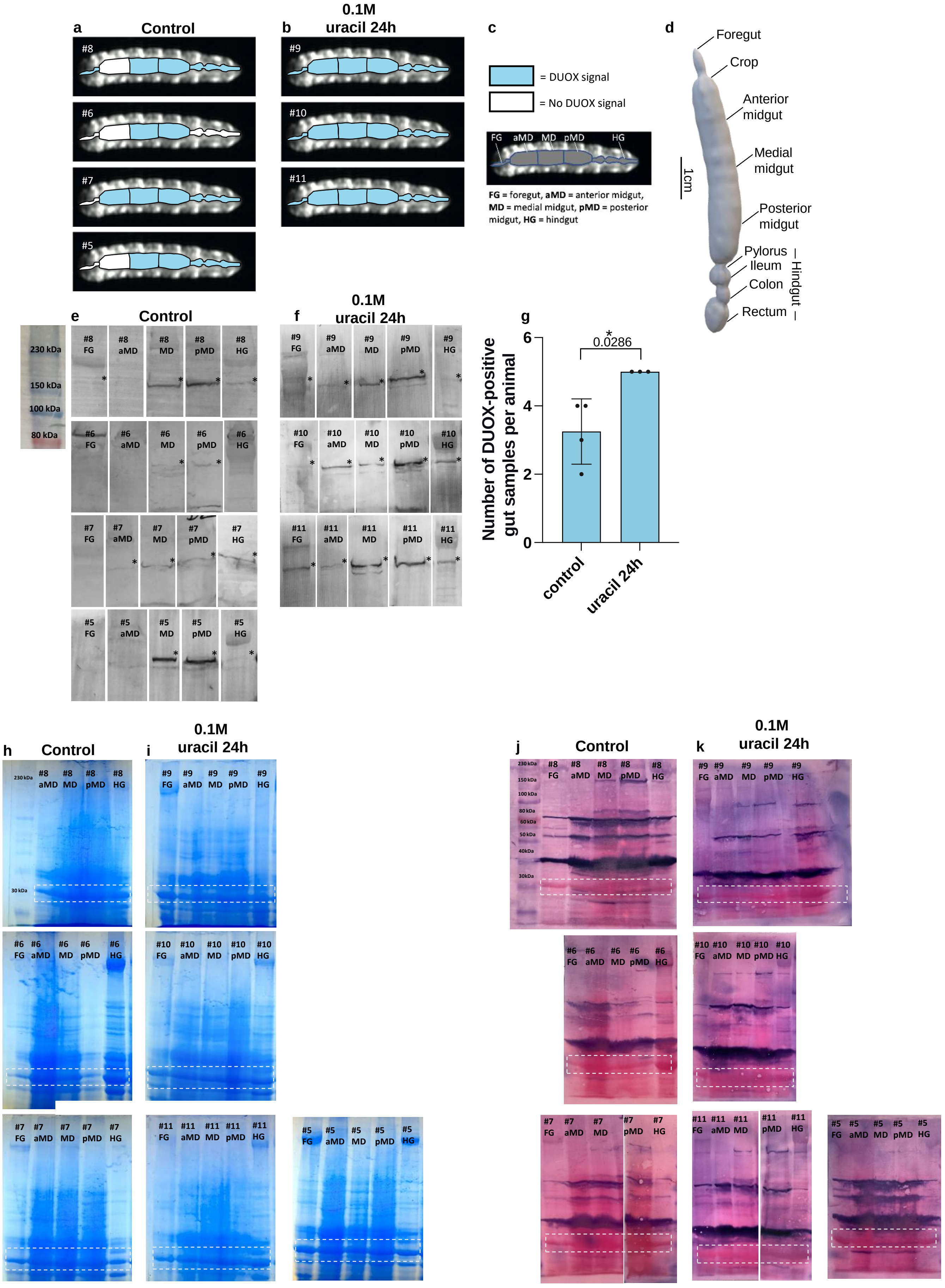

Fig. S22: Western blot with the anti-*M. sexta*-DUOX antibody of different gut regions
(**a–c**) Illustrations of the presence of *M. sexta* DUOX based on the Western blots E–F. (**d**) The anatomy of the digestive system of *M. sexta* is given as a reference. (**e–f**) Western blot analysis with anti-DUOX LLR of the digestive systems of *M. sexta* 24 h after uracil treatment. FG: foregut, aMD: anterior midgut, MD: medial midgut, pMD: posterior midgut and the hindgut (HG). The hindgut (HG) was used as a control (always right lanes, not shown). * indicates a protein with the rel. molecular weight of 170 kDa (DUOX). See the source data file for a presentation of complete, uncropped blots. (**g**) The number of DUOX-positive samples was significantly higher in animals exposed to uracil (control: n = 4, uracil: n = 3), two-tailed Mann Whitney test, P = 0.0286, no adjustments. The bar chart represents the mean and SD. (**h–i**) Coomassie gels of the used samples. (**j–k**) Staining of the western blots with Ponceau S. A protein with a rel. molecular weight of 30 kDa was used as a loading control (dashed lines). The # identifies every animal in this experiment and allows comparison of SDS-PAGE and Western blot results. See the source data file for a presentation of complete, uncropped blots and gels.

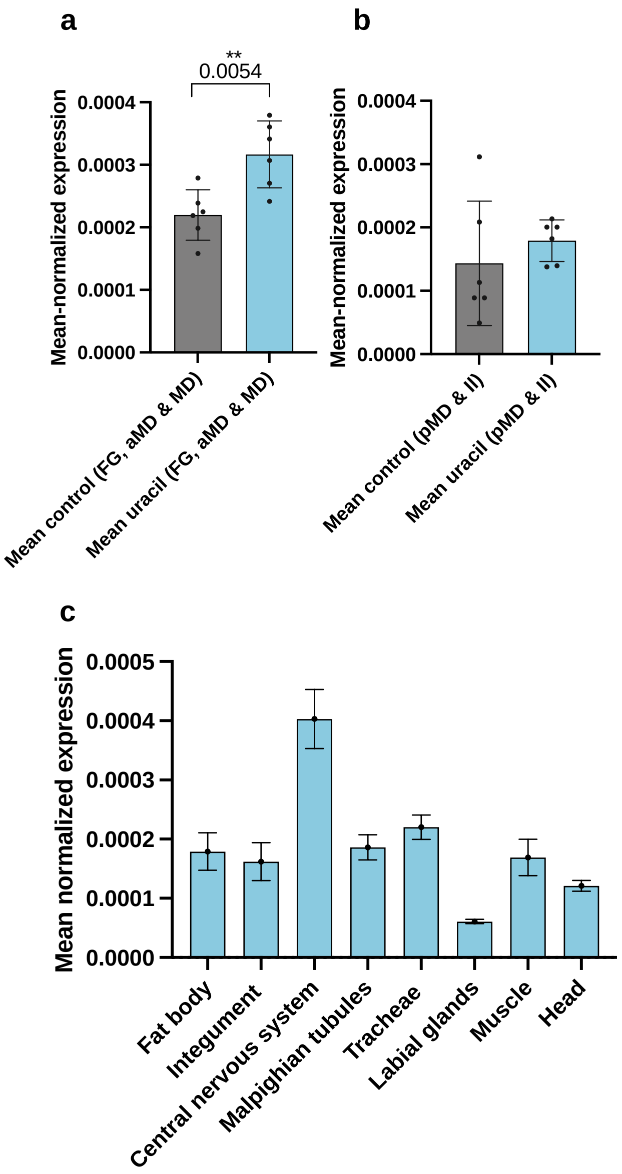

Fig. S23: Quantitative RT-PCR analysis of *MsDUOX* in the gut and different tissues
(**a**) Mean-normalized *MsDUOX* expression values of the anterior digestive tract (FG, Foregut; aMD, anterior midgut; and MD, medial midgut) or the posterior digestive tract (pMD, posterior midgut and Il, ileum, part of the hindgut), n = 12, two-tailed t-test, P = 0.0054, no adjustments (**b**). (**c**) Mean-normalized *DUOX* expression values of different tissues (n = 1; 3-4 animals were pooled for each tissue). Bar charts represent mean and SD. Source Data are provided as a Source Data file.

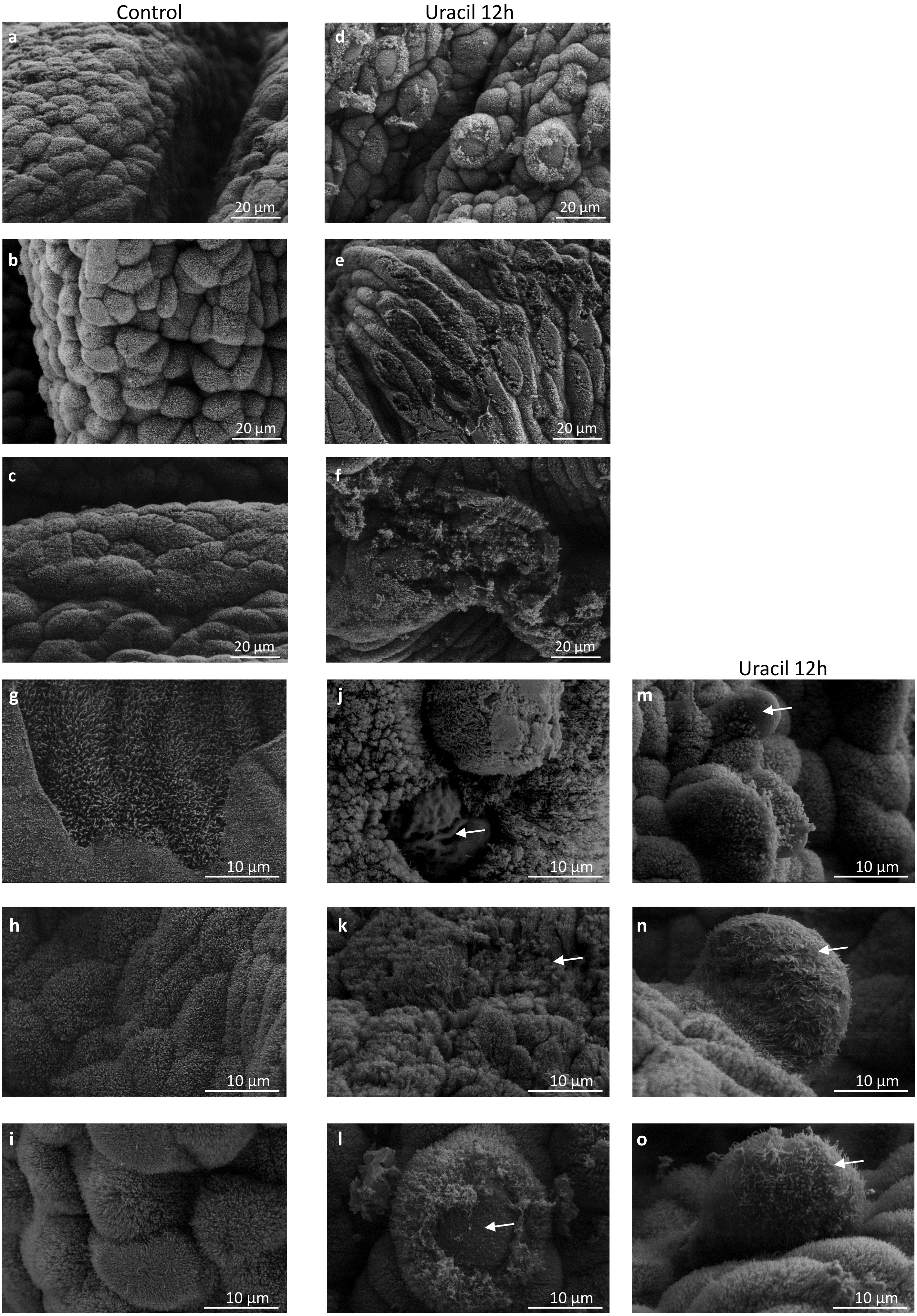

Fig. S24: Midgut enterocytes in scanning electron microscopy (SEM) after 0.1 M uracil treatment SEM revealed loss of microvilli and cell swelling after uracil treatment. (**a–c**) and (**g–i**) SEM images from control midguts (n = 5), (**d–o**) SEM images from uracil (n = 6) treated animals.

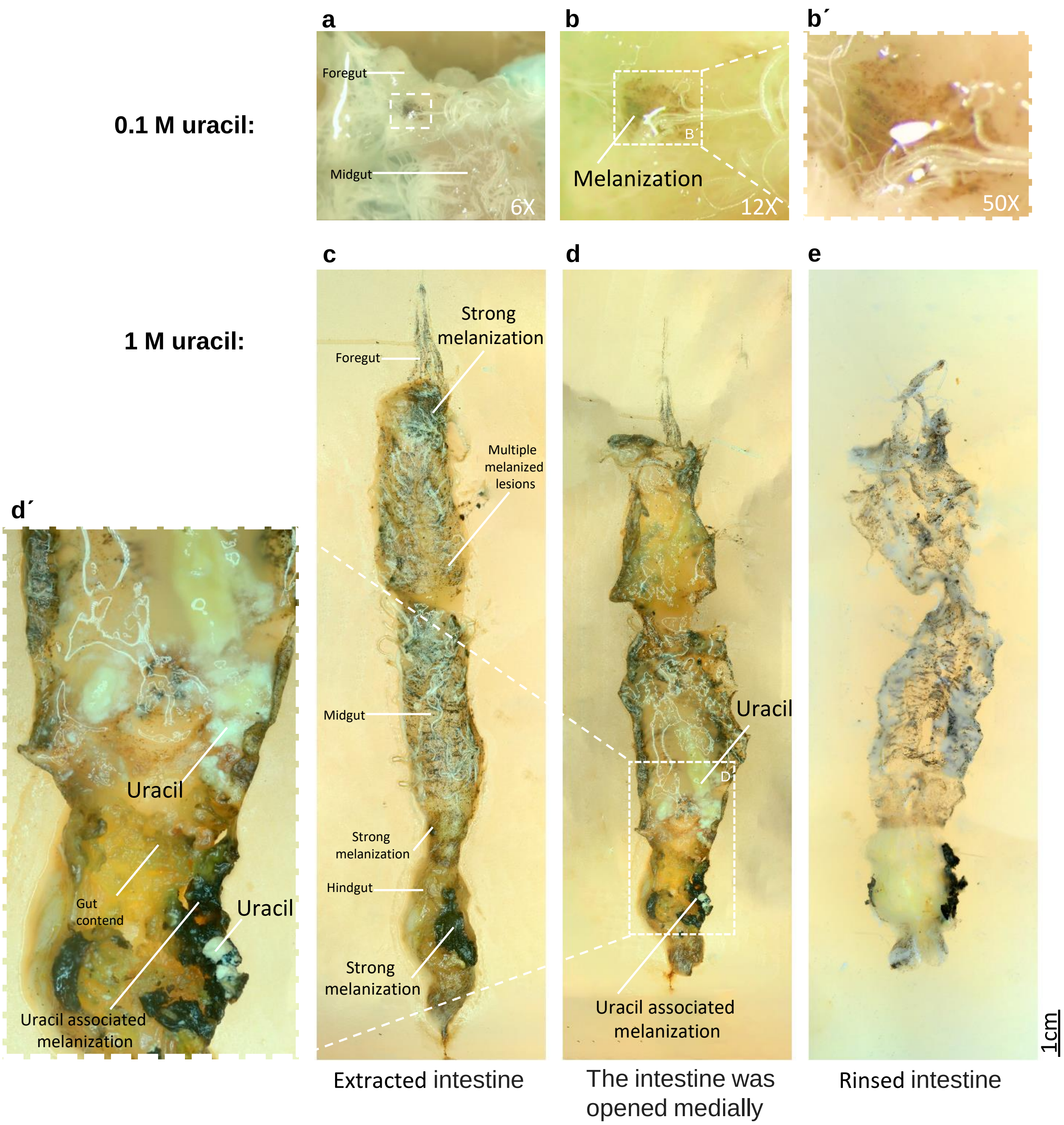

Fig. S25: Uracil concentration-dependent melanized lesions of the digestive tract
(**a**–**b**) After 0.1 M uracil treatment, only small melanized lesions were found after 5 days of treatment. However, after 5 days of 1 M uracil treatment, large melanized lesions were found all over the digestive tract (**c**–**e**). Note the strong melanization at the hindgut with uracil deposits at its center (**c**, **d**, and **d**´).

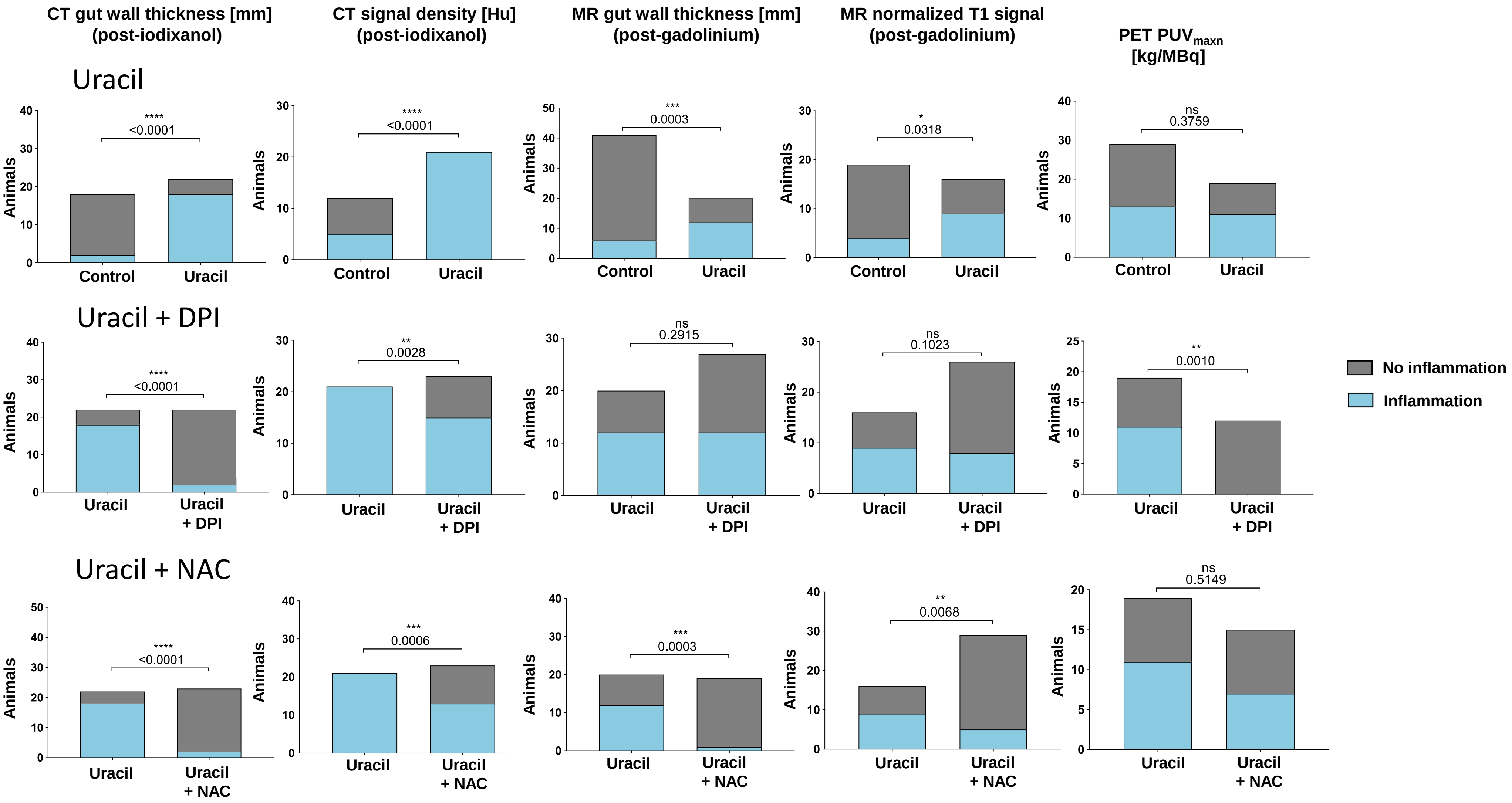

Fig. S26: Chi-square tests with threshold values for each diagnostic feature from the ROC analysis for uracil treatment (+ inhibitor or scavenger)
Animals fed with uracil, uracil + NAC, uracil + DPI, or control diet were compared using the respective threshold values for each diagnostic feature from the ROC analysis. Chi-square tests were calculated. Source data are provided as a Source Data file.

uncropped scans of gels from Fig. S15

a

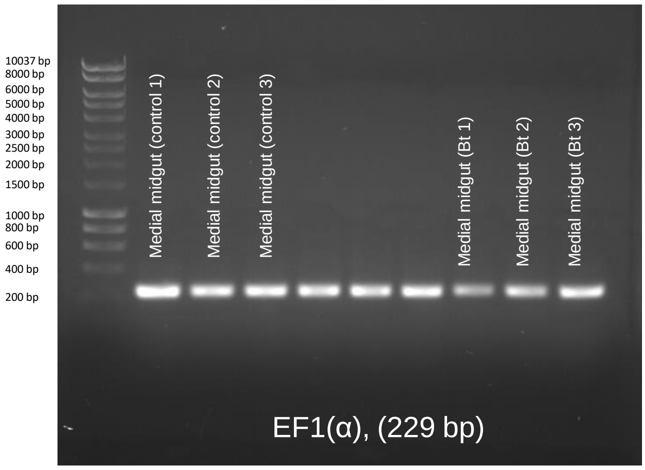

b

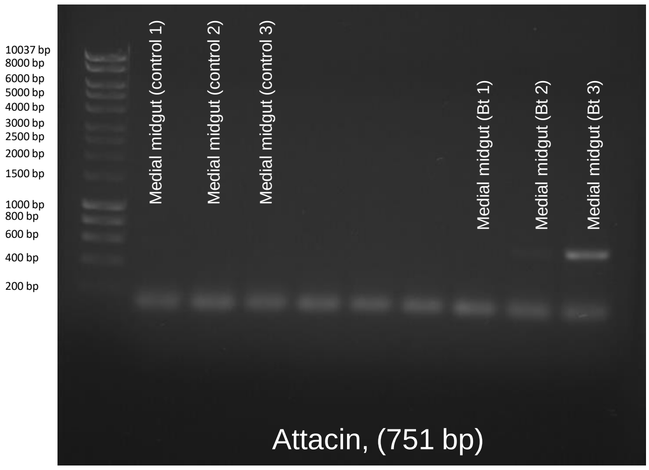

c

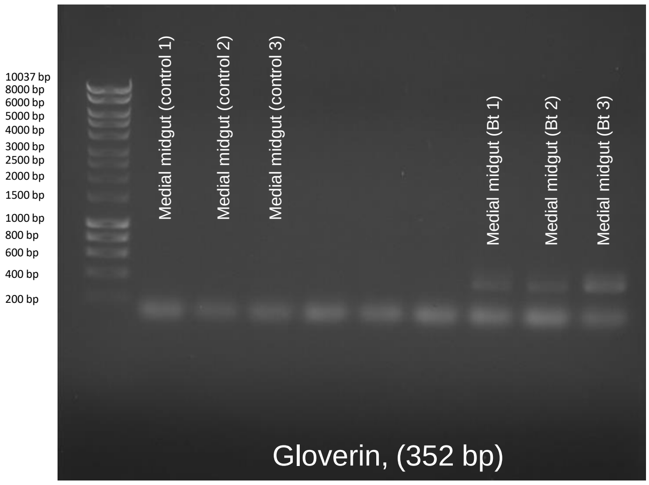

Supplement: Supplementary file 1 — Supplementary Information [file 41467_2022_34865_MOESM1_ESM.pdf]
