# Supplementary material for: High-throughput screening of caterpillars as a platform to study host–microbe interactions and enteric immunity
Source: Nat Commun. 2022 Nov 24;13:7216. doi: 10.1038/s41467-022-34865-7 (PMC9700799; doi:10.1038/s41467-022-34865-7)
Supplement: Supplementary file 3 — Description of Additional Supplementary Files [file 41467_2022_34865_MOESM3_ESM.docx]

**Description of Additional Supplementary Files**

File Name: Supplementary Video 1

Description: Mode of action for extracellular CAs in CT or MRI

File Name: Supplementary Video 2

Description: Mode of action for FDG-PET

File Name: Supplementary Video 3

Description: µMRI cine loops of the heart (dorsal vessel) of *Manduca sexta* in sagittal (**a-c**) and axial (**d**) orientation

File Name: Supplementary Video 4

Description: µMRI cine loops of *Manduca sexta* before (a) and after CA application (**b-d**) showing the distribution of the CA over time

File Name: Supplementary Video 5

Description: 3D volume renderings of contrast-enhanced ex vivo µCT midgut regions of *M. sexta*

File Name: Supplementary Video 6

Description: Optoacoustic reconstructions of *M. sexta* with (**a**) and without (**b**) oral contrast

File Name: Supplementary Video 7

Description: Structure alignment of insect and mammalian DUOX peroxidase homology domains (PHDs) based on homology modeling with DUOX1 from *Mus musculus* as template. (*Manduca sexta* = pink, *Drosophila melanogaster* = blue, *Tribolium castaneum* = violet, *Homo sapiens* (DUOX1) = yellow, *Homo sapiens* (DUOX2) = orange, *Mus musculus* (DUOX1) = green)

File Name: Supplementary Video 8

Description: Homology modeling of *M. sexta DUOX*. The known structure of *H. sapiens DUOX*1 (**left**) and model-assisted protein binding site prediction of *M. sexta* DUOX (**right**, shown in green)

File Name: Supplementary Video 9

Description: Heme or Protoporphyrin IX containing Fe binding site prediction of *M. sexta* DUOX PHD with model-assisted protein binding site prediction
