# Supplementary material for: High-throughput screening of caterpillars as a platform to study host–microbe interactions and enteric immunity
Source: Nat Commun. 2022 Nov 24;13:7216. doi: 10.1038/s41467-022-34865-7 (PMC9700799; doi:10.1038/s41467-022-34865-7)
Supplement: Supplementary file 13 — Reporting Summary [file 41467_2022_34865_MOESM13_ESM.pdf]

nature portfolio

Corresponding author(s): Ulrich Flögel

Last updated by author(s): 2022/11/07

Reporting Summary

Nature Portfolio wishes to improve the reproducibility of the work that we publish. This form provides structure for consistency and transparency in reporting. For further information on Nature Portfolio policies, see our Editorial Policies and the Editorial Policy Checklist.

Statistics

For all statistical analyses, confirm that the following items are present in the figure legend, table legend, main text, or Methods section.

n/a	Confirmed	
☐	☒	The exact sample size (*n*) for each experimental group/condition, given as a discrete number and unit of measurement
☐	☒	A statement on whether measurements were taken from distinct samples or whether the same sample was measured repeatedly
☐	☒	The statistical test(s) used AND whether they are one- or two-sided<br>*Only common tests should be described solely by name; describe more complex techniques in the Methods section.*
☒	☐	A description of all covariates tested
☐	☒	A description of any assumptions or corrections, such as tests of normality and adjustment for multiple comparisons
☐	☒	A full description of the statistical parameters including central tendency (e.g. means) or other basic estimates (e.g. regression coefficient) AND variation (e.g. standard deviation) or associated estimates of uncertainty (e.g. confidence intervals)
☐	☒	For null hypothesis testing, the test statistic (e.g. *F*, *t*, *r*) with confidence intervals, effect sizes, degrees of freedom and *P* value noted<br>*Give P values as exact values whenever suitable.*
☒	☐	For Bayesian analysis, information on the choice of priors and Markov chain Monte Carlo settings
☒	☐	For hierarchical and complex designs, identification of the appropriate level for tests and full reporting of outcomes
☐	☒	Estimates of effect sizes (e.g. Cohen's *d*, Pearson's *r*), indicating how they were calculated

Our web collection on statistics for biologists contains articles on many of the points above.

Software and code

Policy information about availability of computer code

Data collection	Horos 3.3.5 - 4.0, MIM Viewer PEM b1.2.4, ViewMSOT, CTAn 1.20.8.0, NRecon 1.7.4.6, Slicer v4.8.1 r26813, LAS X 3.5.1, OpenCFU 2.5 and ParaVision 5.1
Data analysis	Graphpad Prism 8, Statistica v12.5.192.7,Matlab, 2020a, Fiji ImageJ v2.1.0, Cell Counter (2010/12/07), Raptor X (2021/06), UCSF Chimera v1.11.2., SMART 9.0, SignalP 4.0., OpenCFU-2.5, Image Studio v5.2.5, OriginPro 2020b and Analyze 14.0.

For manuscripts utilizing custom algorithms or software that are central to the research but not yet described in published literature, software must be made available to editors and reviewers. We strongly encourage code deposition in a community repository (e.g. GitHub). See the Nature Portfolio guidelines for submitting code & software for further information.

Data

Policy information about availability of data

All manuscripts must include a data availability statement. This statement should provide the following information, where applicable:

- Accession codes, unique identifiers, or web links for publicly available datasets
- A description of any restrictions on data availability
- For clinical datasets or third party data, please ensure that the statement adheres to our policy

All data supporting the findings of this study are available within the article and its supplementary material. Raw imaging data are available from the corresponding authors. Source data are provided with this paper. All DNA sequencing data was uploaded to GenBank sequence database (OP630947-OP630951).

Field-specific reporting

Please select the one below that is the best fit for your research. If you are not sure, read the appropriate sections before making your selection.

[x] Life sciences [ ] Behavioural & social sciences [ ] Ecological, evolutionary & environmental sciences

For a reference copy of the document with all sections, see nature.com/documents/nr-reporting-summary-flat.pdf

Life sciences study design

All studies must disclose on these points even when the disclosure is negative.

Sample size	No statistical methods were used to predetermine sample size. The sample sizes were based on applicable standards and routinely employed sample sizes in the field. A detailed overview of the sample size is given in Figure 10.
Data exclusions	808 animals were analyzed in MRI, CT, and PET. Three outliers were excluded using the ROUT (Q = 1%) method to achieve normal distribution. A detailed overview of the sample size is given in Figure 10.
Replication	All attempts at replication were successful. Experiments without quantification were repeated at least four times.
Randomization	Larvae were chosen randomly from the colony (= allocation was random within the required developmental age margin). One strength of our high-throughput screening platform is that the experimental and control larvae are collected, treated, and tested simultaneously.
Blinding	Blinding was not feasible because the studied phenotypes allowed unambiguous identification of the treatment in most cases. Blinding was also unnecessary as GraphPad Prism was used for semi-automatic unbiased quantification of data based on experiments performed by the same investigator.

Reporting for specific materials, systems and methods

We require information from authors about some types of materials, experimental systems and methods used in many studies. Here, indicate whether each material, system or method listed is relevant to your study. If you are not sure if a list item applies to your research, read the appropriate section before selecting a response.

Materials & experimental systems

n/a	Involved in the study	
[ ]	[x]	Antibodies
[x]	[ ]	Eukaryotic cell lines
[x]	[ ]	Palaeontology and archaeology
[ ]	[x]	Animals and other organisms
[x]	[ ]	Human research participants
[x]	[ ]	Clinical data
[x]	[ ]	Dual use research of concern

Methods

n/a	Involved in the study	
[x]	[ ]	ChIP-seq
[x]	[ ]	Flow cytometry
[x]	[ ]	MRI-based neuroimaging

Antibodies

Antibodies used	We used the M. sexta plasmatocyte specific monoclonal antibody MS#13 (plasmatocyte specific beta-Integrin), the M. sexta granular cell specific monoclonal antibody MS#7, the oenocytoid labelling anti-M. sexta β 1,3-glucan recognition protein 1 immune serum, the M. sexta spherule cell labelling anti-Ephestia kuehniella haemolymph esterase immune serum and the custom-created polyclonal anti-M. sexta-DUOX antibody. These antibodies are made by the scientific community as described in Von Bredow, C. R., and T. E. Trenczek. "Distinguishing Manduca sexta haemocyte types by cytometric methods." Invertebrate Survival Journal (2022): 13-27. They are available upon personal request.<br><br>As secondary antibodies, we used:<br>-Anti-rabbit alkaline phosphatase (ALP)-conjugated secondary antibody (goat anti-rabbit IgG H&L, Roth #4751)<br>-The used Donkey Anti-Mouse IgG H&L (DyLight 549) is now replaced by The used Donkey Anti-Mouse IgG H&L (DyLight 550) (ab96876)<br>-Goat Anti-Rabbit IgG H&L (DyLight® 488)(ab96899)
Validation	The M.sexta plasmatocyte specific marker MS#13 (plasmatocyte specific beta-Integrin) is extensively validated in<br>-Willott, E., et al. "Immunochemical identification of insect hemocyte populations: monoclonal antibodies distinguish four major hemocyte types in manduca sexta." European Journal of Cell Biology 65.2 (1994): 417-423.<br>-Wiegand, Claudia, et al. "Monoclonal antibody MS13 identifies a plasmatocyte membrane protein and inhibits encapsulation and spreading reactions of Manduca sexta hemocytes." Archives of Insect Biochemistry and Physiology: Published in Collaboration with the Entomological Society of America 45.3 (2000): 95-108.

-Nardi, James B., Chenhua Gao, and Michael R. Kanost. "The extracellular matrix protein lacunin is expressed by a subset of hemocytes involved in basal lamina morphogenesis." Journal of Insect Physiology 47.9 (2001): 997-1006.
-Beetz, Susann, Marion Brinkmann, and Tina Trenczek. "Differences between larval and pupal hemocytes of the tobacco hornworm, Manduca sexta, determined by monoclonal antibodies and density centrifugation." Journal of insect physiology 50.9 (2004): 805-819.
-Levin, David M., et al. "A hemocyte-specific integrin required for hemocytic encapsulation in the tobacco hornworm, Manduca sexta." Insect biochemistry and molecular biology 35.5 (2005): 369-380.
-Beetz, Susann, et al. "Correlation of hemocyte counts with different developmental parameters during the last larval instar of the tobacco hornworm, Manduca sexta." Archives of Insect Biochemistry and Physiology: Published in Collaboration with the Entomological Society of America 67.2 (2008): 63-75.

The M. sexta granular cell specific monoclonal antibody MS#7 is extensively validated in:
-Willott, E., et al. "Immunochemical identification of insect hemocyte populations: monoclonal antibodies distinguish four major hemocyte types in manduca sexta." European Journal of Cell Biology 65.2 (1994): 417-423.
-Beetz, Susann, Marion Brinkmann, and Tina Trenczek. "Differences between larval and pupal hemocytes of the tobacco hornworm, Manduca sexta, determined by monoclonal antibodies and density centrifugation." Journal of insect physiology 50.9 (2004): 805-819.
-Beetz, Susann, et al. "Correlation of hemocyte counts with different developmental parameters during the last larval instar of the tobacco hornworm, Manduca sexta." Archives of Insect Biochemistry and Physiology: Published in Collaboration with the Entomological Society of America 67.2 (2008): 63-75.
-von Bredow, C. R., and T. E. Trenczek. "Distinguishing Manduca sexta haemocyte types by cytometric methods." Invertebrate Survival Journal (2022): 13-27.

The oenocytoid labelling anti-M. sexta β 1,3-glucan recognition protein 1 immune serum is validated in:
-Ma, Congcong, and Michael R. Kanost. "A β1, 3-glucan recognition protein from an insect, Manduca sexta, agglutinates microorganisms and activates the phenoloxidase cascade." Journal of Biological Chemistry 275.11 (2000): 7505-7514.
-von Bredow, Christoph-Rüdiger, Yvette M. von Bredow, and Tina E. Trenczek. "The larval haematopoietic organs of Manduca sexta (Insecta, Lepidoptera): An insight into plasmatocyte development and larval haematopoiesis." Developmental & Comparative Immunology 115 (2021): 103858.

The spherule cell labelling anti-Ephestia kuehniella haemolymph esterase immune serum is validated for the spherule cells of M. sexta in:
-von Bredow, Christoph-Rüdiger, Yvette M. von Bredow, and Tina E. Trenczek. "The larval haematopoietic organs of Manduca sexta (Insecta, Lepidoptera): An insight into plasmatocyte development and larval haematopoiesis." Developmental & Comparative Immunology 115 (2021): 103858.

The polyclonal rabbit anti-DUOX antibody was custom-created by Davids Biotechnologie (Fig. S16). One rabbit was injected with the DUOX peptide LLRDKHCRYGKAPGGHDAIR (amino acids 342–361, within the extracellular PHD of DUOX). The affinity-purified antibody was used at a dilution of 1:100 (v/v) for Western blot and immunohistochemistry. The preimmune serum was tested as an additional control.

Animals and other organisms

Policy information about studies involving animals; ARRIVE guidelines recommended for reporting animal research

Laboratory animals	The Manduca sexta larvae were obtained from our in-house colony, which has now existed for over 25 years at the University of Giessen. Only animals of the same developmental stage (L5 larvae, day 5–6 if not indicated otherwise) were included in this study.
Wild animals	This study does not involve wild animals.
Field-collected samples	The study did not involve samples collected from the field.
Ethics oversight	No ethical approval or guidance was required to use Manduca sexta as a model system.

Note that full information on the approval of the study protocol must also be provided in the manuscript.
